# The Progress and Promise of RNA Medicine—An Arsenal of Targeted Treatments

**DOI:** 10.1021/acs.jmedchem.2c00024

**Published:** 2022-05-09

**Authors:** Janet M. Sasso, Barbara J. B. Ambrose, Rumiana Tenchov, Ruchira S. Datta, Matthew T. Basel, Robert K. DeLong, Qiongqiong Angela Zhou

**Affiliations:** ∥ CAS, a division of the American Chemical Society 2540 Olentangy River Road, Columbus, Ohio 43202, United States; ‡ College of Veterinary Medicine, Kansas State University, Manhattan, Kansas 66506, United States; § Nanotechnology Innovation Center Kansas State, Kansas State University, Manhattan, Kansas 66506, United States

## Abstract

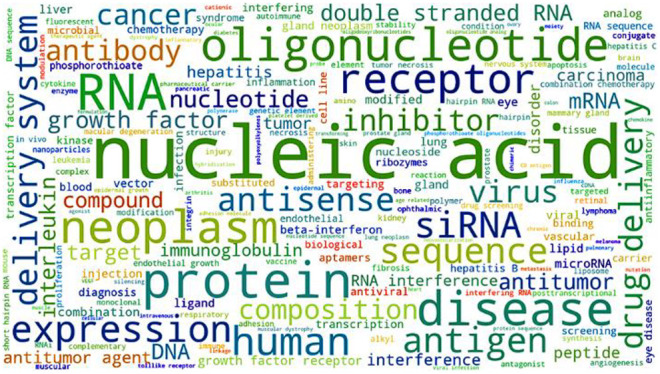

In the past decade, there has been a shift in research, clinical development, and commercial activity to exploit the many physiological roles of RNA for use in medicine. With the rapid success in the development of lipid–RNA nanoparticles for mRNA vaccines against COVID-19 and with several approved RNA-based drugs, RNA has catapulted to the forefront of drug research. With diverse functions beyond the role of mRNA in producing antigens or therapeutic proteins, many classes of RNA serve regulatory roles in cells and tissues. These RNAs have potential as new therapeutics, with RNA itself serving as either a drug or a target. Here, based on the CAS Content Collection, we provide a landscape view of the current state and outline trends in RNA research in medicine across time, geography, therapeutic pipelines, chemical modifications, and delivery mechanisms.

## Introduction

Recent advances in RNA design and delivery have enabled the development of RNA-based medicine for a broad range of applications, including therapeutics, vaccines, and diagnostics. While human RNA medicine has faced many challenges in terms of efficacy and immunogenicity, the recent success of mRNA vaccines against COVID-19 and the approval of new RNA-based drugs provide new momentum to the field. Many classes of RNA play important regulatory roles in cells and tissues, beyond the obvious role of mRNA in protein synthesis. Scientific research, clinical development, and commercial production now focus on exploiting the many roles of RNA for use in biotechnology and medicine. Advances in understanding RNA structure and function are combined with a robust production pipeline to develop clinically effective RNA-related applications.^1−12^

**Figure 1:**
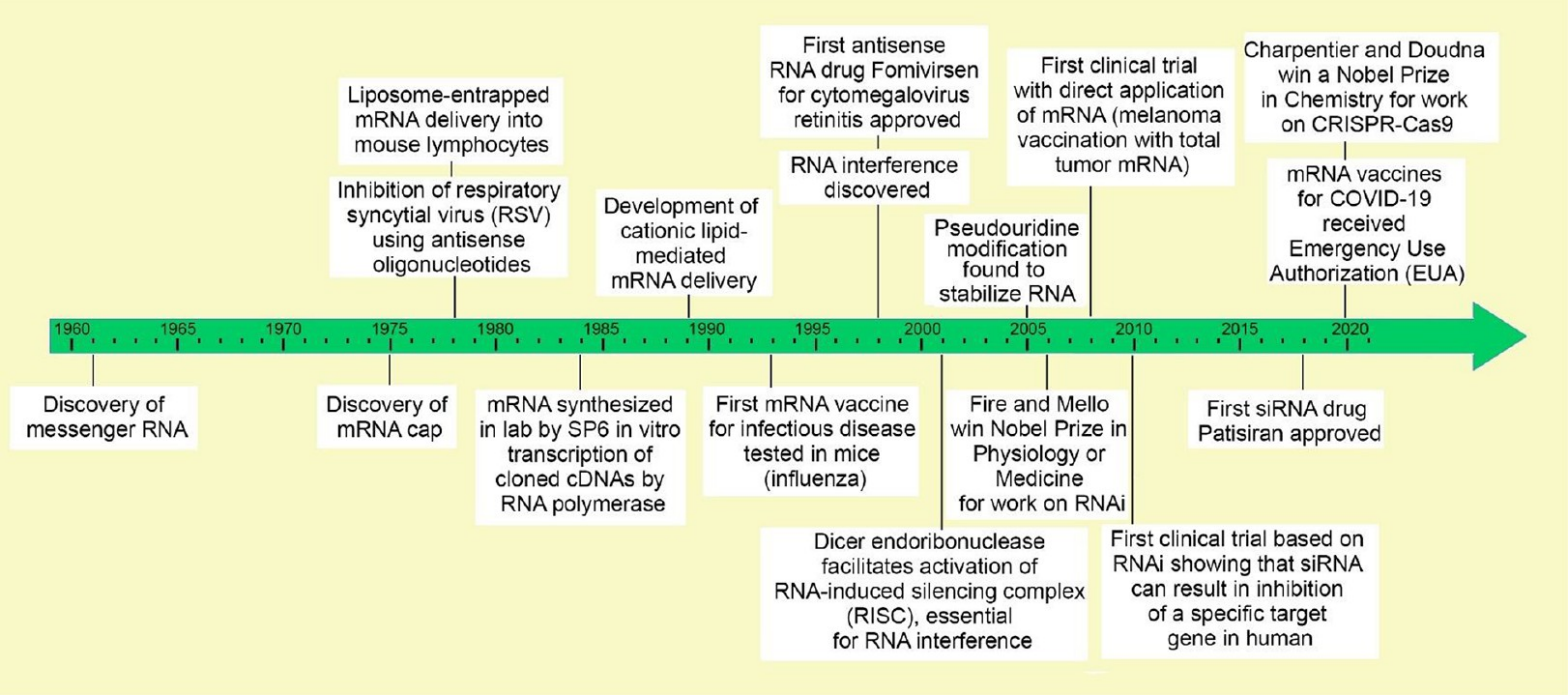
Timeline of major RNA research and development milestones. A more detailed timeline table complete with references is provided as Table S1.

Many key discoveries have contributed to the advancing of RNA medicines we have today. Early research in the 1960s on nucleic acids led to the discovery of mRNA.^13^ In the next decade, the 5′-cap on mRNA was discovered,^14,15^ the first liposome-entrapped RNA was delivered into cells,^16^ and antisense oligomers (ASOs) were used to inhibit Rous sarcoma virus (RSV).^17^ In the 1980s, *in vitro* transcription from engineered DNA templates using a bacteriophage SP6 promoter and RNA polymerase^18^ allowed the manufacture of mRNA and expression of other types of RNA in cell-free systems. Later in the 1980s, the first cationic-lipid-mediated mRNA delivery was achieved.^19,20^ The discovery of RNA interference (RNAi)^21^ and the approval of the first antisense RNA drug in the late 1990s^22^ were key to the development of RNA therapeutics. For their pioneering work on RNAi and the RNA-induced silencing complex (RISC),^23^ Fire and Mello^21^ were awarded the Nobel Prize in Physiology and Medicine in 2006. During the 2000s, the discovery of the importance of pseudouridine modification^24^ and further research on mRNA led to the first human trial of an mRNA vaccine against melanoma in 2008.^25^ In 2010, a pivotal human clinical trial showed that siRNA could target specific human genes,^10^ and subsequent pre-clinical research and development led to the approval of the first siRNA drug in 2018.^26^ Most recently, Doudna and Charpentier were awarded the Nobel Prize in 2020 for CRISPR-Cas9 gene editing. In a CRISPR-Cas9 system, a small piece of RNA with a short “guide” sequence attaches to a specific target sequence of DNA in a genome; the RNA also binds to the Cas9 enzyme—thus, the modified RNA is used to recognize the DNA sequence, and the Cas9 enzyme cuts the DNA at the targeted location.^27^ Two human mRNA vaccines against COVID-19 received Emergency Use Authorization in 2020, and one of them was finally approved in 2021.^28−30^ These key milestones and achievements are captured in Figure 1.

RNA technology provides an innovative approach for developing new drugs for rare or difficult-to-treat diseases. Since 2014, several drugs have been approved to treat macular degeneration, Duchenne muscular dystrophy (DMD), polyneuropathy, and amyotrophic lateral sclerosis.^31^ Drugs to treat many other diseases, including cancer, hepatic and renal diseases, cardiac diseases, metabolic diseases, blood disorders, respiratory diseases, and autoimmune diseases are currently in various stages of clinical studies, some showing promising results.

Compared to other biomolecules, RNA molecules are unstable and transient. Foreign RNA molecules, when introduced to the human body, have limited protein expression levels in cells and often trigger immunogenicity in the body. However, these practical problems can often be mitigated by various optimizations on RNA molecules, including chemical modifications, mRNA cap/codon/tail optimization, etc. Leveraging the unique aspects of both the chemical and biological information of the CAS Content Collection,^32^ this paper focuses on chemical modifications to the base, backbone, sugar, and 5′ or 3′ ligations to other molecules. While chemical modifications increase RNA stability, complexes of RNA within nanoparticles provide further protection. Except for aptamers that bind to a cell surface target, RNA must be delivered into the cell by a carrier. After the RNA is internalized, it must be released from the membrane-bound vesicle or endosome to the cytosol. Without a doubt, the delivery system has been an important research topic for RNA medicine.

In this paper, we reviewed naturally occurring RNAs with their cellular functions and the associated research trends based on the analysis of CAS Content Collection.^32^ We then appraised different types of RNA in medical applications: their advantages, challenges, and research trends. Subsequently, we assessed the development pipelines of RNA therapeutics and vaccines with company research focuses, disease categories, development stages and publication trends. Finally, we discussed RNA chemical modifications and delivery systems in detail, as they are critical to the success of RNA medicine. We thus revealed the sustained global effort that propelled this field to the cusp of realization for novel medical applications of RNA in many diseases. We hope this review can serve as an easy-to-understand overview so that scientists from many different disciplines can appreciate the current state of the field of RNA medicine and join in solving the remaining challenges for fulfilling its potential.

## Types of Naturally Occurring RNA and Their Functions in Biological Systems

**Figure 2:**
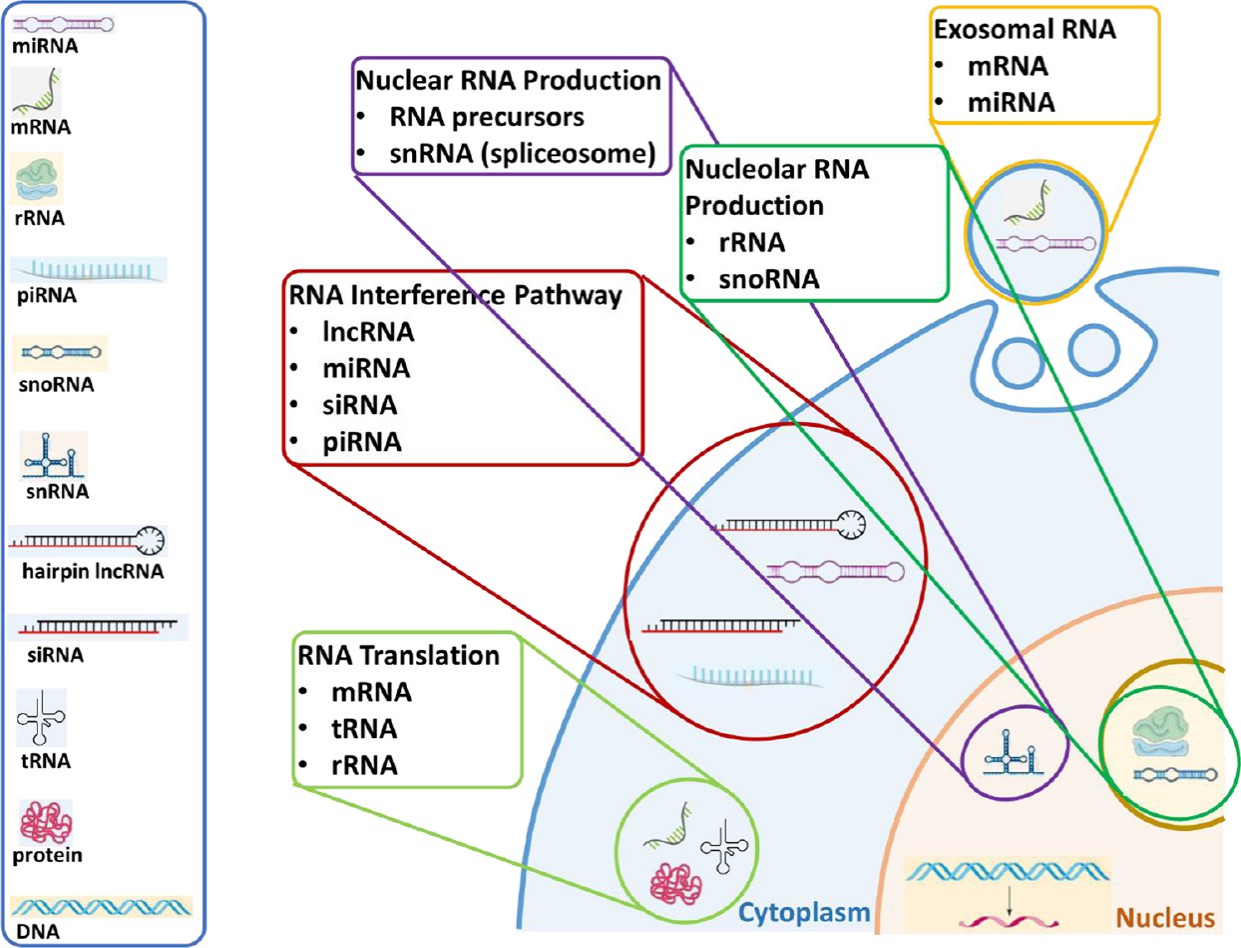
Types of naturally occurring RNA and their cellular functions and localizations

RNA, a versatile macromolecule that is specialized for many functions, can be broadly defined as coding or messenger RNA (mRNA) and non-coding RNA (ncRNA). There are several different types of ncRNA including ribosomal RNA (rRNA),^33,34^ transfer RNA (tRNA),^33,34^ small nuclear RNA (snRNA),^35−37^ small nucleolar RNA (snoRNA),^36,38−45^ long non-coding RNA (lncRNA),^7,46−52^ short hairpin RNA (shRNA), micro-RNA (miRNA),^53−59^ transfer messenger RNA (tmRNA), small interfering RNA (siRNA),^60−71^ small activating RNA (saRNA), piwi-interacting RNA (piRNA),^3,4,72−78^ circular RNA (circRNA), ribozymes, and exosomal RNA.^79−83^ The cellular localizations of different types of RNA are illustrated in Figure 2, and an overview of their functions is provided in the following section.

### Functions of the Naturally Occurring RNA Types

**mRNA**, which was the first RNA to be characterized, is the initial transcription product of a protein-coding gene and includes both protein-coding exons and non-coding introns. The mature, translatable mRNA must be spliced to remove the introns and, in transcripts that can go through alternative splicing, one or more potential exons. The mature RNA has a 5′-7-methylguanosine cap, a 5′-untranslated region, a start codon (unique sequence of 3 bases) for the translated region of the gene, a stop codon that ends the translated region of the gene and starts the 3′-untranslated region, and a 3′-polyadenosine tail. Gene expression, which is often regulated by the amount of mRNA for the gene, is controlled by the balance between synthesis and degradation of mRNA. Although mRNA is a critically important RNA, it makes up only 1–5% of the RNA in a cell.^33,34^

**ncRNAs**, in contrast to mRNA, are the final functional products of the DNA. Although it was thought initially that ncRNAs were non-functional junk RNA, in the 1950s, in the same paper that introduced the phrase “Central Dogma”, Francis Crick correctly hypothesized that the ncRNA might function in the translation of mRNA into protein.^84^ In 1955, George Palade identified ribosomes as a small particulate component of the cytoplasm that contains RNA, and in 1965, Robert Holley purified a tRNA from yeast and determined the structure.^85,86^ In the past half-century, many types of ncRNA with various functions have been identified; many are involved in regulating transcription and protein expression in the cell.^87−94^

**rRNA**, which constitutes up to 80% of the RNA in an active cell, comprises three rRNAs (the 5S, 5.8S, and 28S) complexed with many proteins to form the large subunit of the ribosome and one rRNA (the 18S) complexed with proteins to form the small subunit of the ribosome. There are also two mitochondrial rRNA genes (the 12S and 16S) which, along with many proteins, form the mitochondrial ribosome. rRNA in the ribosome, acting as a ribozyme, catalyzes peptide bond formation between two amino acids. Synthesis of the large amount of rRNA occurs in the nucleolus, a heterochromatic region found in most nuclei.^33,34^

**tRNA**, which makes up 10–15% of the RNA in the cell, translates the mRNA codon sequence for each amino acid. The many different tRNAs, which are usually 75–95 nucleotides, all fold into very similar three-dimensional structures. The 3D structure exposes three unpaired nucleotides that serve as the anti-codon to base pair with the mRNA. Specific amino acids are covalently bound to a tRNA by aminoacyl tRNA synthetases. The specificity between the anti-codon and the bound amino acid is the basis of translation. Each tRNA anticodon can bind to several different mRNA codons. This pairing is based on the wobble rules for the third nucleotide position of the anti-codon, which is often post-transcriptionally modified to allow for wobble-pairing. Common modifications at the third position include 5-methyl-2-thiouridine, 5-methyl-2′-O-methyluridine, 2′-O-methyluridine, 5-methyluridine, 5-hydroxyuridine, hypoxanthine, and lysidine.^33,34^

**snRNAs** (∼150 nucleotides) are components of the small nuclear ribonucleoproteins (snRNPs) of the spliceosome. They act as catalysts that splice mRNA into its mature form and they are important in the selection of alternative splicing sequences.^35−37^

**snoRNAs** (60–300 nucleotides) are bound to four core proteins and act as guides to correctly target modifications for the maturation of rRNA. They comprise two classes of RNA. C/D snoRNAs participate in the 2-methylation of targeted nucleotides, while H/ACA snoRNAs participate in the modification of uridine to pseudouridine. They help guide the protein to the specific target, rather than catalyzing the reaction directly. As participants in rRNA maturation, snoRNAs are found in the nucleolus.^36,38−45^

**siRNAs** are products of double-stranded lncRNAs (e.g., hairpin lncRNA) and are central to RNA interference, which negatively regulates gene expression. Double-stranded RNA (dsRNA), either from genomic lncRNA or dsRNA viruses, is recognized and cleaved by the endonuclease Dicer into 20–24 base-pair sections with short overhangs on both ends. These siRNAs bind the Argonaute protein to form the pre-RISC (RNA-induced silencing complex). Argonaute selects the less thermodynamically stable strand of the siRNA and releases the other strand to form the mature RISC. RISC recognizes mRNA complementary to the single-stranded siRNA, and the Argonaute endonuclease cuts this targeted mRNA, thereby downregulating the gene product. The binding of a RISC to a target mRNA also prevents efficient ribosome binding and translation, further downregulating the gene product. Active RISCs may also affect the transcription of target genes by inducing chromatin reorganization through epigenetic modifications. This can be a defense mechanism against dsRNA viruses or an endogenous gene-regulatory mechanism.^60−71^

**miRNAs** are closely related to siRNAs but are formed from pri-miRNAs (primary microRNAs), which are long, imperfectly paired hairpin RNA transcripts. The pri-miRNA is processed first by Drosha nuclease into a ∼70-nucleotide imperfectly paired hairpin pre-miRNA (precursor microRNA) that is then, like siRNA, processed by Dicer to produce the 21–23-bp, mature, double-stranded miRNA that binds to Argonaute to form the RISC. Alternatively, some miRNAs are made from introns in mRNAs. After splicing, that intron is a pre-miRNA that is processed by Dicer to form a RISC. miRNAs form negative gene regulatory networks and intronic miRNAs may regulate and balance potentially competing pathways.^53−59^

**piRNAs**, like siRNA and miRNA, negatively regulate gene expression, but they interact with the Piwi class of Argonaute proteins. Unlike siRNA and miRNA, piRNAs (24–31 nucleotides) are produced from long, single-stranded RNA transcripts through an uncharacterized Dicer-independent mechanism. Mature piRNAs bind to Piwi proteins to form RISCs that act primarily as epigenetic regulators of transposons (genetic elements that move around the genome) but may also regulate transposons post-transcriptionally through the ping-pong pathway.^3,4,72−78^

**saRNA**, like siRNA, is a ∼21-bp dsRNA long that interacts with Argonaute proteins to form a RISC. Unlike siRNA, saRNA upregulates target gene expression by an unknown mechanism, perhaps activating transcription by targeting the promoter region of the gene. saRNA may be produced endogenously or artificially to strongly activate the target gene.^5,95−101^

**lncRNAs** comprise a mixed group of RNAs >200 bp, which differentiates them from short ncRNAs such as snoRNA, siRNA, miRNA, piRNA, etc. lncRNAs have a wide variety of functions including regulation of chromosome architecture and interactions, chromatin remodeling, and positive or negative regulation of transcription, nuclear body architecture, and mRNA stability and turnover.^7,46−52^

**circRNAs** are lncRNAs with 5′ and 3′ ends linked covalently to form a continuous circle. circRNAs are broadly expressed in mammalian cells and have shown cell-type and tissue-specific expression patterns.^102^ Neither the mechanisms leading to circularization of the RNA nor the function of circRNA is known, but the leading hypothesis is that they may serve as miRNA sponges. Many circRNAs contain large numbers of miRNA target sites that may competitively antagonize the ability of miRNA to silence its target genes.

**Exosomal RNAs** are mRNAs, miRNAs, siRNAs, and lncRNAs that are packaged and exported from the cell through the exosomal pathway. Although they are poorly understood, exosomal RNAs may serve as signaling molecules to regulate gene expression in target cells. These circulating RNAs, especially miRNAs, may serve as diagnostic and/or prognostic targets for various diseases such as cancers.^79−83^

**Antisense RNA** (asRNA), also referred to as antisense oligonucleotide (ASO), is a single-stranded RNA that is complementary to a protein-coding messenger RNA (mRNA) with which it hybridizes, and thereby blocks its translation into protein.^103^

### Research Trends on Different Types of RNA As Reflected by Number of Publications

**Figure 3:**
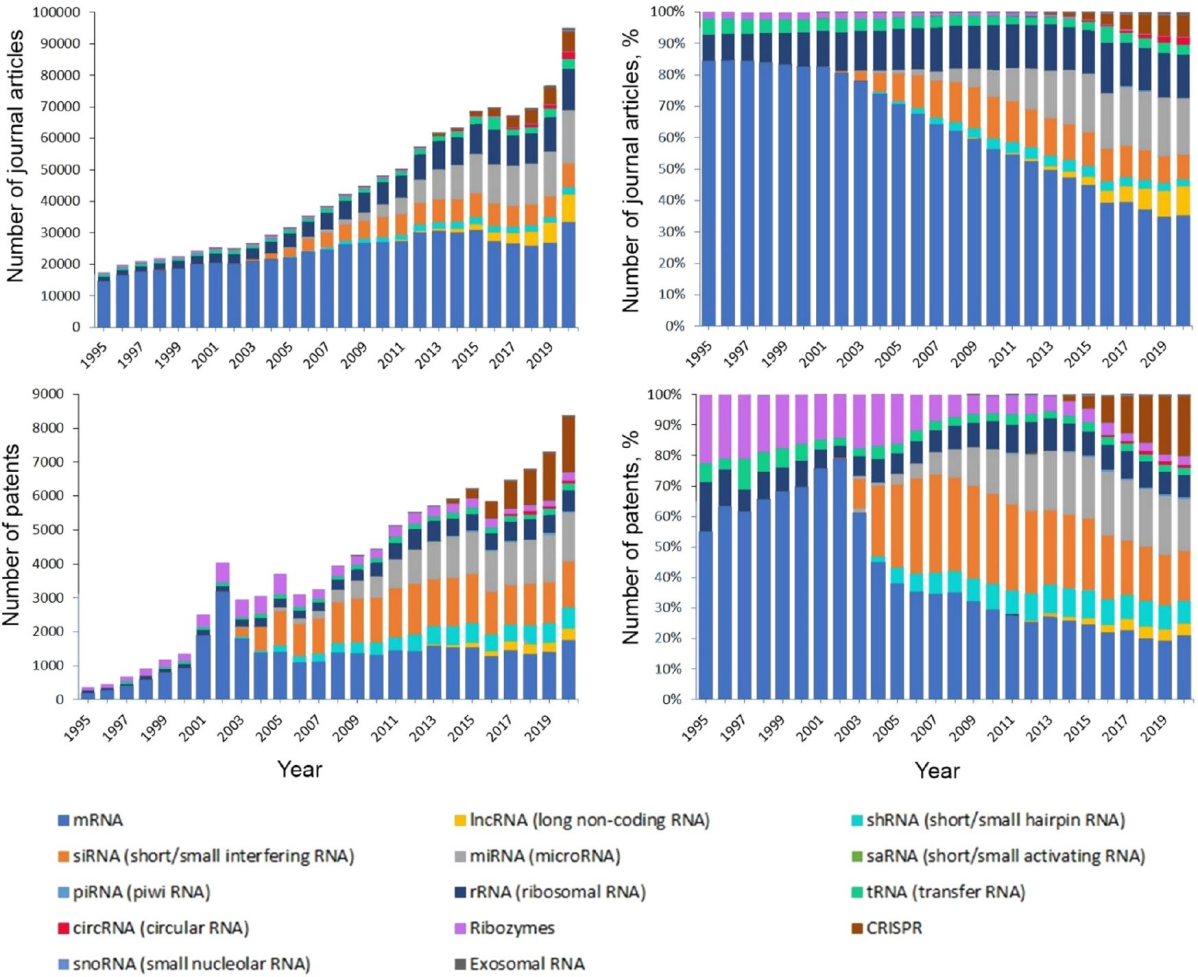
Document publication trends for different types of RNA from 1995 to 2020 as found from the CAS Content Collection.^32^ Top two panels: journal publications in absolute numbers and given year percentages. Bottom two panels: patent publications (counted once per patent family) in absolute numbers and given year percentages.

The CAS Content Collection^32^ is the largest human-curated collection of published scientific knowledge, used for quantitative analysis of global scientific publications against variables such as time, research area, formulation, application, disease association, and chemical composition. We searched the title, abstract, or CAS-indexed terms using RNA-related keywords and their synonyms to identify relevant published documents. Figure 3 shows trends in the number of publications for specific types of RNA. In the past 25 years, the research areas became more diverse as new types of RNA were discovered, and this is reflected in both journal and patent publications, particularly in the areas of siRNA, miRNA, lncRNA, and CRISPR-related research. CRISPR technology has recently increased rapidly in volume of patent publications, and it accounted for 20% of the RNA-related patent publications in 2020.

**Figure 4:**
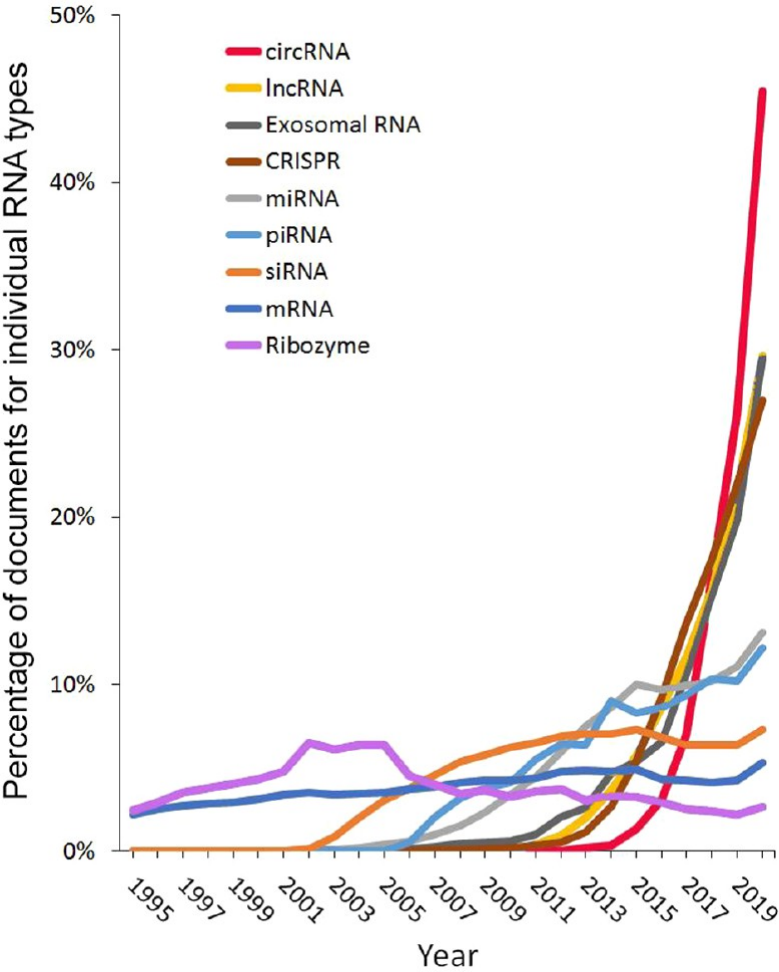
Trends in publication volume for different RNA types in the years 1995–2020. Percentages are calculated with yearly publication numbers for each individual RNA type, normalized by total publications in the years 1995–2020 for the same RNA type. Example: Percentage of circRNA documents in 2020 = (number of circRNA documents in 2020)/(total number of circRNA documents from 1995 to 2020).

To better reveal the rising trends of those recently emerged types of RNA, the percentage of document publications of a specific year was calculated within the given type of RNA over the time (Figure 4). Although the cumulative publication numbers for circRNA, exosomal RNA, lncRNA, and CRISPR, are relatively small compared with others (Figure 3), their rates of increase are much faster.

## Types of RNA Used in Medical Applications, Their Advantages, and Challenges

### Types of RNAs and Their Applications in Medicine

mRNA transcripts can act as therapeutic RNAs, diagnostic biomarkers, or therapeutic targets. Translation of an mRNA in the cell can produce a therapeutic protein to replace a defective or missing protein. In the case of vaccines, mRNA translation can generate antigenic targets for the immune system, such as the spike glycoprotein of SARS-CoV-2 in the COVID-19 mRNA vaccines. mRNA may also serve as a therapeutic target for ASOs, siRNA, miRNA, aptamers, and suppressor tRNAs.

In the cell, miRNAs bind to the 3′-untranslated region of mRNAs and target them for degradation by the RISC.^104^ Because a single miRNA binds to multiple mRNAs, miRNAs serve as regulatory check points. The cellular processes regulated by miRNAs include those involved in many diseases, such as cardiovascular disease, cancer, and disease-related metabolic pathways.^105,106^ Thus, they can serve as biomarkers for disease diagnosis, as potential drugs, or as attractive targets for other regulatory RNAs.

Although siRNAs, like miRNAs, use the RISC to degrade their target mRNAs, siRNAs bind to specific areas in the mRNA coding region. This target specificity makes them attractive as potential drugs, but off-target effects can negate this advantage. In order to minimize off-target effects, siRNAs are modified to decrease their thermal stability, increase their target specificity, and decrease the stability of their binding of the siRNA to mRNAs that are not an exact match to the intended target mRNA. These usually include 2′-O-methyl and 2′-MOE ribose modifications that introduce steric hindrance to decrease binding affinity (more discussion in the Chemical Modifications section below).^104^

**Figure 5:**
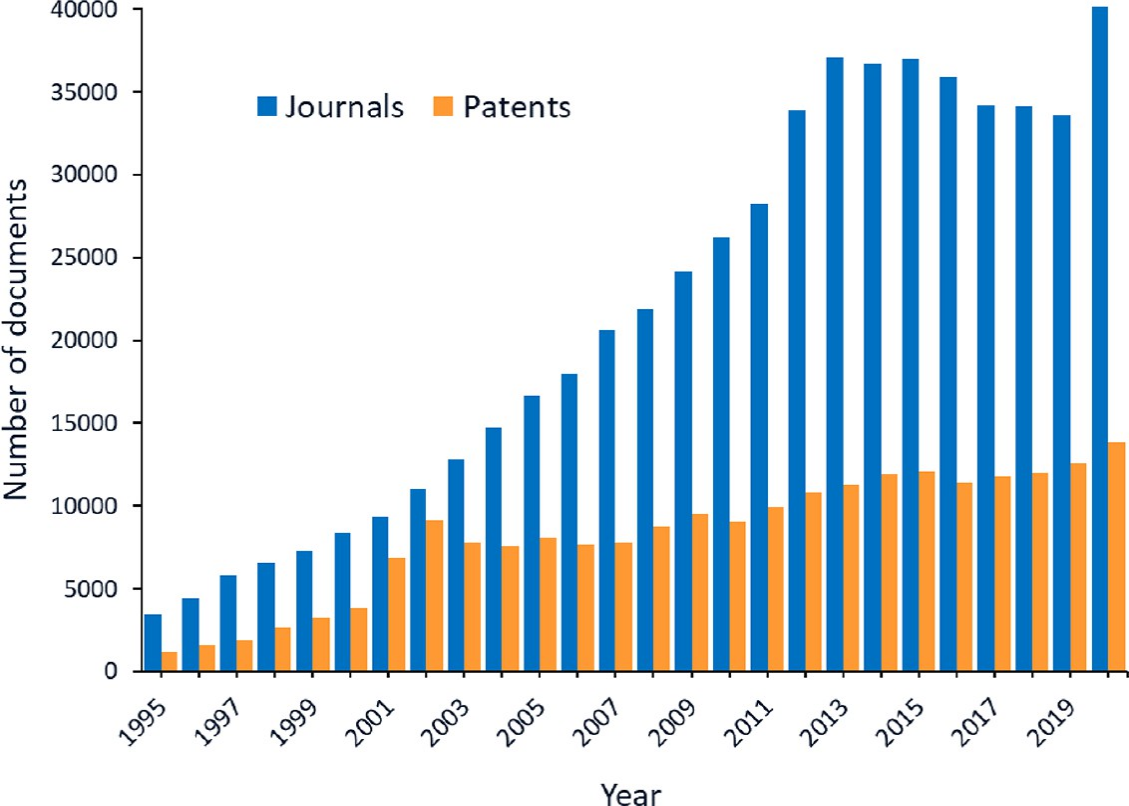
Numbers of journal documents and patents related to RNAs for medical use by year.

One of the earliest therapeutic RNAs, ASOs, recognize and bind to complementary DNA or RNA sequences, including mutated sequences that may lead to disease. Upon binding to the mutated sequences, ASOs may facilitate proper mRNA splicing, prevent translation of a defective protein, or target RNAs for degradation.^104^

In therapeutic antibody–oligonucleotide conjugates (AOCs), the antibody targets the site of interest while carrying an ASO or a siRNA that acts on the targeted region.^107^ Conjugation of an ASO to an antibody to create an AOC improves the pharmacokinetics of the ASO *in vivo* by increasing tissue distribution and prolonging gene silencing in multiple tissues.^108^

The CRISPR-Cas system uses a guide RNA that is either a combination of a trans-activating CRISPR RNA (tracrRNA) and a CRISPR RNA (crRNA) or a joined single guide RNA (sgRNA). The guide RNA directs the CRISPR complex containing a Cas endonuclease to a specific site in the genome for cleavage.^6^ The ability of the CRISPR-Cas system to create directed double-stranded breaks in DNA allows the repair of genetic mutations. Changing the endonuclease activity of Cas can convert CRISPR-Cas to a system that nicks a single strand of the DNA or that deaminates a specific nucleotide.^109^ If the Cas endonuclease is inactivated, the system can simply bind to DNA to regulate transcription.^104^

Aptamers are structure-based rather than sequence-based ligands that neither hybridize with other nucleic acids nor produce proteins. They can be RNA, DNA, RNA/DNA combinations, or even proteins. *In vitro* systematic evolution of ligands by exponential enrichment (SELEX) is used to identify single-stranded RNA or DNA oligonucleotides with a high affinity for a target. Because their binding depends on their 3D structure, aptamers can bind a wide range of targets, including proteins, cells, microorganisms, chemical compounds, and other nucleic acids.^110^ Aptamers may also serve as delivery agents for siRNA in nanoparticles for cancer therapy.^111^

**Figure 6:**
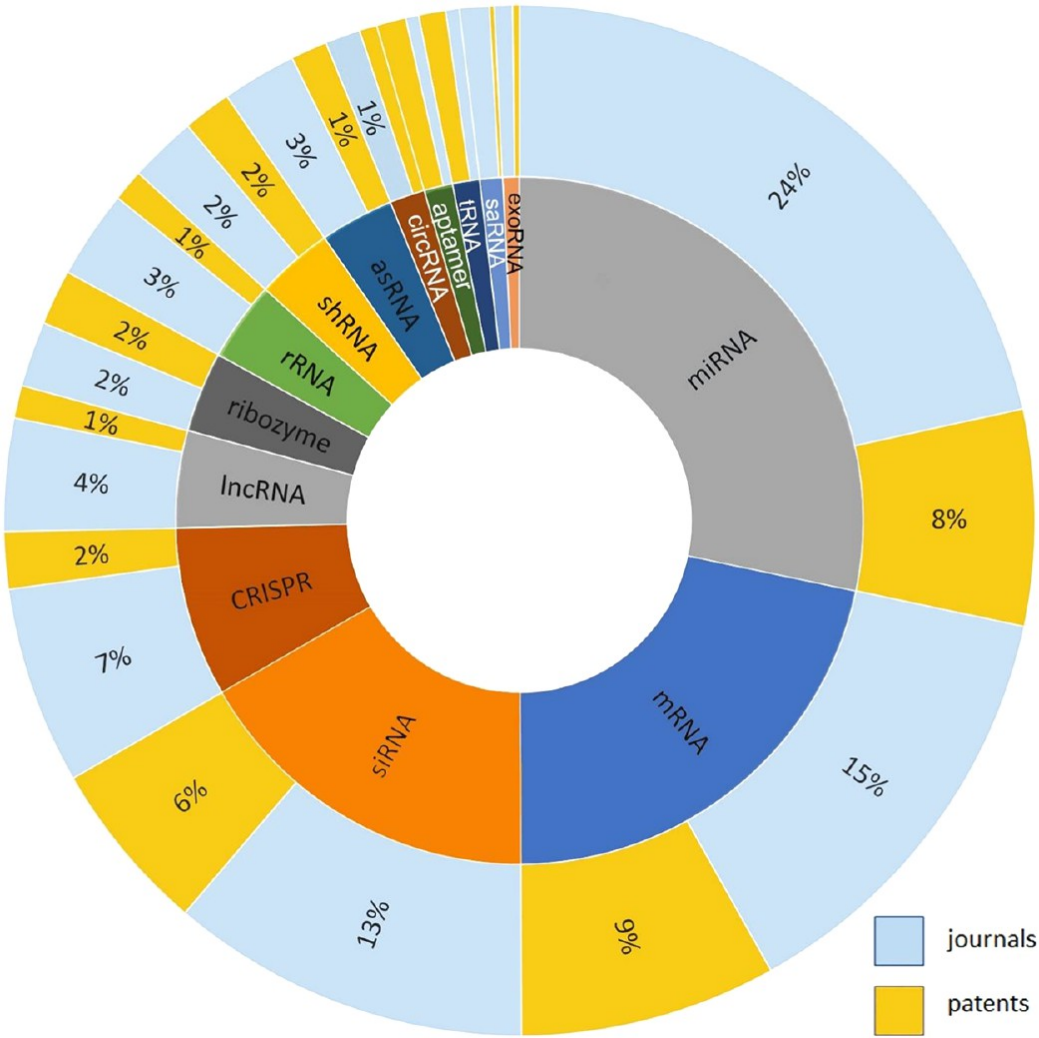
Percentage of journal documents and patents for various types of RNA used in medical studies including therapeutics, vaccines, and diagnostics.

To measure the distribution of research effort using different types of RNA as therapeutics, vaccines, or diagnostics, related documents were extracted accordingly from the CAS Content Collection, and journal and patent publications percentages for each type of RNA were determined as shown in Figure 6.^32^ miRNA and mRNA, the two most popular therapeutic RNAs in the journal and patent literature, can serve as drugs, disease biomarkers, and drug targets. Together with siRNA, they represent most of the therapeutic RNA patent activity. Approved RNA drugs include mRNAs, siRNAs, ASOs, and aptamers; these RNAs along with CRISPR RNAs and AOCs comprise most of the clinical candidates.

### Publication Trends for RNAs Used in Medical Applications

The CAS Content Collection^32^ shows a steady increase in the number of journal articles and patents related to RNA applications in medicine (Figure 5). The peak in patents in 2001–2002 may correlate with the first clinical trials using dendritic cells transfected with mRNA encoding tumor antigens (a therapeutic mRNA cancer vaccine) in 2001.^112,113^ The spike in journal article numbers in 2020 likely resulted from interest in the COVID-19 mRNA vaccines. The increase in journal articles and patents on therapeutic RNA from 2011 to 2016 can be attributed to initial interest in siRNA and miRNA, which decreased temporarily with the discovery of their off-target effects. Interest in mRNA also increased from 2011 to 2016, then decreased and only recovered once mRNA vaccines took center stage in the fight against COVID-19.

However, other types of RNA are potential therapeutics or targets. These RNAs include (a) shRNA; (b) lncRNA, an RNA whose role in gene regulation is generating increasing interest; (c) circRNA, once thought to be a byproduct of RNA splicing, that may have a role in regulation as a miRNA sponge; (d) saRNA, which regulates gene transcription; and (e) exosomal RNA, which is contained in naturally occurring lipid vesicles called exosomes, which can cross the blood-brain barrier and appear to be vital for cell-to-cell communication, and which transport mainly miRNA.^114^

**Figure 7:**
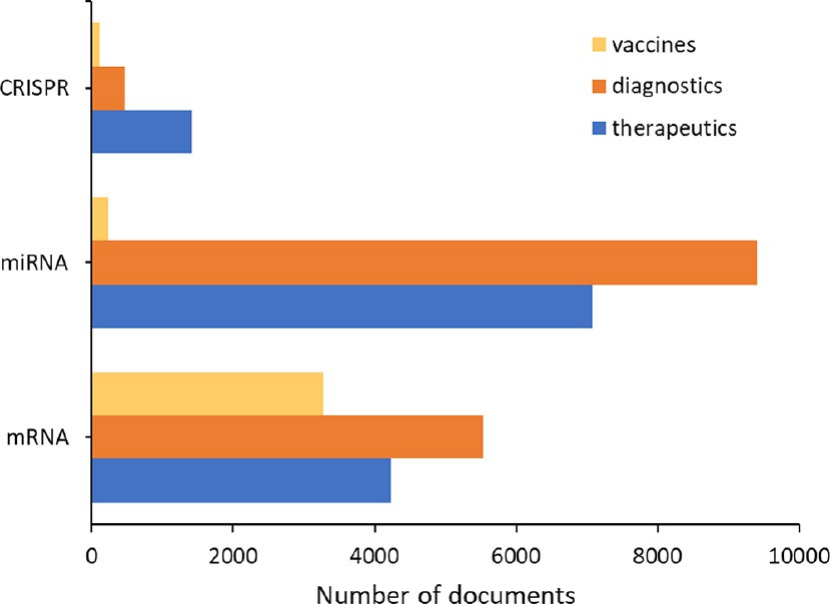
Number of journal publications and patents for mRNA, miRNA, and CRISPR with applications in therapeutics, vaccines, and diagnostics.

Based on the CAS Content Collection data for CRISPR RNA, miRNA, and mRNA used in vaccines, diagnostics, and therapeutics,^32^ only mRNA has substantial applications in vaccines (Figure 7). However, both miRNA and mRNA have demonstrated their potential as therapeutics and diagnostics.

The large body of work on miRNA and mRNA in diagnostics may be surprising since these molecules are much more susceptible to nuclease degradation than DNA. However, the essential roles of mRNA and miRNA in cellular metabolism make them excellent biomarkers for the study of normal cellular processes and the diagnosis of disease. Metabolic diseases such as cancer as well as infectious diseases can be diagnosed via miRNA biomarkers by reverse transcriptase-quantitative polymerase chain reaction (RT-qPCR) and lateral flow immunoassays.^115,116^ High-throughput sequencing of total cellular mRNA pinpoints changes in gene expression that can be used in diagnosis.^117^ The specificity of the CRISPR system for its target DNA makes it a potential tool with both diagnostic and therapeutic purposes.^118^ The number of journal publications and patents using RNA for diagnosis demonstrates its power (Figure 7); however, a comprehensive review of RNA as a diagnostic biomarker or, in the case of CRISPR RNA, as a diagnostic agent, is beyond the scope of this review. Here we focus on RNAs as therapeutic agents.

### Advantages and Challenges of RNAs as Therapeutics

There are several important advantages to RNA therapeutics. They are: (a) target specific, (b) modular with easy-to-switch sequences, (c) predictable in terms of pharmacokinetics and pharmacodynamics, (d) economical in comparison to antibodies or protein drugs since they are synthesized from widely available synthons on an automated synthesizer, and (e) relatively safe, as most of them do not alter the genome.

Proteins must be designed for synthesis from genes in plasmids, then optimized for expression and purification, and these processes may be different for each protein and challenging to optimize. In contrast, RNA can be easily synthesized and purified by established methods using commercially available reagents and equipment. Small RNAs, such as aptamers, siRNAs, miRNAs, and ASOs can be synthesized using solid-support chemistry in commercial oligonucleotide synthesizers.^119,120^*In vitro* transcription using commercial kits^121^ produces longer RNAs, i.e., mRNAs and lncRNAs. The sequence of the RNA can be changed easily, providing custom molecules targeting different proteins or genes. Thus, the development of RNA therapeutics and vaccines that target disease-specific genes or proteins is relatively fast and straightforward. This was demonstrated by the development, testing, and administration of the COVID-19 mRNA vaccines within a year of the isolation and sequencing of the SARS-CoV-2 viral genome.^108^

Most RNAs regulate transcription, post-transcriptional processing, and translation, but they do not alter the genome. The exception is the CRISPR-Cas system in which the guide RNA and Cas endonuclease can edit the genome. RNA aptamers, which mimic ligands, regulate post-translational protein activity. The rapid degradation and predictable pharmacokinetics of RNAs give them a safety advantage over gene therapies.^104^

Despite the attractiveness of the plug-and-play concept of RNA therapeutic drug design, they require testing to determine their efficacy and safety, and cell delivery is difficult because RNA is easily degraded. The therapeutic RNA must penetrate the cell membrane and escape endosomal entrapment.^104^ Although designed for specific targets, therapeutic RNAs can have off-target effects, limiting their usefulness as drugs. Several of these limitations can be mitigated by chemically modifying the RNA to increase target specificity, lower nuclease susceptibility, and improve cellular uptake.^104,108^

## Types of RNA in Medicine in the Development Pipeline and Their Targeted Diseases

### Distribution of Diseases Associated with RNA Medicine in Publications and Patents

**Figure 8:**
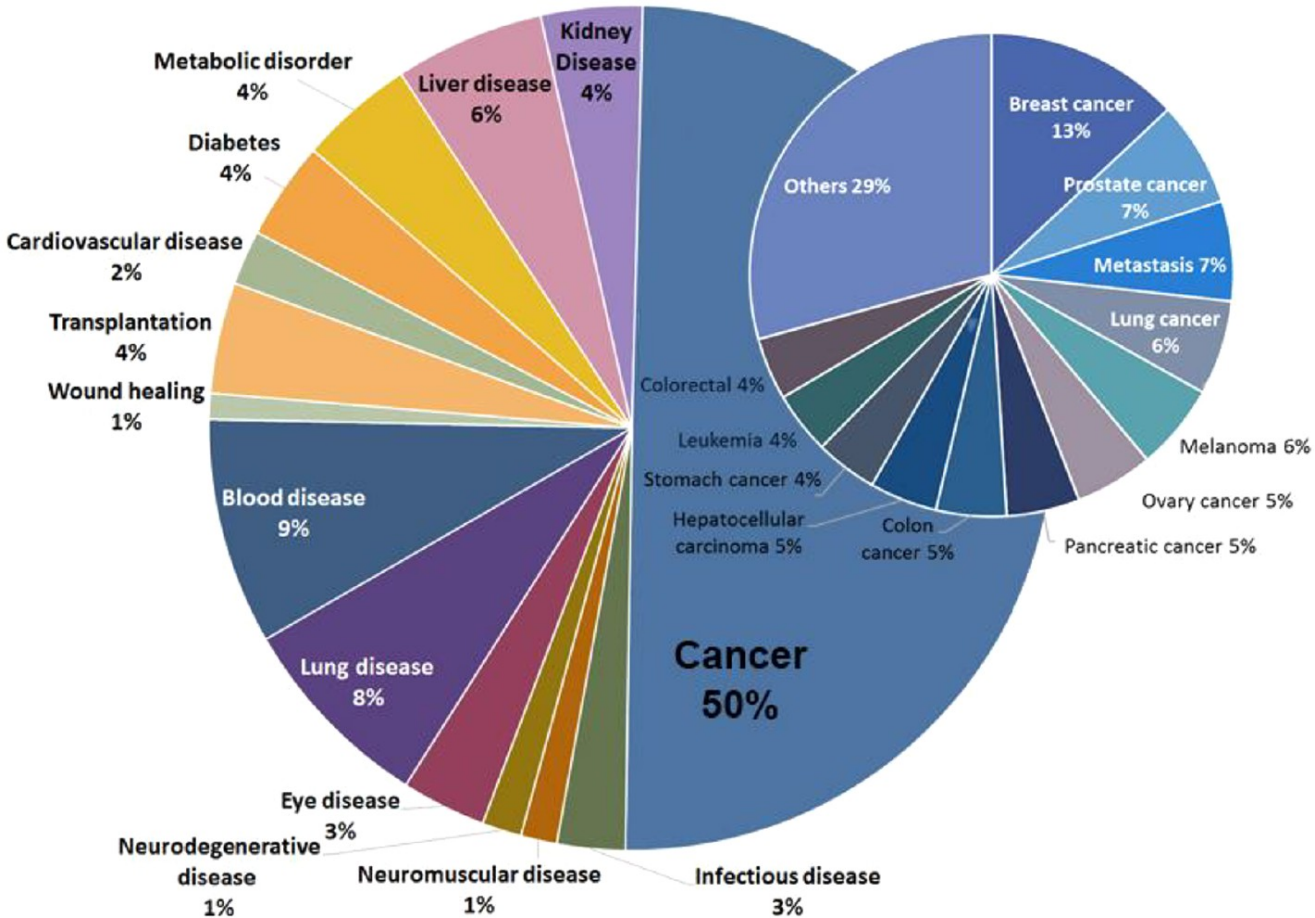
Percentage of publications associated with RNAs in medical applications

**Figure 9:**
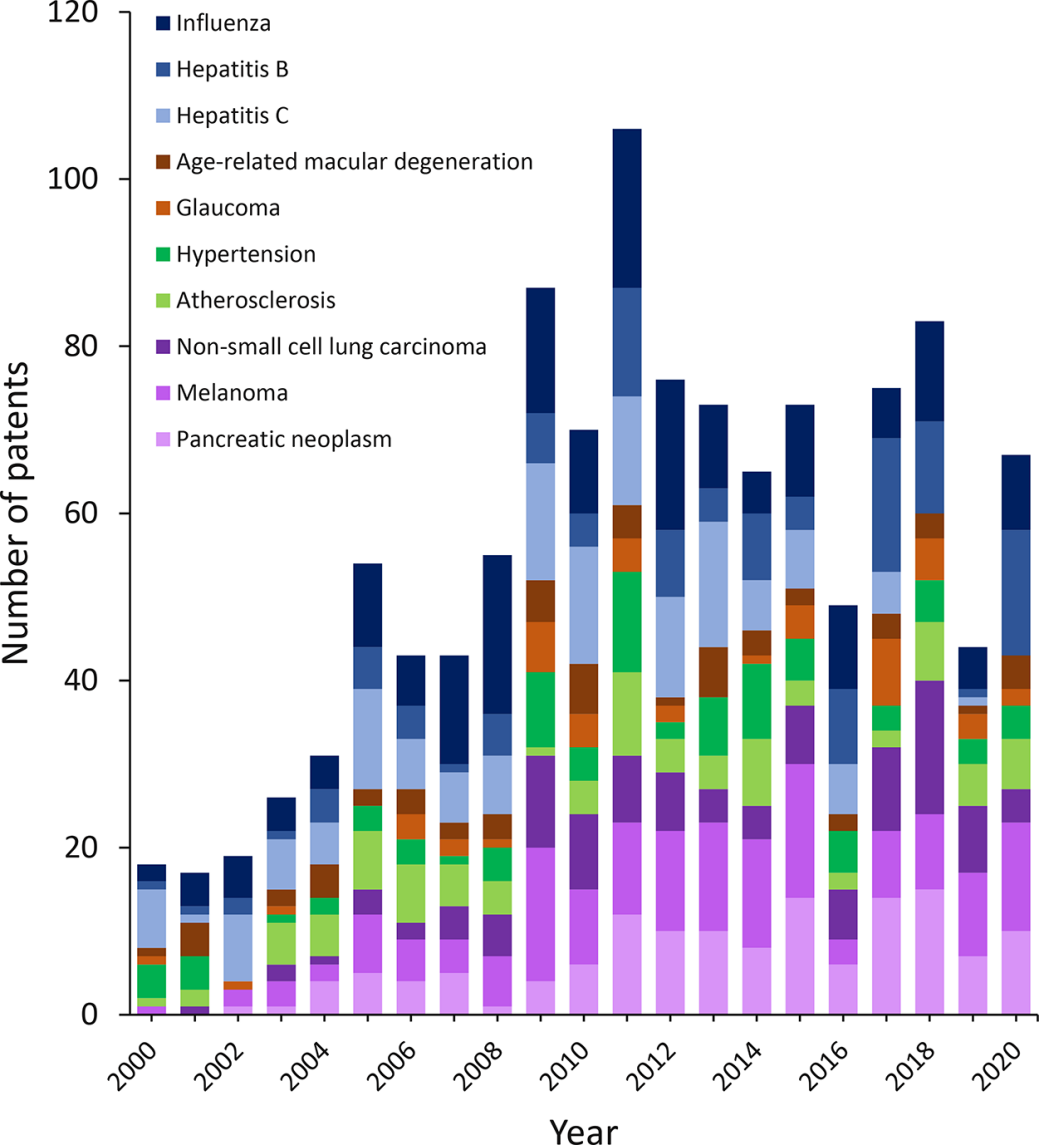
Yearly number of patent publications on specific diseases targeted by RNA therapeutics, vaccines, and diagnostics.

**Figure 10:**
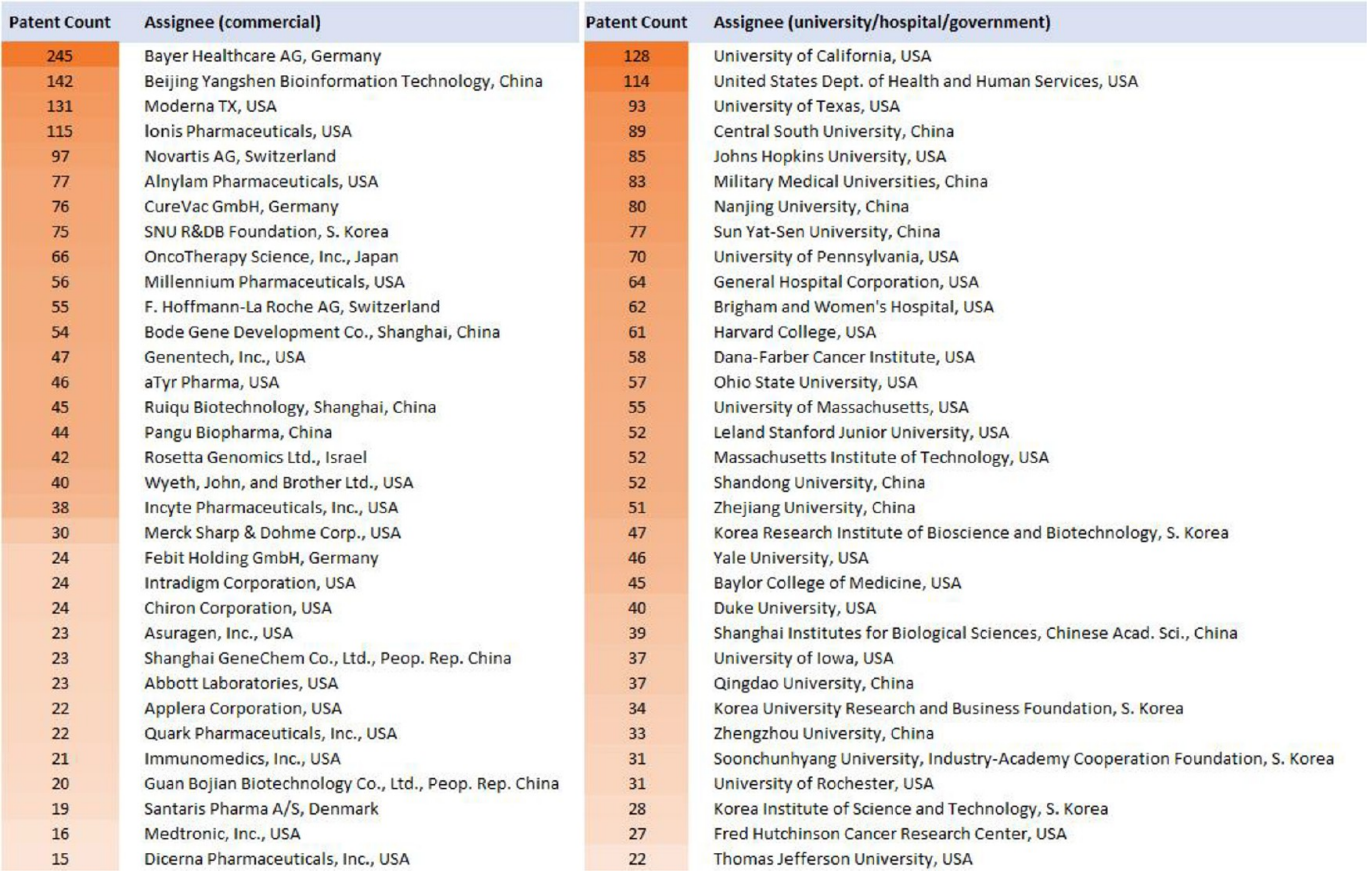
Top patent assignees for RNA therapeutics, vaccines, and diagnostics.

Since the first approved ASO RNA therapeutic in 1998, the research and development of RNA in medical applications has increased. We analyzed data from the CAS Content Collection^32^ for journal publications and patents on RNAs as therapeutics, vaccines, or diagnostic agents for diseases and found that 50% of the publications are associated with cancer diagnosis or treatment, although lung, liver, and metabolic diseases are also highly represented (Figure 8). There was little correlation between the type of RNA and a targeted disease, indicating that different types of RNA have been explored for many kinds of diseases in the research phase (Figure S1). Infectious diseases and cancer have shown the greatest growth and are the most frequent diseases treated by RNA, followed by eye and cardiovascular diseases, which grew in the first decade of this century and remained relatively stable in the second decade (Figure 9). Specifically, the association of RNA medicine with pancreatic neoplasm, melanoma, non-small-cell lung cancer, hepatitis B, and influenza has increased quickly in the past 20 years. Patent publications for RNA therapeutics for hepatitis C have decreased in recent years, most likely due to the approval of several effective small molecule drugs for hepatitis C. RNA therapeutics for atherosclerosis, hypertension, glaucoma, and age-related macular degeneration research have remained relatively stable. The top 20 patent assignees for patent publications on RNA therapeutics, vaccines, or diagnostics are mostly in the U.S. or China, although a few are in Germany, Korea, Japan, Switzerland, or Israel (Figure 10).

### Pipeline Dynamics of RNAs for Therapeutics and Vaccines

**Figure 11:**
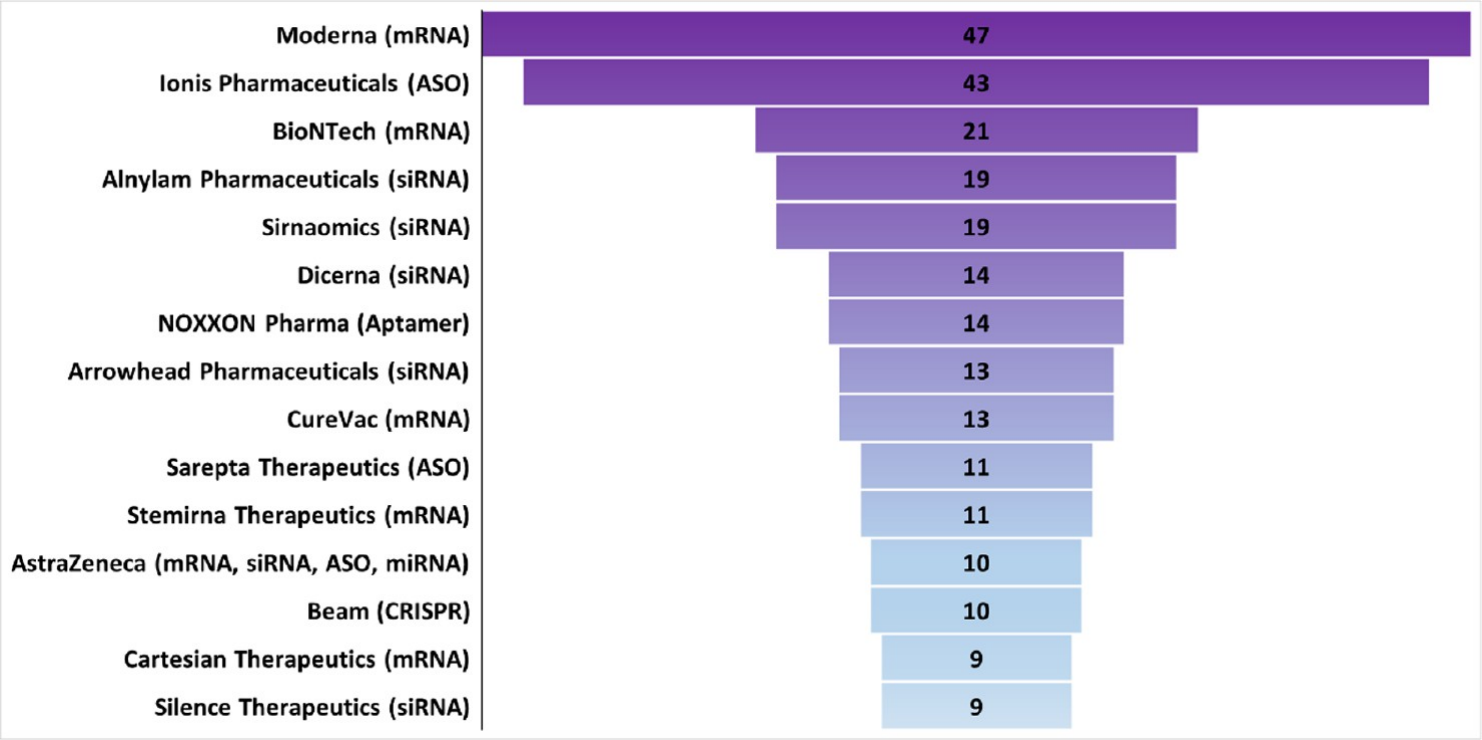
Top pharmaceutical companies ranked by the number of RNA therapeutic and vaccine agents in the development pipeline. Counts include RNA agents in company-announced pre-clinical development, in clinical trials, or approved. A single RNA agent can be counted multiple times when applied to multiple diseases.

After decades of extensive research, the therapeutic potential of RNAs has led to the development of over 250 therapeutics that are approved or in development (Table S2). Among the top 15 RNA therapeutics companies (Figure 11), which are located worldwide (Figure S2), each mostly focuses on one type of RNA to develop novel RNA therapeutics for treating diseases that range from very rare to common. mRNA and siRNA are the most common RNAs used by the top 15 companies, followed by ASO, CRISPR, and aptamers. Many of these companies are among the top patent assignees for RNA therapeutics, vaccines, and diagnostics in the commercial sector (Figure 10).

Moderna, BioNTech, CureVac, Stemirna Therapeutics, and Cartesian Therapeutics specialize in mRNA related therapeutics or vaccines. Moderna, headquartered in Massachusetts, USA,^122^ leads this group with over 45 therapeutics in its pipeline (Figure 11) and 131 RNA therapeutic/diagnostic patents (Figure 10). BioNTech and CureVac are both headquartered in Germany.^123,124^ BioNTech has in its pipeline 21 therapeutics that utilize the immune system to treat cancer and infectious diseases (Figure 11).^123^ CureVac has 13 therapeutics (Figure 11) along with 76 RNA therapeutic/diagnostic patents (Figure 10) for mRNA medicines.^124^ Stemirna Therapeutics headquartered in China,^125^ has a pipeline of 11 mRNA-based therapeutics (Figure 11). Cartesian Therapeutics, headquartered in Maryland, USA,^126^ has 9 therapeutics and is a pioneer in using mRNA for cell therapies within and beyond oncology, with products in development for autoimmune and respiratory disorders (Figure 11).

Alnylam, Sirnaomics, Arrowhead Pharmaceuticals, Silence Therapeutics, and Dicerna develop siRNAs for their RNA therapeutics. (Effective December 28, 2021, Dicerna is a wholly owned subsidiary of Novo Nordisk. In this paper Dicerna is considered separately.) Alnylam Pharmaceuticals and Dicerna are headquartered in Massachusetts, USA.^127,128^ Alnylam is the leader in siRNA therapy with over 19 therapeutics in their pipeline and 77 therapeutic/diagnostic patents (Figure 11). Dicerna treats both rare and common diseases with its 14 therapeutics (Figure 11). Sirnaomics, headquartered in Maryland, USA, has developed over 19 therapies for human diseases that have no treatments.^129^ Arrowhead Pharmaceuticals, headquartered in California, USA,^130^ treats previously intractable diseases with their 13 therapeutics (Figure 11). Silence Therapeutics, headquartered in London, UK,^131^ has 9 therapeutics (Figure 11).

Ionis Pharmaceuticals and Sarepta Therapeutics use ASOs for their RNA therapeutics. Ionis Pharmaceuticals is headquartered in California, USA,^132^ and Sarepta Therapeutics in Massachusetts, USA.^133^ Ionis has developed over 40 therapeutics (Figure 11) and 115 therapeutic/diagnostic patents (Figure 10). Sarepta uses antisense technology to target neurological diseases, including neuromuscular and neurodegenerative disease, with their 11 therapeutics (Figure 11).

**Figure 12:**
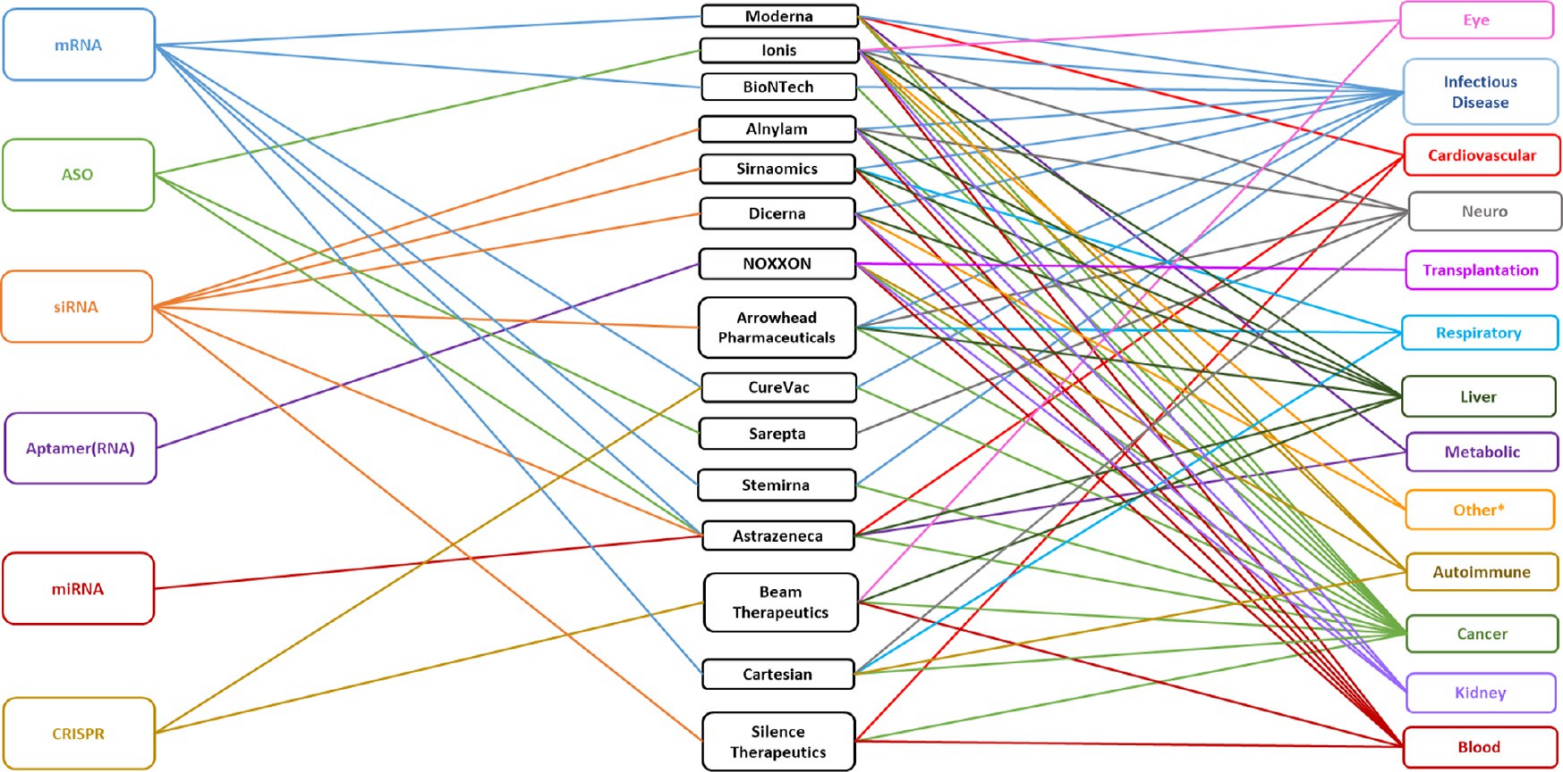
Types of RNA by company and targeted diseases. *Other: acromegaly, hereditary angioedema, and alcohol use disorder.

NOXXON Pharma, headquartered in Germany, uses RNA aptamers for their 14 therapeutics (Figure 11).^134^ Their current pipeline is focused only on oncology, but previously they researched treatments for transplantation, metabolic, blood, autoimmune, and kidney diseases (Figure 12). Beam Therapeutics, headquartered in Massachusetts, USA,^135^ is pioneering the use of base editing with CRISPR-Cas. Beam has 10 therapeutics in development (Figure 11). AstraZeneca supports multiple RNA platforms through partnerships with many of the top RNA companies.^136^

All but two of the top 15 RNA medicine companies specialize in one type of RNA (Figure 12). AstraZeneca supports multiple RNA platforms and CureVac is partnering with CRISPR Therapeutics for their current pre-clinical CRISPR RNA therapy^137^ along with their mRNA therapy. Companies typically specialize in one type of RNA but treat multiple diseases. All but two of the top 15 RNA medicine companies cover multiple diseases. Sarepta specializes in neurological and neuromuscular diseases and NOXXON has supported multiple diseases in the past (Figure 12) but is now dedicated exclusively to the treatment of cancer.

**Figure 13:**
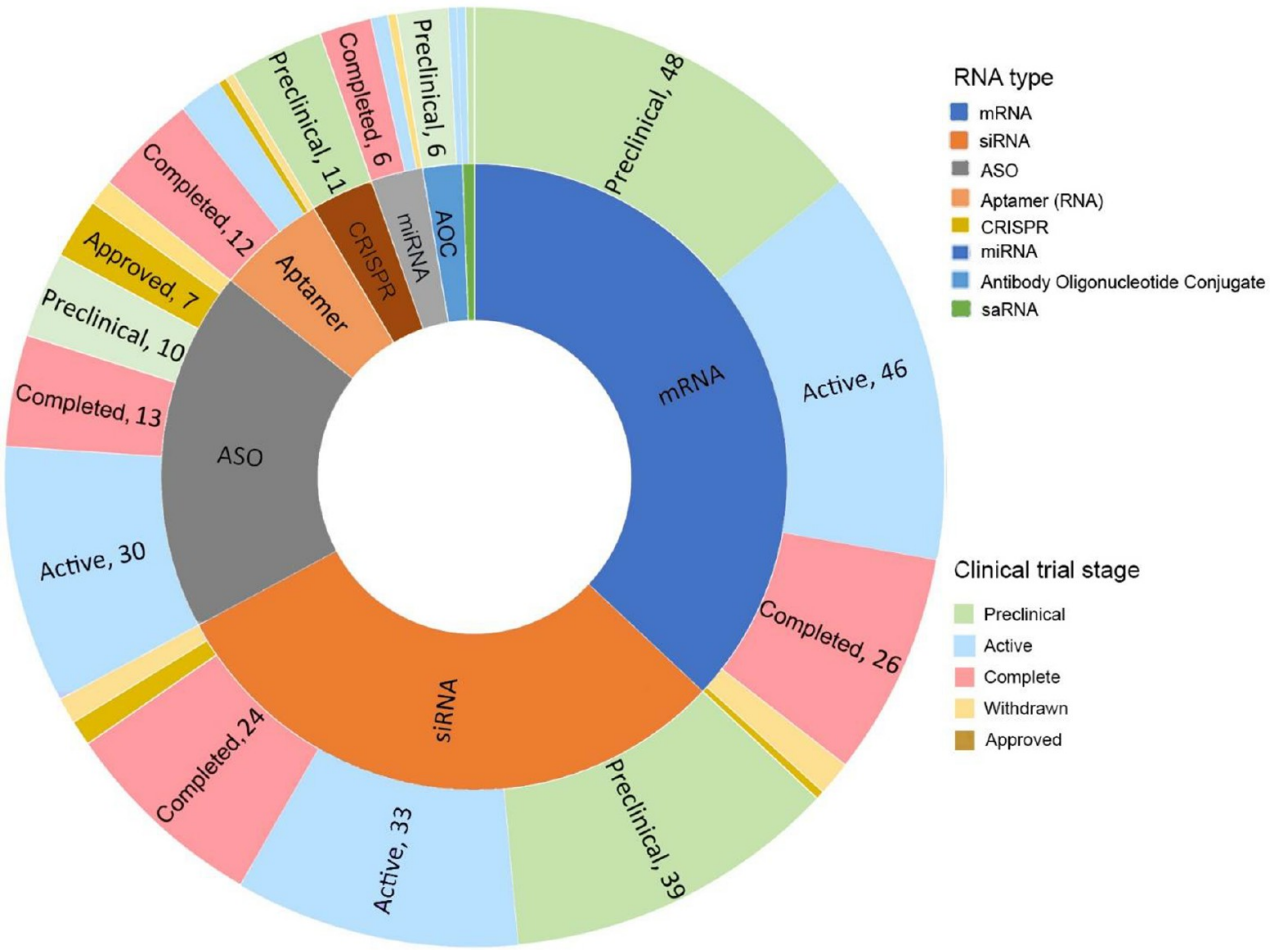
Counts of potential therapeutics and vaccines in different stages of development (pre-clinical, clinical, completed, withdrawn, and approved) for the various types of RNA. A full list of collected clinical trials is provided in Table S2.

**Figure 14:**
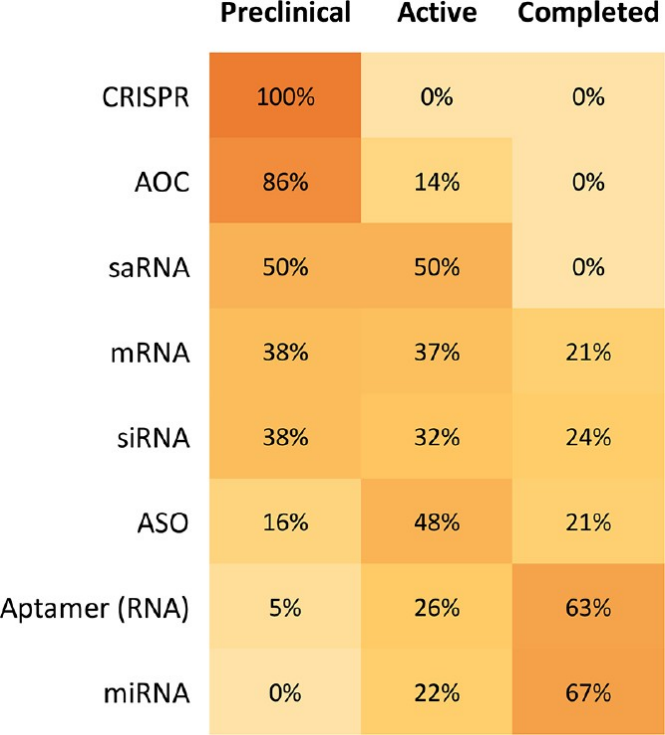
Percentage of pre-clinical, active, and completed clinical trials by RNA type. Figure rows may not sum to 100% because approved and withdrawn clinical trials are not included in this figure.

To investigate the research trends among different types of RNA therapeutics, the collected RNA therapeutics were further grouped by their types and development stages (Figures 13 and 14). The newer RNA therapeutics, such as AOCs and CRISPR, often have higher numbers of pre-clinical trials, indicating a great potential for future drug approval. In contrast, the more established types of RNA therapeutics, such as ASO and siRNA, often have a higher percentage of therapeutics on the market and a higher percentage of active and completed clinical trials, suggesting a shifting of research focus on the early development pipeline.

### Disease-Specific RNA Therapeutics and Vaccines

**Figure 15:**
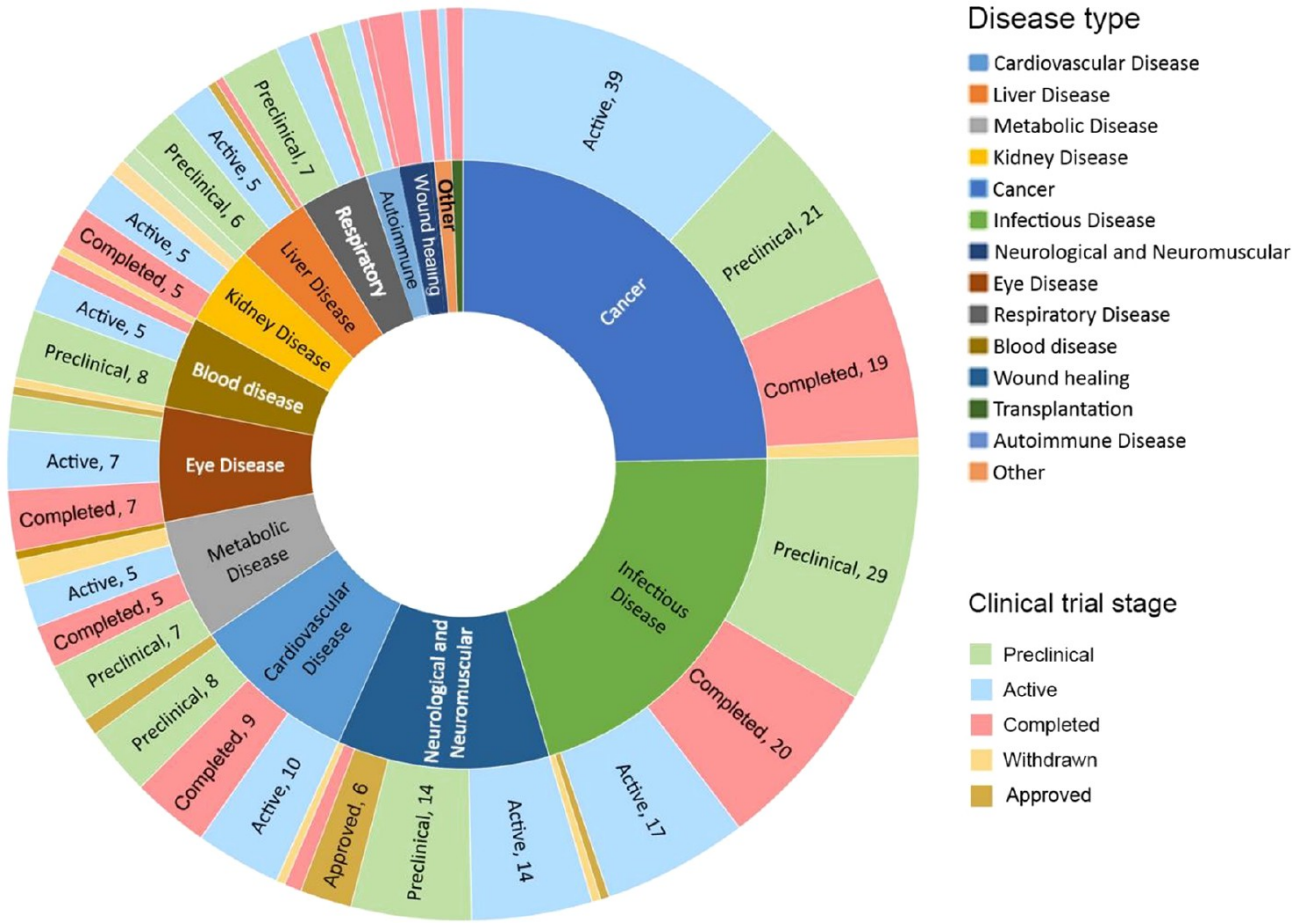
Counts of potential therapeutics and vaccines in different development stages (pre-clinical, clinical, completed, withdrawn, and approved) for various disease types. A full list of collected clinical trials is provided in Table S2.

**Figure 16:**
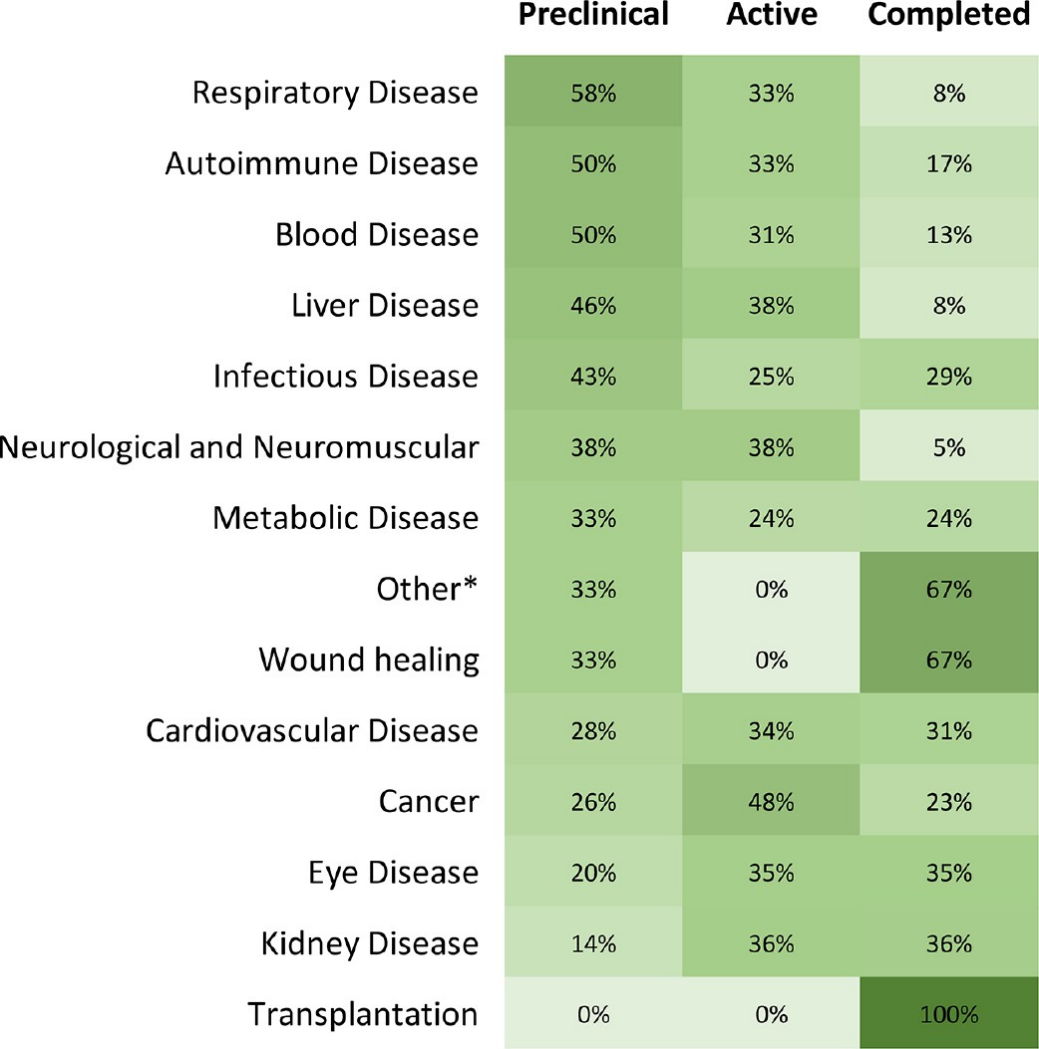
Percentage of pre-clinical, active, and completed clinical trials by disease type. Figure rows may not sum to 100% because approved and withdrawn clinical trials are not included in this figure. *Other: acromegaly, alcohol use disorder, and hereditary angioedema.

To further assess the pipeline dynamics, the above collected RNA therapeutics (Table S2) were then categorized based on their targeting diseases and development status (Figure 15). Cancer has attracted the highest number of therapeutics and vaccines in the research phase, with infectious diseases in second place. Neurological and neuromuscular diseases have the most approved treatments on the market, followed by cardiovascular and infectious diseases. The COVID-19 pandemic quickly catapulted RNA therapeutics for infectious diseases in both the research phase and approved vaccines to the forefront. While diseases such as familial hypercholesterolemia and DMD have approved RNA therapeutics, blood diseases, cancers, and respiratory diseases currently do not have any approved RNA treatments. Respiratory disease, autoimmune disease, and blood diseases have the highest percentage of therapeutics in pre-clinical trials but so far have no approved treatments. Figure 16 shows the percentages of RNA therapeutics in various development stages based on disease type, revealing places with high activities, as well as places needing more attention.

Disease types along with their RNA therapeutics are examined and summarized below (Tables 1–11). These tables and corresponding text are not exhaustive and highlight promising therapeutics in the development stages from the collected RNA therapeutics (Table S2).

**Cardiovascular diseases**, which account for 32% of all deaths, are the leading cause of death worldwide, taking an estimated 17.9 million lives each year.^138^ Cardiovascular diseases include disorders of the heart and blood vessels. Over 80% of cardiovascular disease deaths are due to heart attacks and strokes, and one-third of these deaths occur prematurely in people under 70 years of age.^138^

Leqvio is an approved therapeutic for the treatment of the cardiovascular disease, familial hypercholesterolemia (Table 1). Leqvio requires only two doses per year, and it received US FDA approval very recently in December 2021.^139^ It is a trivalent *N*-acetylgalactosamine (GalNAc)-conjugated siRNA with three GalNAc molecules clustered and conjugated to one siRNA molecule.^140^

**Table 1:** RNA Therapies for Cardiovascular Diseases

cardiovascular disease	drug name/lab code	type of RNA	target	company	development stage	clinical trial number
familial hypercholesterolemia	Leqvio	siRNA	proprotein convertase subtilisin/kexin type 9	Alnylam/Novartis	FDA approval in 2021^139^	
cardiovascular disease with high LpA	SLN360	siRNA	lipoprotein A	Silence Therapeutics	phase I	NCT04606602^141^
hypertension	Zilebesiran	siRNA	angiotensinogen	Alnylam	phase I	NCT03934307^147^
ischemic heart disease	AZD8601	mRNA	vascular endothelial growth factor-A	Moderna/AstraZeneca	phase II	NCT03370887^148^

In addition to this approved one, there are several more promising RNA therapeutics for cardiovascular diseases currently in the clinical trial stages (Table 1). Silence Therapeutics is evaluating the siRNA product SLN360 in a phase I clinical trial for its safety, tolerance, and pharmacodynamic and pharmacokinetic response in individuals with high lipoprotein A (LpA) cardiovascular disease.^141^ By targeting the LPA gene, SLN360 lowers the levels of LpA, decreasing the risk of heart disease, heart attacks, and strokes.^131^ Alnylam is also evaluating the siRNA product Zilebesiran that targets angiotensinogen for sustained reduction of hypertension.^142^ Interim phase I results show >90% reduction in serum angiotensinogen (AGT) for 12 weeks at a dosage of 100 mg or greater given quarterly or biannually.^143^ This dosing regimen and the continued efficacy and safety of the drug are being evaluated in a phase II study initiated in June 2021 (NCT04936035).^143^ Zilebesiran also uses Alnylam’s trivalent GalNAc-conjugated siRNA delivery platform.^140^ AstraZeneca and Moderna are collaborating on the mRNA drug AZD8601 to treat ischemic heart disease.^144^ AZD8601 targets vascular endothelial growth factor-A (VEGF-A).^145^ When AZD8601 is injected into the epicardium, VEGF-A is produced close to the damaged heart muscle, allowing cardiac regeneration.^146^

**Metabolic diseases** affect over a billion people worldwide by causing too much or too little of essential substances in the body.^149^ Diabetes alone affects 422 million people worldwide, causing 1.5 million deaths annually.^150^ IONIS-GCGR_Rx_ is an ASO product developed by Ionis Pharmaceuticals that treats diabetes by reducing the production of the glucagon receptor (GCGR) (Table 2).^151^ Glucagon is a hormone that opposes the action of insulin and stimulates the liver to produce glucose, particularly in patients with type-2 diabetes.^152^ Waylivra, another ASO product by Ionis Pharmaceuticals targeting apolipoprotein C-III (apoC-III), received European Union (EU) approval in 2019 as a treatment for familial chylomicronemia syndrome (FCS) (Table 2).^153^ FCS is a disease that prevents the body from breaking down consumed triglycerides. ApoC-III protein, which is produced in the liver, regulates plasma triglyceride levels in FCS patients, and Waylivra (volanesorsen) reduces its mean plasma levels.^154^

**Table 2:** RNA Therapies for Metabolic Diseases

metabolic disease	drug name/lab code	type of RNA	target	company	development stage	clinical trial number
diabetes	IONIS-GCGRRx	ASO	glucagon receptor GCGR	Ionis Pharmaceuticals	phase II	NCT01885260^161^
familial chylomicronemia syndrome	Waylivra	ASO	apolipoprotein C-III	Ionis Pharmaceuticals	EU approval in 2019^153^	
alpha-1 antitrypsin deficiency	ARO-AAT	siRNA	mutant of α1-antitrypsin	Arrowhead Pharmaceuticals	phase II	NCT03946449^162^
alpha-1 antitrypsin deficiency	unnamed	CRISPR	precise correction of E342K mutation	Beam Therapeutics	pre-clinical^157^	
methylmalonic acidemia	mRNA-3705	mRNA	mitochondrial enzyme methylmalonic-CoA mutase	Moderna	phase I/II	NCT04899310^163^

Alpha-1 antitrypsin deficiency (AATD) is a hereditary metabolic disease. Alpha-1 antitrypsin (AAT) is a glycoprotein produced in the liver that travels through the bloodstream to protect the lungs from inflammation.^155^ Mutations in the *SERPINA1* gene cause a deficiency of AAT in the blood, leading to toxic effects in the lungs and the accumulation of high levels of AAT in the liver that cause liver damage.^155^ ARO-ATT, a siRNA product developed by Arrowhead Pharmaceutical, targets the *SERPINA1* mutation, and in pre-clinical studies has shown promise in reducing AAT liver disease (Table 2).^156^ Beam Therapeutics is developing a CRISPR base editing drug to treat AATD by correcting the E342 K mutation in the *SERPINA1* gene.^157^ Beam’s base editor has two main components, a CRISPR protein bound to a guide RNA and a base editing enzyme, which are fused to form a single protein.^158^ This fusion allows the precise targeting and editing of a single base pair of DNA, which has not been previously achieved.^158^ Repairing the mutation would restore normal gene function and eliminate abnormal AAT production. This editing system uses a non-viral lipid nanoparticle delivery system.^157^

Methylmalonic acidemia (MMA) is a hereditary metabolic disease in which the body is unable to metabolize certain proteins and lipids correctly.^159^ MMA is caused by mutations in the *MMUT*, *MMAA*, *MMAB*, *MMADHC*, and *MCEE* genes. The mutation in the *MMUT* gene accounts for about 60% of MMA cases. Moderna has developed an mRNA therapeutic mRNA-3705 that targets the *MMUT* mutation (Table 2).^144^ mRNA-3705 instructs the cell to restore the missing or dysfunctional proteins that cause MMA. mRNA-3705 entered clinical trials with the first patient treated in August 2021.^160^

**Liver diseases** affect more than 1.5 billion people worldwide^164^ and account for over 2 million deaths per year.^165^ The siRNA therapeutic GIVLAARI, developed by Alnylam, received FDA approval in 2019 for the treatment of acute hepatic porphyria (Table 3).^142^ Acute hepatic porphyria is a genetic disease characterized by life-threatening acute attacks and chronic pain.^166^ GIVLAARI is the first approved GalNAc-conjugated RNA therapeutic.^167^

**Table 3:** RNA Therapies for Liver Diseases

liver disease	drug name/lab code	type of RNA	target	company	development stage	clinical trial number
acute hepatic porphyria	GIVLAARI	siRNA	5-aminolevulinic acid synthase 1 mRNA	Alnylam	FDA approval in 2019^170^	
non-alcoholic fatty liver disease	ION839/AZD2693	ASO	patatin-like phospholipase domain-containing protein 3	Ionis/AstraZeneca	phase I	NCT04483947^168^

Non-alcoholic steatohepatitis (NASH) is an accumulation of fat in the liver that causes liver damage. The antisense therapeutic ION839/AZD2693 from Ionis/AstraZeneca entered phase I trials (Table 3) in 2020 in patients with NASH and fibrosis.^168^ ION839/AZD2693 targets patatin-like phospholipase domain-containing 3 (*PNPLA3*), reducing its expression.^151^ Mutation of *PNPLA3*, which produces a protein that accumulates on the surface of intracellular lipid droplets, is strongly associated with an increased risk for NASH.^169^

**Cancer** includes a large group of diseases that are characterized by abnormal cell growth in various parts of the body. It is the second leading cause of death globally, accounting for an estimated 10 million deaths per year with over 19 million new cases diagnosed in 2020.^171^ Currently there are no approved RNA therapeutics for cancer treatment. NOXXON Pharma’s lead aptamer candidate NOX-A12 is in development as a combination therapy for multiple types of cancers (Table 4).^172^ It is intended to enhance other anti-cancer treatments without side effects. A phase I/II trial of NOX-A12 in combination with radiotherapy in newly diagnosed brain cancer patients who would not benefit from standard chemotherapy is ongoing.^172^ Interim data from the study in June 2021 showed tumor reduction in five of six patients, consistent with an anti-cancer immune response, and there were no unexpected adverse events.^172^ NOXXON is collaborating with Merck in their pancreatic cancer program.^172^ NOXXON, in a phase I/II combination trial with Merck’s Keytruda, reported success in treating metastatic pancreatic and colorectal cancer and entered a second collaboration with Merck to conduct a phase II study in pancreatic cancer patients.^173^ NOX-A12 targets C-X-C motif chemokine ligand 12 (CXCL12),^172^ which, with its receptors, acts as a link between tumor cells and their environment, promotes tumor proliferation, new blood vessel formation, and metastases, and inhibits cell death.^174^

**Table 4:** RNA Therapies for Cancers

cancer	drug name/lab code	type of RNA	target	company	development stage	clinical trial number
brain cancer/glioblastoma	NOX-A12	aptamer (RNA)	C-X-C motif chemokine ligand 12	NOXXON Pharma	phase I/II	NCT04121455^179^
pancreatic cancer	NOX-A12	aptamer (RNA)	C-X-C motif chemokine ligand 12	NOXXON Pharma	phase II	NCT04901741^180^
solid tumor	STP707	siRNA	transforming growth factor beta, cyclooxygenase-2	Sirnaomics	phase I	NCT05037149^181^
multiple myeloma	Descartes-11	mRNA	B-cell maturation antigen	Cartesian Therapeutics	phase I/II	NCT03994705^182^

The anti-cancer siRNA product, STP707 by Sirnaomics,^175^ targets TGF-β1 and COX-2 mRNAs (Table 4). A pre-clinical study demonstrated that knocking down *TGF-β1* and *COX-2* gene expression simultaneously in the tumor microenvironment increases active T cell infiltration and combining the two siRNAs produces a synergistic effect that diminishes pro-inflammatory factors.^176^ Descartes-11 developed by Cartesian is a CAR T-cell therapy for treating multiple myeloma, a white blood cell cancer that affects plasma cells (Table 4).^177^ It recently completed (March 2022) phase II studies with newly diagnosed patients.^178^ Descartes-11 contains autologous CD8^+^ T cells engineered with RNA chimeric antigen receptors (CARs) that bind to B-cell maturation antigen (BCMA).^177^ BCMA is highly expressed in all myeloma cells, and Descartes-11 binds and destroys BCMA-positive myeloma cells.^177^

**Infectious diseases** are caused by bacteria, viruses, fungi, or parasites.

SARS-CoV-2 has infected over 549 million people, causing over 6.1 million deaths worldwide.^183^ The COVID-19 pandemic brought the first approved mRNA vaccine to market. BioNTech/Pfizer’s COVID-19 BNT162/Comirnaty vaccine was given U.S. FDA emergency use authorization in 2020 and approved by the FDA in 2021 (Table 5).^184^ Moderna’s COVID-19 mRNA-1273/Spikevax vaccine was also given US FDA emergency use authorization in 2020 and approved by the FDA in 2022 (Table 5).^185^ Globally over 11.39 billion COVID-19 vaccine doses have been administered.^186^

**Table 5:** RNA Therapies and Vaccines for Infectious Diseases

infectious disease	drug name/lab code	type of RNA	target	company	development stage	year (first posted)	clinical trial number
COVID-19	BNT162/Comirnaty	mRNA	SARS-CoV-2 spike protein	BioNTech/Pfizer	FDA approval in 2021^184^		
COVID-19	mRNA-1273/Spikevax	mRNA	SARS-CoV-2 spike protein	Moderna	FDA approval in 2022^185^		
hepatitis B viral	AB-729	siRNA	hepatitis B viral surface antigen	Arbutus Biopharma Corporation	phase I	2021	NCT04775797^192^
influenza	mRNA-1010	mRNA		Moderna	phase I/II	2021	NCT04956575^193^
respiratory syncytial virus	unnamed	mRNA		CureVac	pre-clinical^137^		
respiratory syncytial virus	mRNA-1345	mRNA		Moderna	phase I	2021	NCT04528719^194^

Arbutus Biopharma Corporation developed AB-729, an RNA interference (RNAi) therapeutic specifically designed to reduce all hepatitis B virus (HBV) antigens, including hepatitis B surface antigen (HBsAg) (Table 5).^187^ HBsAg interferes with host immune response,^188^ and preliminary data indicate that long-term suppression of HBsAg with AB-729 results in an increased HBV-specific immune response.^187^

Moderna’s first quadrivalent seasonal influenza mRNA vaccine candidate mRNA-1010 is in phase I/II trials (Table 5).^144^ mRNA-1010 targets influenza lineages recommended by the World Health Organization (WHO) for the prevention of influenza, including seasonal influenza A H1N1 and H3N2 and influenza B Yamagata and Victoria.^189^ CureVac has a mRNA prophylactic vaccine for respiratory syncytial virus (RSV), a common respiratory virus that can cause serious illness in infants and older adults, in their pipeline under pre-clinical development (Table 5).^137^ Moderna has also developed a vaccine for RSV.^144^ Moderna’s mRNA-1345 (Table 5) is currently in a phase I trial to evaluate its tolerance and reactogenicity in children, younger adults, and older adults.^190,191^

**Neuromuscular diseases** affect the function of muscles and nerves that communicate sensory information to the brain.^195^ They affect the brain as well as the nerves found throughout the body and the spinal cord.^196^ These types of diseases have the greatest number of approved RNA therapeutics. Approved treatments for Duchenne muscular dystrophy include Sarepta’s ASO therapeutics Exondys 51, which targets exon 51 of the dystrophin gene, receiving FDA approval in 2016,^197^ Vyondys 53, which targets exon 53 of the dystrophin gene, receiving FDA approval in 2019,^198^ and NS Pharma’s Viltepso, also targeting exon 53 of the dystrophin gene, receiving FDA approval in 2020 (Table 6).^199^ Ionis Pharmaceuticals received FDA approval in 2016 for ASO therapeutic Spinraza targeting survival of motor neuron 2 (SMN2) for the treatment of spinal muscular atrophy (Table 6).^200^ Their second therapeutic approved by the FDA in 2018 was the ASO neurological therapeutic Tegsedi, which targets hepatic production of transthyretin (TTR) and is used for the treatment of hATTR amyloidosis-polyneuropathy, a disease due to mutations in the gene encoding TTR that leads to abnormal amyloid deposits on nerves.^201^ Onpattro by Alnylam is another approved RNA drug for the treatment of hATTR amyloidosis-polyneuropathy by targeting hepatic production of transthyretin (Table 6).^202^ Onpattro, which was FDA approved in 2018 (Table 6), was the first siRNA drug.^26^ Sarepta Therapeutics has two other ASO therapeutics for the treatment of DMD, SRP-5044 and SRP-5050 (targeting exon 44 and exon 50 of the dystrophin gene, respectively), that are in pre-clinical studies (Table 6).^203^

**Table 6:** RNA Therapies for Neuromuscular and Neurological Diseases

neurological and neuromuscular disease	drug name/lab code	type of RNA	target	company	development stage	clinical trial number
Duchenne muscular dystrophy	Exondys 51	ASO	exon 51 dystrophin	Sarepta Therapeutics	FDA approval in 2016^197^	
Duchenne muscular dystrophy	Vyondys 53	ASO	exon 53 dystrophin	Sarepta Therapeutics	FDA approval in 2019^198^	
Duchenne muscular dystrophy	Viltepso	ASO	exon 53 dystrophin	NS Pharma	FDA approval in 2020^199^	
spinal muscular atrophy	Spinraza	ASO	survival of motor neuron 2 mRNA	Ionis Pharmaceuticals	FDA approval in 2016^200^	
hATTR amyloidosis-polyneuropathy	Tegsedi	ASO	transthyretin mRNA	Ionis Pharmaceuticals	FDA approval in 2018^201^	
hATTR amyloidosis-polyneuropathy	Onpattro	siRNA	transthyretin mRNA	Alnylam	FDA approval in 2018^207^	
amyotrophic lateral sclerosis	Tofersen	ASO	superoxide dismutase 1	Ionis Pharmaceuticals/Biogen	phase III	NCT04856982^208^
Duchenne muscular dystrophy	SRP-5044	ASO	exon 44 dystrophin	Sarepta Therapeutics	pre-clinical^203^	
Duchenne muscular dystrophy	SRP-5050	ASO	exon 50 dystrophin	Sarepta Therapeutics	pre-clinical^203^	
myotonic dystrophy type 1	AOC 1001	antibody–oligonucleotide conjugate	myotonic dystrophy protein kinase	Avidity Biosciences	phase I/II	NCT05027269^209^
Duchenne muscular dystrophy	AOC 1044	antibody–oligonucleotide conjugate	exon 44 dystrophin	Avidity Biosciences	pre-clinical^205^	

Amyotrophic lateral sclerosis (ALS) is a progressive neurodegenerative disease that affects nerve cells in the brain and spinal cord.^204^ Ionis Pharmaceuticals in partnership with Biogen has an ASO investigational drug, Tofersen, in phase III clinical trials (Table 6).^151^ Tofersen targets superoxide dismutase 1 (SOD1), the second most common and best understood genetic cause of ALS.

Avidity Biosciences has a pipeline of antibody oligo-nucleotide conjugate (AOC) therapeutics focused on neuromuscular diseases. Their leading candidate, AOC 1001, is a siRNA conjugated with a monoclonal antibody (mAb) (Table 6).^205^ AOC 1001 has an ongoing phase I/II trial in adults with myotonic dystrophy type 1 (DM1). DM1 is a progressive neuromuscular disease that impacts skeletal and cardiac muscle. DM1 is caused by an abnormal number of CUG triplet repeats in the myotonic dystrophy protein kinase gene (DMPK), reducing muscleblind-like protein (MBNL) activity and disrupting muscle development.^206^ AOC 1001 is designed to reduce DMPK levels and CUG triplet repeats so that MBNL can perform normally.^205^ Avidity Biosciences has also developed an AOC to treat DMD.^205^ The oligonucleotides are designed to promote skipping of specific exons to produce dystrophin in patients with DMD. Their leading DMD drug candidate, AOC 1044, can induce exon skipping specifically for exon 44 (Table 6), and clinical trials are planned for 2022.^205^

**Eye diseases** including visual impairment affect 2.2 billion people globally.^210^ Macular degeneration, which causes loss in the center of the field of vision and is irreversible, affects 196 million people.^211^ Macugen, developed by Gilead Sciences (Table 7), was the first FDA-approved (2014) RNA aptamer treatment for neovascular age-related macular degeneration.^212^ Macugen targets the Vascular endothelial growth factor (VEGF) protein in the eye, reducing the growth of blood vessels to control the leakage and swelling that cause vision loss.^213^ As age-related macular degeneration (AMD) progresses, geographic atrophy (GA), a chronic progressive degeneration of the macula, can develop.^214^ Zimura by Iver Bio is another RNA aptamer therapeutic and is currently in a phase III clinical trial (Table 7). Zimura inhibits complement component 5,^215^ which is involved in the development and progression of AMD. In clinical trials, Zimura slowed the progression of GA over 12 months in individuals with age-related macular degeneration.^214^ QR-504a from ProQR Therapeutics is an investigational RNA ASO therapeutic that is in ongoing phase I/II clinical trials.^216^ QR-504a is designed to slow vision loss in individuals with Fuchs endothelial corneal dystrophy (FECD) which results from trinucleotide repeat expansion mutations in the Transcription factor 4 (*TCF4*) gene.^217^

**Table 7:** RNA Therapies for Eye Diseases

eye disease	drug name/lab code	type of RNA	target	company	development stage	clinical trial number
macular degeneration	Macugen	aptamer (RNA)	vascular endothelial growth factor protein	Gilead Sciences	FDA approval in 2014^212^	
geographic atrophy related to age-related macular degeneration	Zimura	aptamer (RNA)	complement component 5	IVERIC Bio	phase III	NCT04435366^215^
Fuchs endothelial corneal dystrophy	QR-504a	ASO	transcription factor 4	ProQR Therapeutics	phase I/II	NCT05052554^216^

**Kidney disease** affects over 850 million people worldwide.^218^ Primary hyperoxaluria type 1 (PH1) is a genetic kidney disease characterized by the overproduction of oxalate, which leads to kidney stones, kidney failure, and systemic oxalosis.^218^ The siRNA therapeutic Oxlumo was developed by Alynlam and approved by the FDA in 2020 (Table 8).^219^ Oxlumo targets the hydroxy acid oxidase 1 (*HAO1*) gene, which encodes glycolate oxidase.^219^ Oxlumo reduced urinary oxalate excretion in patients with progressive kidney failure in PH1.^219,220^ The majority of patients had normal or near-normal levels of oxalate after 6 months of treatment. Autosomal dominant polycystic kidney disease (ADPKD) is caused by mutations in the *PKD1* or *PKD2* gene.^221^ It is characterized by cysts within the kidneys, often leading to kidney failure. To treat ADPKD, Regulus designed RGLS4326, a second-generation oligonucleotide that inhibits miR-17 (Table 8), which produces kidney cysts. RGLS4326 binds to miR-17 microRNAs, inhibits miR-17 activity, and reduces disease progression.^222^ Immunoglobulin A (IgA) nephropathy is a chronic kidney disease that is caused by deposits of protein IgA inside the glomeruli of the kidneys.^223^ Overproduction of complement factor B (FB) is associated with increased IgA nephropathy. Ionis has partnered with Roche to develop a ligand-conjugated ASO therapeutic, IONIS-FB-LRx, which targets FB to reduce the production of IgA and alleviate the symptoms of IgA nephropathy (Table 8).^151^

**Table 8:** RNA Therapies for Kidney Diseases

kidney disease	drug name/lab code	type of RNA	target	company	development stage	clinical trial number
primary hyperoxaluria type 1	Oxlumo	siRNA	hydroxy acid oxidase 1	Alnylam	FDA approval in 2020	NCT04152200^224^
autosomal dominant polycystic kidney disease	RGLS4326	oligonucleotide	miR-17	Regulus	phase I	NCT04536688^225^
IGA nephropathy	IONIS-FB-LRx	ASO	complement factor B	Ionis/Roche	phase II	NCT04014335^226^

**Respiratory diseases** affect more than 1 billion people worldwide.^227^ COVID-19 brought acute respiratory distress syndrome (ARDS) to the forefront of medical news during the pandemic. ARDS is a severe inflammatory lung disease with a mortality rate of over 40%.^228^ Inflammation leads to lung tissue injury and leakage of blood and plasma into air spaces, resulting in low oxygen levels and often requiring mechanical ventilation.^228^ Descartes-30 is an engineered mRNA cell therapy product by Cartesian Therapeutics (Table 9). It comprises human mesenchymal stem cells that secret two human DNases for degrading ARDS-causing neutrophil extracellular traps (NETS).^177^ Descartes-30 is currently in phase I/II clinical trials.^229^

**Table 9:** RNA Therapies for Respiratory Diseases

respiratory disease	drug name/lab code	type of RNA	target	company	development stage	clinical trial number
acute respiratory distress syndrome	Descartes-30	mRNA	neutrophil extracellular traps	Cartesian Therapeutics	phase I/II	NCT04524962^229^
cystic fibrosis	MRT5005	mRNA	cystic fibrosis transmembrane conductance regulator	Translate Bio	phase I/II	NCT03375047^236^
cystic fibrosis	ARO-ENaC	siRNA	epithelial sodium channel α subunit	Arrowhead Pharmaceuticals	phase I/IIa	NCT04375514^237^

Cystic fibrosis is caused by a dysfunctional cystic fibrosis transmembrane conductance regulator (CFTR) protein, resulting from mutations in the *CFTR* gene.^230^ Without CFTR, mucus in various organs including the lungs is extremely thick and sticky. MRT5005, developed by Translate Bio, is an mRNA therapeutic (Table 9) that bypasses this mutation by delivering mRNA encoding a fully functional CFTR protein to the cells in the lungs through nebulization.^231^ Although initial interim results from clinical studies showed promise,^232^ results from the second interim phase I/II clinical trial showed no increase in the lung function of individuals receiving MRT5005.^233^ The siRNA therapeutic ARO-ENaC is designed by Arrowhead Pharmaceuticals to reduce epithelial sodium channel alpha subunit (αENaC) in the lungs and airways (Table 9). Increased ENaC contributes to airway dehydration and increased mucus.^234^ However, Arrowhead paused a phase I/II study of ARO-ENaC in July 2021 after safety studies showed local lung inflammation in rats.^235^

**Blood diseases** affect one or more components of blood. Sickle cell disease is a type of blood disease that causes red blood cells to become misshapen and break down.^238^ Beta-thalassemia syndromes are a group of blood diseases that result in reduced levels of hemoglobin in red blood cells.^239^ BEAM-101 (Table 10), Beam Therapeutics leading *ex vivo* base editor, is a patient-specific, autologous hematopoietic investigational cell therapy. BEAM-101 introduces base edits that mimic the single nucleotide polymorphisms found in individuals with hereditary persistence of fetal hemoglobin (HPFH), which could alleviate the effects of mutations causing sickle cell disease or beta-thalassemia since the fetal hemoglobin does not become misshapen.^240^ Beam plans to initiate a phase I/II clinical trial to assess the safety and efficacy of BEAM-101 for the treatment of sickle cell disease.^240^ BEAM-101 uses an electroporation delivery system.^157^ Silence Therapeutics has RNA therapeutics for blood diseases such as thalassemia, myelodysplastic syndrome, and rare iron-loading anemias.^241^ Their siRNA therapeutic SLN124 targets transmembrane serine protease 6 (*TMPRSS6*) (Table 10), a gene that prevents the liver from producing hepcidin.^241^ Phase I clinical trial data showed that SLN124 improved red blood cell production and reduced anemia by increasing the levels of hepcidin.^241,242^

**Table 10:** RNA Therapies for Blood Disease

blood disease	drug name/lab code	type of RNA	target	company	development stage	clinical trial number
sickle cell disease/beta-thalassemia	BEAM-101	CRISPR	fetal hemoglobin activation	Beam Therapeutics	pre-clinical^157^	
thalassemia/low-risk myelodysplastic syndrome	SLN124	siRNA	transmembrane serine protease 6	Silence Therapeutics	phase I	NCT04718844^243^

**Keloid scarring** is a type of pathological condition that forms abnormally thick scarring after a skin injury. STP705, from Sirnaomics, is a siRNA therapeutic in phase II clinical trial for the treatment of keloid scars (Table 11).^244^ STP705 targets both *TGF-β1* and *COX-2* gene expression with polypeptide nanoparticle-enhanced delivery.^245^ The synergistic effect of simultaneous silencing *TGF-β1* and *COX-2* may reverse skin fibrotic scarring by decreasing inflammation and activating fibroblast apoptosis. This mechanism of action of STP705 can be widely applied for the treatment of other fibrotic conditions.^245^

**Table 11:** RNA Therapies for Other Diseases

other disease	drug name/lab code	type of RNA	target	company	development stage	clinical trial number
keloid scarring	STP705	siRNA	transforming growth factor beta 1/cyclooxygenase-2	Sirnaomics	phase II	NCT04844840^244^
autoimmune diseases	mRNA-6231	mRNA	interleukin-2	Moderna	phase I	NCT04916431^251^
alcohol use disorder	DCR-AUD	siRNA	aldehyde dehydrogenase 2	Dicerna Pharmaceuticals	phase I	NCT05021640^252^

**Autoimmune diseases** are characterized by immune activation in response to normal antigens.^246^ mRNA-6231 from Moderna is an mRNA encoding IL-2 mutein (Table 11) that activates and expands the regulatory T cell population and dampens self-reactive lymphocytes to help restore normal immune function.^247^

**Alcohol use disorder** (AUD) is the inability to control alcohol use despite adverse social, occupational, or health consequences. According to the World Health Organization, AUD affects over 283 million people globally.^248^ Alcohol is metabolized in the liver to acetaldehyde via alcohol dehydrogenase and then to acetic acid by aldehyde dehydrogenase 2 (ALDH2).^249^ Inhibiting ALDH2 results in unpleasant symptoms due to the increased acetaldehyde when alcohol is not fully metabolized.^250^ The siRNA therapeutic DCR-AUD by Dicerna knocks down ALDH2 protein expression (Table 11) in the liver, which results in increased acetaldehyde levels that discourage continued alcohol use in AUD patients.^250^

## Chemical Modifications for Improving RNA Stability and Target Specificity

**Figure 17:**
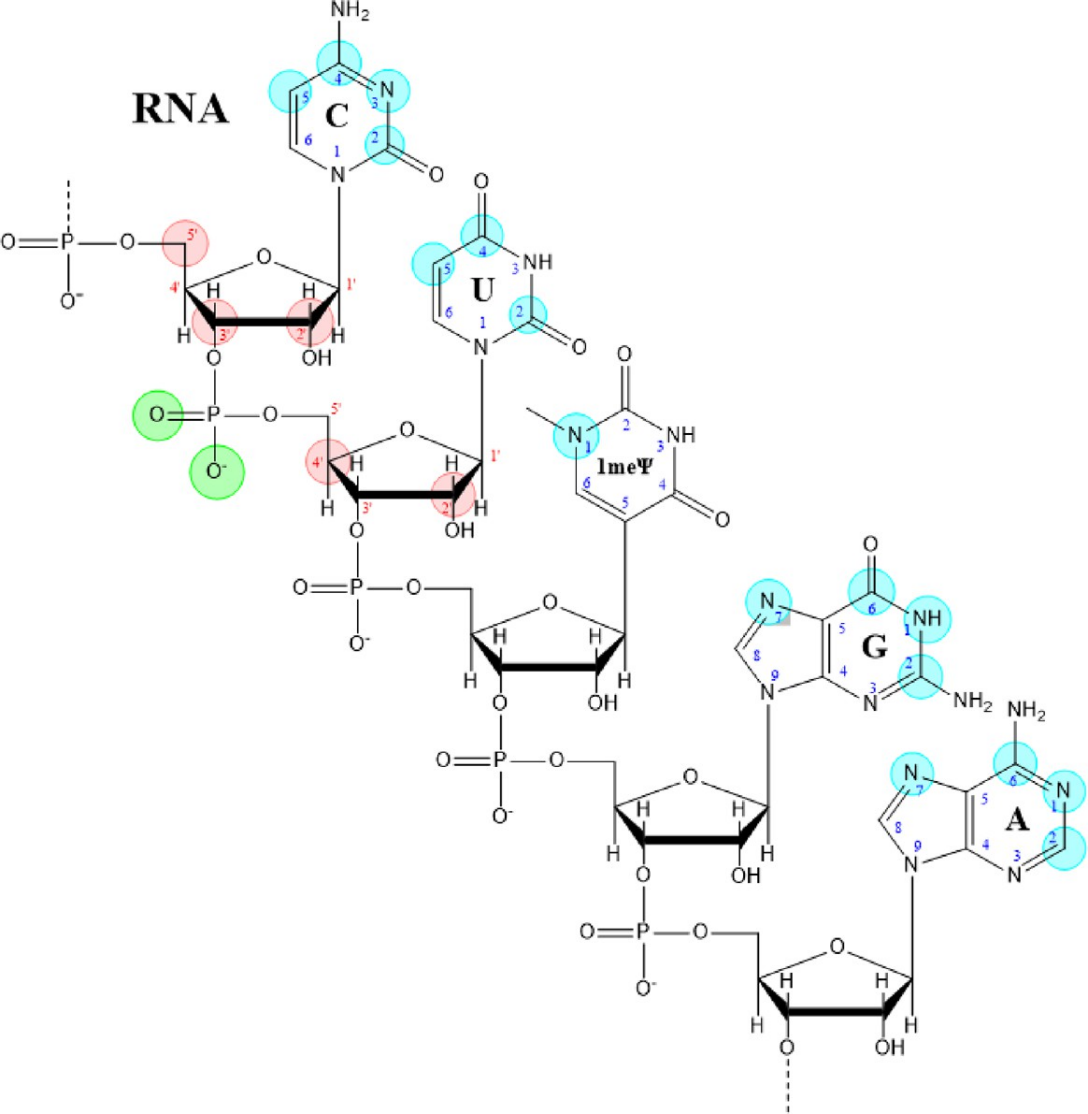
An example of RNA structure and modification sites. Green circles, modification sites on the phosphate; red circles, attachment sites for modifications on the ribose; blue circles, attachment sites for modifications on the nucleic acid base: 1-methylpseudouridine (1meΨ), cytosine (C), uridine (U), guanosine (G), and adenosine (A).

RNA is composed of nucleosides that consist of a nucleic acid base attached to a d-ribose through a β-*N*-glycosyl bond between the ribose and a pyrimidine base (uracil and cytosine) or a purine base (adenosine and guanosine). The nucleosides are connected by a phosphodiester bond using the phosphate between the 3′ and 5′ carbons on adjacent ribose molecules (Figure 17).^253^ RNAs can be modified on the nucleic acid base and on the phosphate and the ribose of the sugar–phosphate backbone, as illustrated in Figure 17 with color circles.

The nucleic acid side chains extending from the sugar–phosphate backbone form hydrogen bonds between the nucleic acid bases of complementary RNA chains; U bonds with A, and G bonds with C. Double-stranded RNA structures form as a result of intramolecular and intermolecular base pairing. Base pairing between loops of double-stranded stem-loops can yield 3D structures, and triple helixes can form within single strands or between multiple strands.

Chemical modifications on RNA can improve the stability and reduce the immunogenicity of therapeutic RNAs. RNA is very susceptible to nucleases and hydrolysis by basic compounds. Chemical modification of RNA protects the vulnerable sugar–phosphate backbone from nuclease degradation and lowers the risk of off-target effects. For RNAs that form a duplex with a target sequence, mutations that lower the melting temperature of the duplex destabilize the complex and improve target specificity by decreasing base pairing with non-target RNA. RNA modifications can also improve the delivery of the RNA into the cell through the plasma membrane and enhance the activity of the RNA.^104^

### Nucleic acid base modifications

**Figure 18:**
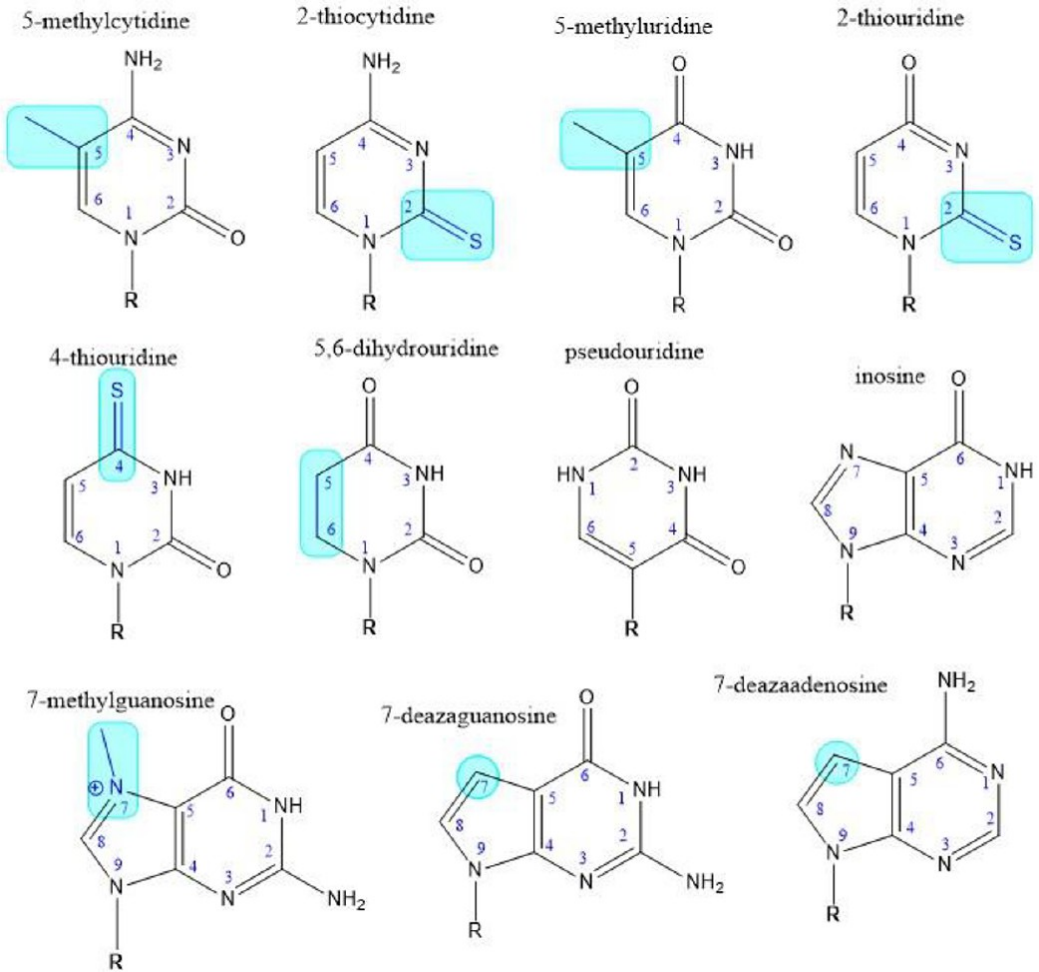
Examples of modified and rare bases. R = d-ribose; locations of modifications are shown in blue.

Figure 18 shows chemical structures of commonly seen base modifications and rare bases. Modifications include methylation, replacement of oxygen with sulfur, and replacement of a nitrogen within the ring system with carbon. Cytidines or uridines can be methylated at the N-5 position to be 5-methylcytidines or 5-methyluridines. Cytidines can have the oxygen replaced with a sulfur at the N-2 position to be 2-thiocytidines. When uridines have the oxygen replaced with a sulfur at either the N-2 or the N-4 position, they become 2-thiouridines or 4-thiouridines respectively. Uridines can also be reduced on the base ring and become 5,6-dihydrouridines. Another commonly seen base modification, methylation at the N-7 position of the guanosine, occurs in mRNA cap structure. The guanosines can also be modified at the N-7 position by converting the nitrogen to a carbon, and become 7-deazaguanosine. Similar modifications can happen to adenosines, resulting in 7-methyladenosines and 7-deazaadenosines. The rare bases, pseudouridine and inosine, are also seen in modified RNA sequences. Additional modified or rare bases are shown in Figure S7. According to the data in the CAS Content Collection,^32^ the use of RNA modifications has been increasing since 1995 (Figure S6).

Base modifications that interfere with the formation of hydrogen bonds can thermally destabilize duplex formation with the target, and thus improve target specificity by limiting off-target binding.^254^ In addition, modifications improve the performance of therapeutic RNA. Replacing uridine with the modified base 1-methylpseudouridine (Figure 17) in therapeutic mRNAs, such as the COVID-19 vaccines (Pfizer’s Comirnaty and Moderna’s Spikevac), improves translation and lowers cytotoxic side effects and immune responses to the mRNA.^255^ Both Pfizer’s Comirnaty and Moderna’s Spikevac mRNA vaccines also use a 7-methylguanosine cap linked by a 5′-triphosphate to the 5′ end of the mRNA, replicating the naturally occurring mRNA caps that prevent degradation of the 5′ end of the mRNA.^256^

### Modifications on Ribose

**Figure 19:**
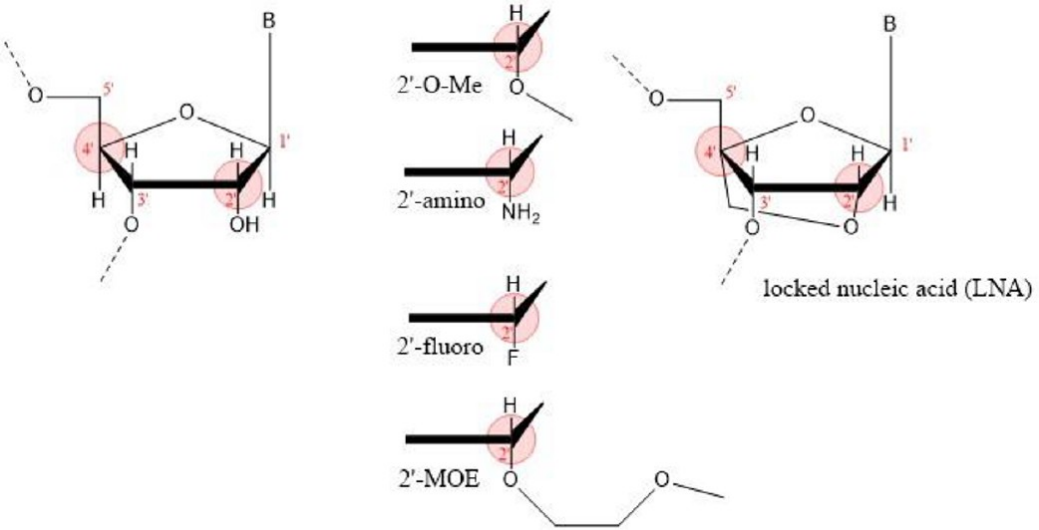
Common modifications on the 2′-hydroxyl group of d-ribose. B = nucleic acid base.

The hydroxyl group on the C-2′ position of the ribose destabilizes RNA compared to DNA. Modification of this hydroxyl protects against nuclease digestion and can lower the thermal stability of duplexes formed with a target RNA. Figure 19 shows the chemical structures of these modifications on ribose. The most common modifications at the C-2′ position include 2′-*O*-methyl, 2′-amine, 2′-fluoro, and 2′-*O*-methoxyethyl (2′-MOE). Another modification at the C-2′ position is the LNA in which a 2′-*O*,4′-*C*-methylene bridge connects the 2′ position to the 4′ position on the ribose.^257^

In addition, the 5′ and 3′ ends of RNA can be modified using cleavable linkers attached to GalNAc groups or lipophilic moieties to target the therapeutic RNA to the desired tissue. The linkers are cleaved by acid pH, redox potential, or degradative enzymes in cells but not in serum or blood. Cleavable linkers include acid-cleavable groups, ester-based cleavable groups, and peptide-based cleavable groups.^254^

### Backbone Modifications

Modifications to the phosphate group in the sugar–phosphate backbone can improve the resistance of therapeutic RNAs to extracellular and intracellular nucleases. In addition, the negative charge on the phosphate group interferes with the delivery of the RNA into the cell through the lipid bilayer membrane, which is impermeable to polar molecules. Thus, replacing the oxygens on the phosphates with neutral groups or complexing the phosphate groups to cations like sodium can improve RNA delivery.^258^

**Figure 20:**
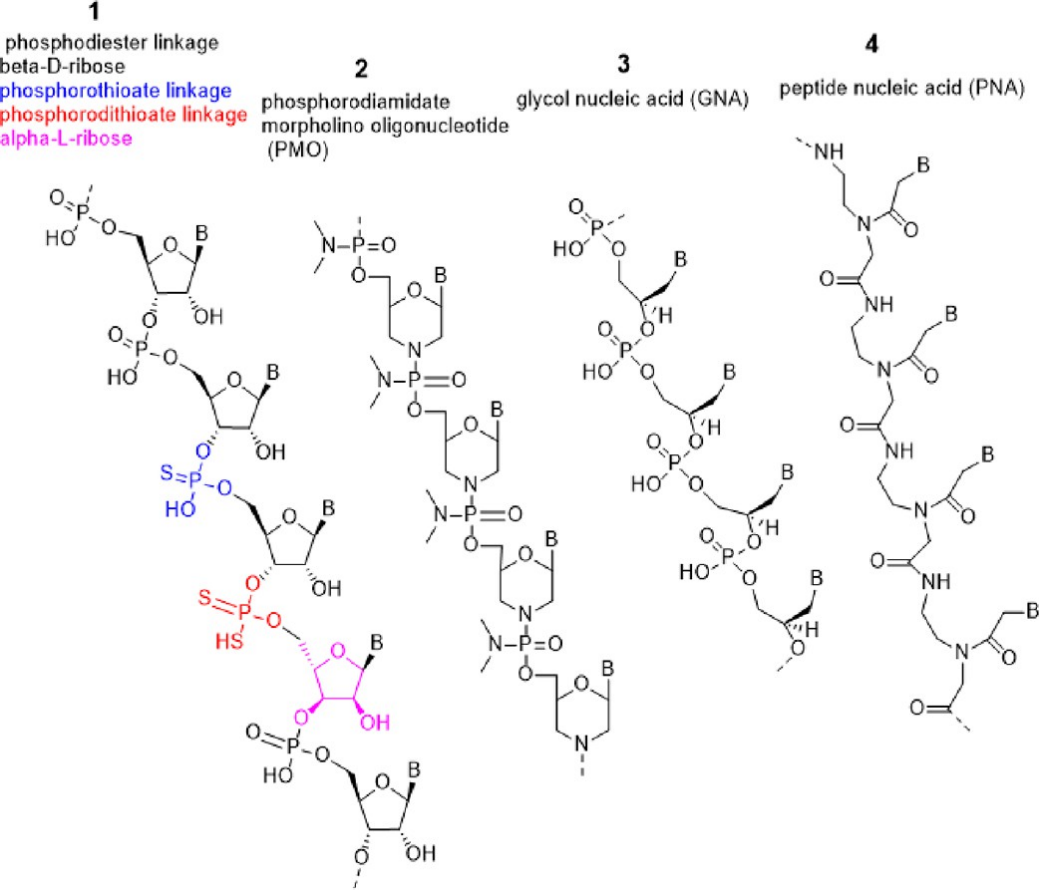
Examples of modified RNA backbones. Backbone 1 shows phosphate–ribose backbone linkages, which include the classic phosphodiester (black), phosphorothioate (blue), and phosphorodithioate (red). The purple ribose, α-l-ribose, has an alternative stereochemistry compared to the normal β-d-ribose moieties shown in black. Backbone 2 is a phosphorodiamidate morpholino (PMO) backbone, backbone 3 is (*R*)-glycol nucleic acid ((*R*)-GNA), and backbone 4 is peptide nucleic acid (PNA). B = nucleic acid base.

A widely used backbone modification, phosphorothioate, as shown in Figure 20, which replaces an oxygen in the phosphate group with sulfur, reduces the activity of extracellular and intracellular nucleases.^259^ RNA molecules with phosphorothioate linkages at the ends resist exonucleases, whereas RNA molecules with phosphorothioate linkages within the RNA resist endonucleases. However, the sulfur on the phosphate group creates stereogenic α-phosphorus atoms resulting in diastereomers with different functional properties that can affect duplex formation. Careful spacing of the phosphorothioate linkages within the RNA can ameliorate this problem.^257,258^

Other backbone modifications replace the d-ribose with either an l-ribose or a non-ribose moiety (Figure 20). Phosphorodiamidate morpholino oligonucleotides (PMOs) contain morpholino groups linked by phosphorodiamidate groups rather than ribose linked by phosphates. Glycol nucleic acids (GNAs) have a backbone of repeating glycerol units linked by phosphodiester bonds.^260^ In peptide nucleic acids (PNAs) the sugar–phosphate backbone is replaced with a flexible *N*-(2-aminoethyl)glycine polymer with the nucleobases attached via a methylene carbonyl linkage.^261^ PMOs, GNAs, and PNAs all resist nuclease degradation. PNA forms duplexes with complementary DNA or RNA with higher affinity and specificity than unmodified DNA–DNA or DNA–RNA duplexes. However, duplexes containing PNAs, PMOs, and LNAs resist RNase H degradation, inhibiting gene knockdown through targeted mRNA degradation.^257^

### Trends of RNA Chemical Modifications

**Figure 21:**
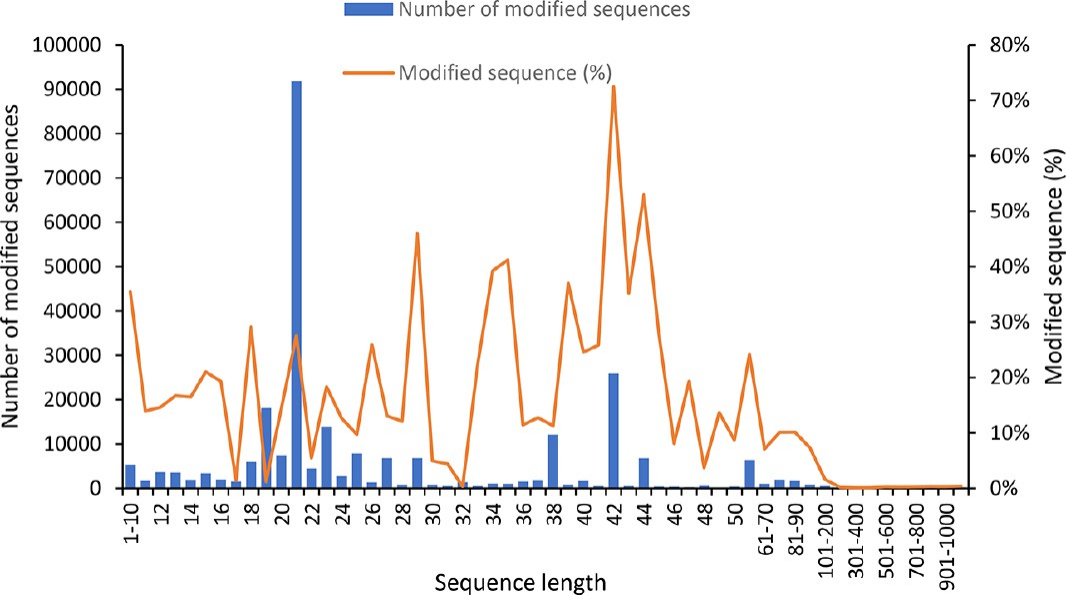
RNA sequences containing modifications and their distribution with respect to sequence lengths (from the CAS Content Collection). Blue bars: absolute number of modified RNA sequences; orange line: percentage of modified RNA sequences in the total RNA sequences with same sequence length.

Sequence data for RNAs and RNA modifications are annotated and collected with CAS Registry Sequence Guidelines^262^ from published documents and stored in the CAS Content Collection.^32^ To better understand the chemical modification trends on RNA molecules, we extracted ∼170,000 modified RNA sequences from the CAS Content Collection. Figure 21 shows the number of the modified RNA sequences and distribution of these modified sequences along the sequence length. The predominance of modified nucleotide RNAs for lengths of 18–27 bases reflects the fact that this sequence length is commonly used in siRNAs and ASOs; processed, naturally occurring double-stranded siRNAs are typically 21 or 23 bp long. The double-stranded nature of siRNAs accounts for the large number of modifications for nucleotides with a length of 42 and 44; two 21-nucleotide RNAs produce 42 nucleotides of RNA, while a 21-nucleotide and a 23-nucleotide RNA produce 44 nucleotides. The percentage of modified RNA sequences in the total RNA sequences was also shown along the sequence length, suggesting that sequences less than 100 base pairs are more frequently being modified than the longer sequences. This drop-off observed in RNA modification for RNAs over 100 nt is likely an artifact of the methods used to synthesize artificial RNA and the methods used to sequence naturally occurring RNAs. Small artificial RNAs are produced by solid-support synthesis or other chemical methods; larger RNAs are produced by in vitro transcription. Chemical synthesis has more capacity to add modified nucleotides to an RNA; in vitro transcription faces the limitations posed by the use of enzymes. Furthermore, naturally occurring RNA sequences are generally characterized by reverse transcription to cDNA, amplification, and then sequencing. This process would obscure modification present on the initial RNA.

**Figure 22:**
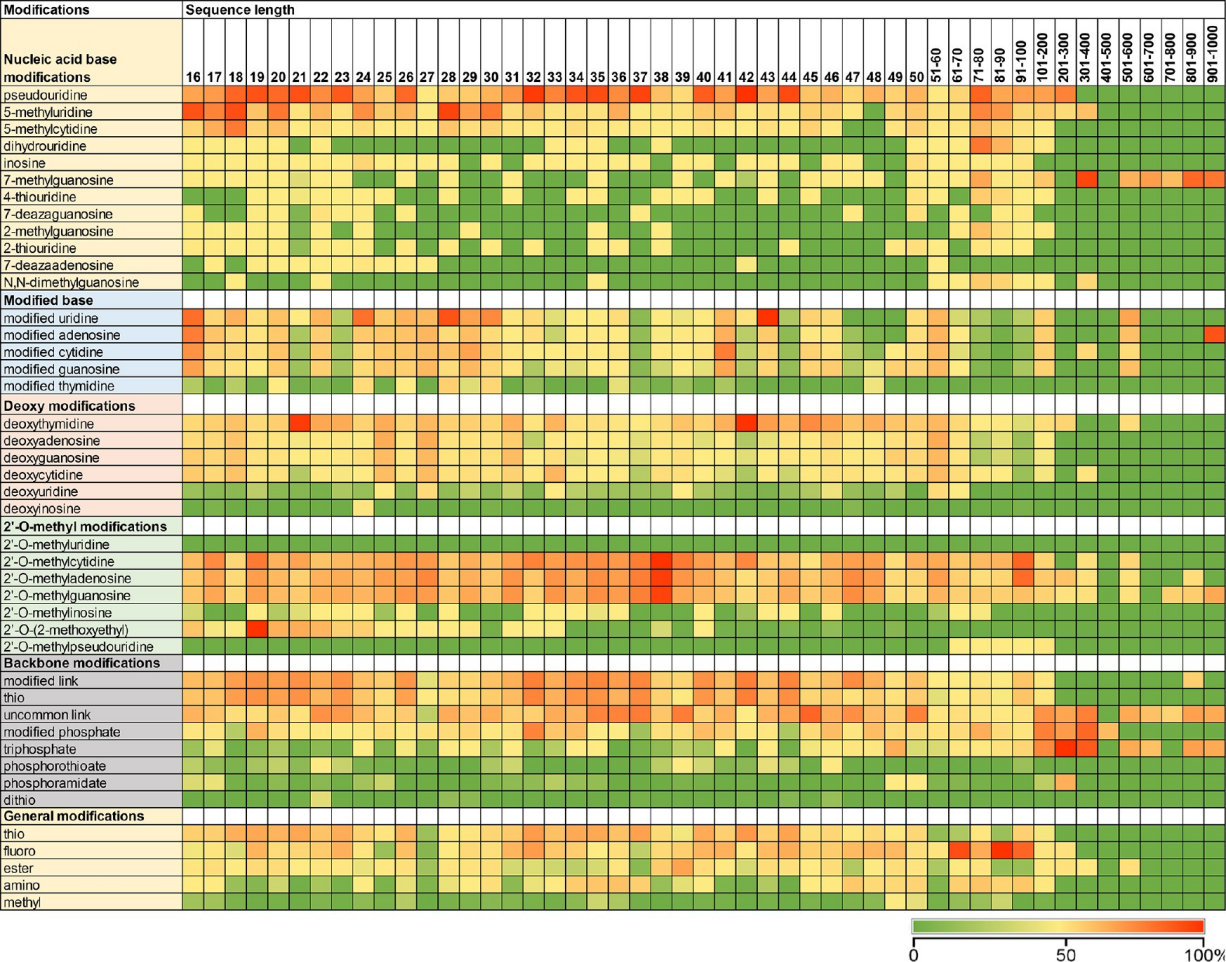
Frequencies of modifications of RNA and their distributions based on sequence lengths (CAS Content Collection).

Chemical modifications were further analyzed using this data set with specific types of modifications and sequence lengths. Out of 145 curated sequence modifications, 117 sequence modifications were identified in the modified RNA data set. A heatmap (Figure 22) covering the most popular modifications was constructed based on the relative frequencies of specific types of modification in the total modification events in that sequence length. The heat map of modification type versus sequence length shows a sharp change in the types of modifications in sequences <200 nucleotides vs >200 nucleotides. Sequences >200 nucleotides are modified much less than shorter sequences. This set of longer RNAs, which includes lncRNAs and mRNAs, is either transcribed in vivo or produced using *in vitro* transcription. The most common modifications contained in the longer sequences are triphosphates and 7-methylguanosines, suggesting that they are mRNAs with 5′ end caps consisting of 7-methylguanosine linked to the 5′ end of the mRNA with a triphosphate group. In addition, modified adenosine is observed in longer sequences, particularly in sequences from 901 to 1000 nucleotides; this would include the mRNA modification, 6-methyladenosine.^263,264^ Since therapeutic mRNAs are translated by the ribosomes to produce an active protein, excessive modification might provide steric hindrance that inhibits translation.

Common nucleobase modifications for small RNAs (those <200 nucleotides in length) are 5-methyluridine (m^5^U) and 5-methylcytosine (m^5^C). The rare base pseudouridine appeared frequently in the database, followed by the rare bases dihydrouracil and inosine. Bases in which the N-7 position is converted to a carbon (7-deazaguanine and 7-deazaadenine) appeared with some frequency, as did the replacement by sulfur of the oxygen double-bonded to the N-4 or N-2 position of uracil or the N-2 position of cytosine. Less common modifications included benzoyl groups attached to N-3 in cytosine and the nitrogens in adenosine.

Modifications at the C-2′ ribose position are also common, with 2′-O-methyl and 2′-deoxy modifications as the most highly represented and 2′-fluoro and 2′-MOE somewhat less. The prevalence of thymidine (deoxythymidine) at sequence lengths of 21 and 42 is because thymidine at the 3′ end of artificial siRNAs protects them from exonucleases. Artificial siRNAs are predominantly 21-mers, and since they are double-stranded, they are highly represented at both 21 and 42 nucleotides.

Phosphorothioate is the most common phosphate modification for the sugar–phosphate backbone, although phosphorodithioate also occurs. Altered ribose stereochemistry, such as l-ribose in place of d-ribose, is a relatively rare modification, as are replacement backbones, such as PMO, GNA, and PNA.

Heat maps for modifications versus disease targets show little correlation between the disease and the modifications on the therapeutic RNA (Figures S3 and S4). Overall, there is little correlation between the type of RNA and the targeted diseases (Figure S1), suggesting any RNA can be explored to target any disease.

**Figure 23:**
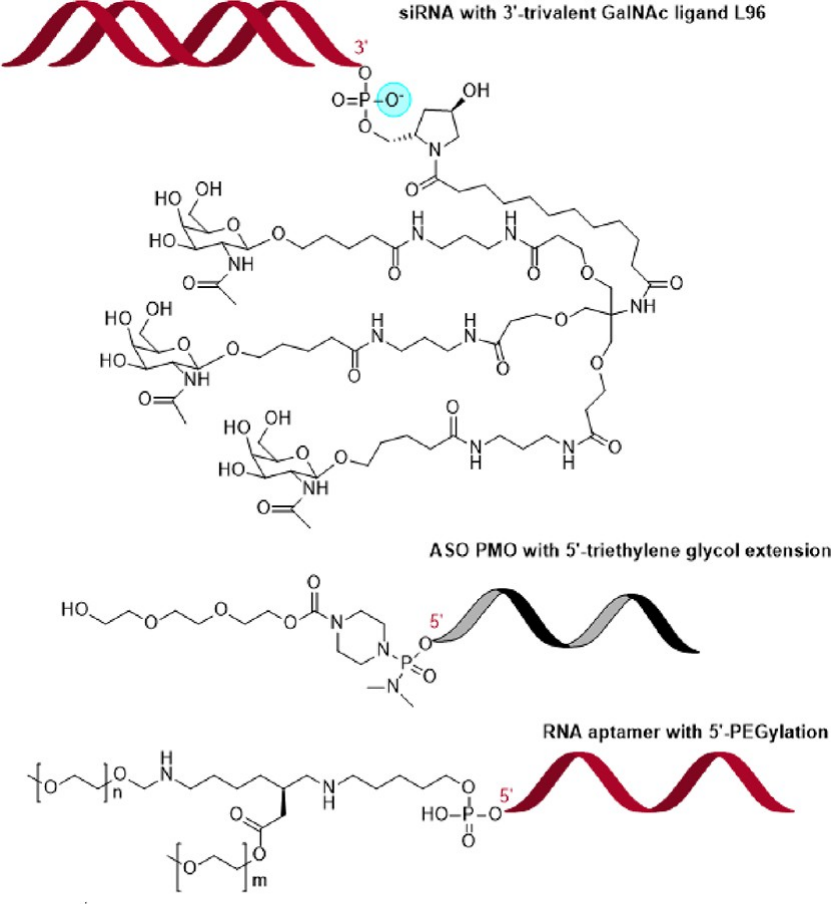
Examples of terminal modifications for therapeutic RNAs: siRNA with 3′ tripartite GalNAc ligand L96 (O in the blue circle can be replaced with S); ASO with a PMO backbone (black) and triethylene glycol; RNA aptamer with double 5′-PEGylation.^268−270^

Approved RNA medicines show a correlation between the type of RNA and specific modifications (Table 12). Of the approved RNA therapeutics, only ASOs have m^5^C and m^5^U. The ASOs have either a PMO backbone, a phosphorothioate backbone where all the nucleotides contain 2′-MOE groups, or a phosphorothioate backbone with blocks of nucleotides at the 5′ and 3′ ends containing 2′-MOE groups and internal 2′-deoxyribonucleotide residues. While the 2′-MOE groups protect the ASO from degradation, they also prevent RNase H digestion of the target mRNA. Thus, an ASO with an internal gap between the protected ends permits RNase H recognition of the target, which is the case for three of the four ASOs with 2′-MOE groups (Table 12).^257^ ASOs with PMO backbones (see Figure 21) regulate their targets through steric hindrance, not nuclease digestion.^265^ For the five ASOs in Table 12, three have phosphorothioate backbones and two, Exondys 51 and Vyondys 53, have PMO backbones together with a triethylene glycol extension at their 5′ end (Figure 23).

**Table 12:**
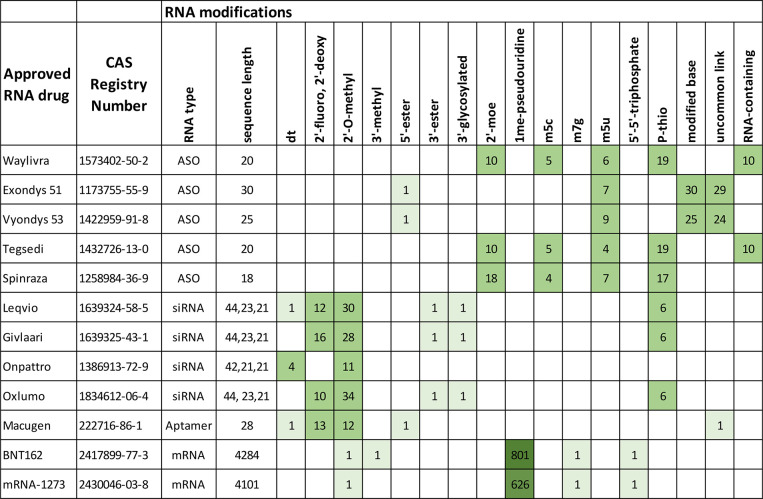
Number of RNA Modifications in Approved RNA Therapeutics^a^

a The color intensity reflects the number of RNA modifications.

siRNAs have a moderate number of phosphorothioate linkages, with 2′-fluoro and 2′-O-methyl modification of the ribose, and are often modified on their 3′ ends. Three of the approved therapeutic siRNAs, Leqvio, Givlaari/Girosiran, and Oxlumo, are 3′-glycosylated with the trivalent GalNAc conjugate^266^ shown in Figure 23. This trivalent branched linker containing GalNAc residues targets the siRNAs to hepatocytes to treat liver diseases.^267^ The other approved siRNA, Onpattro, has two 3′-terminal thymidine (dT) residues on both RNA strands to protect the siRNA from exonucleases.

Aptamers have ester linkages to protective groups and can have extensive sugar–phosphate backbone modifications, such as PEGylation (the covalent attachment of polyethylene glycol) on their 5′ ends. Because aptamers bind to their targets as a result of their tertiary structure and do not rely on nucleic acid hybridization for function, they have fewer constraints on modifications compared to other therapeutic RNAs.^104^ The single FDA-approved aptamer, Macugen, has extensive 2′-*O*-methyl and 2′-fluoro modifications, a 3′-to-3′ link to thymidine at its 3′ end, as well as double PEGylation at its 5′ end (Figure 23).

The mRNA vaccines BNT162/Comirnaty and mRNA-1273/Spikevax have 7-methylguanosine caps connected to the 5′ end of the mRNA by a triphosphate group and have all their uridines replaced with 1-methylpseudouridine, which improves mRNA stability and translation. In addition, both contain a 2′-O-methyl modification to the nucleotide immediately following the 7-methylguanosine cap to improve stability to base and nuclease digestion.

In summary, chemical modifications protect therapeutic RNAs from exonucleases, endonucleases, and the cellular environment, and they enhance pharmaceutical activity. The choice of the backbone determines whether an ASO blocks cellular processes such as translation, transcription, or splicing, or targets an RNA for nuclease digestion. The 2′-ribose modifications on siRNAs mitigate off-target effects by lowering the thermal stability, thereby enhancing target-specific binding. 1-methylpseudouridine improves stability and translation of therapeutic mRNAs. Since therapeutic RNAs are extensively modified, they frequently are not designated as RNA vs DNA but have names such as ASO (antisense oligonucleotide) and siNA (short interfering nucleic acid) for siRNAs.^271^

## RNA Delivery Systems

**Figure 24:**
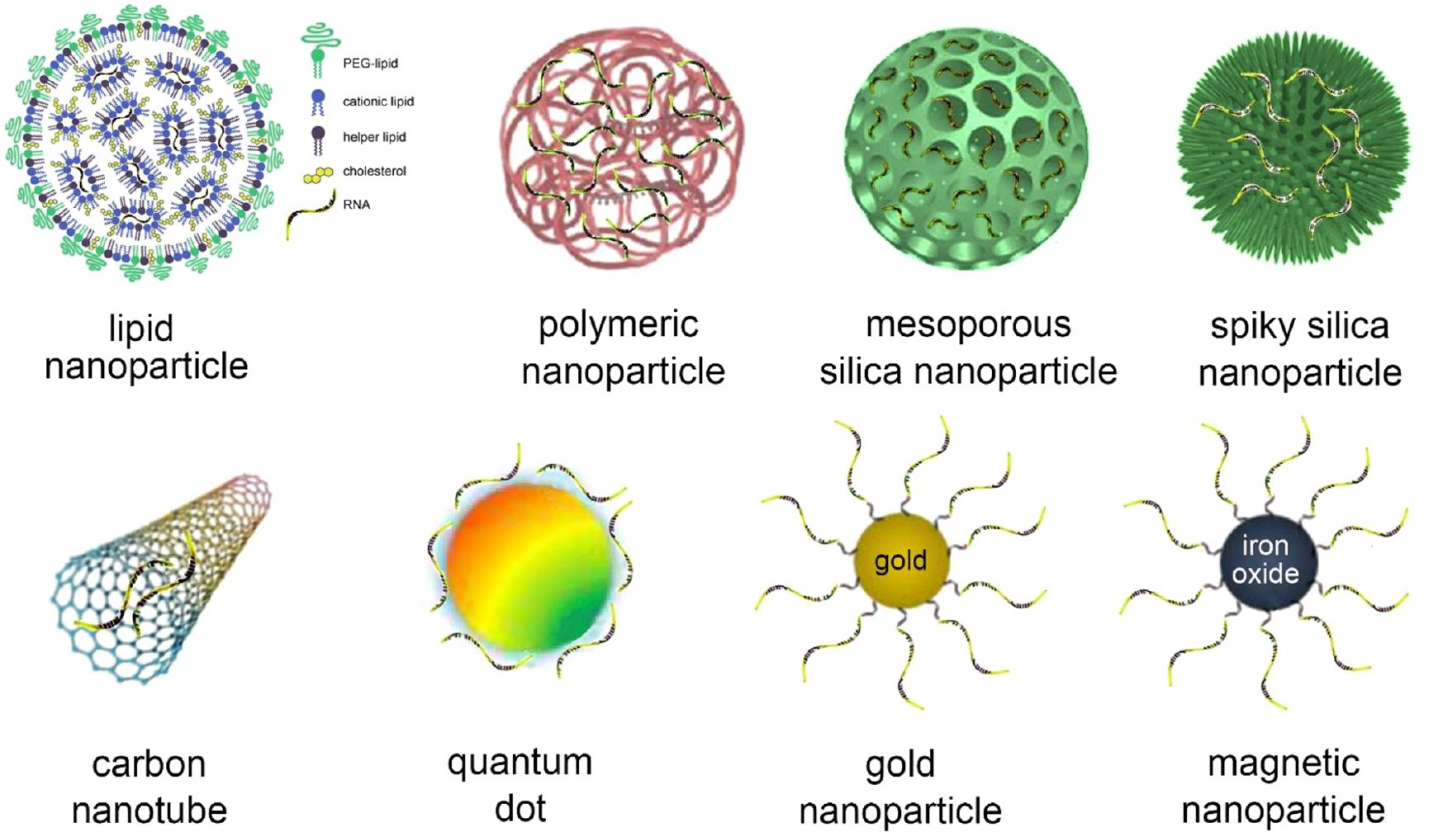
Examples of RNA nanocarriers.

RNA therapeutics, which are hydrophilic and negatively charged, cannot diffuse across cell membranes; thus, they require delivery vectors and/or chemical modification to reach their targets. When administered systemically, RNA delivery systems need to protect the RNA against serum nucleases, bypass the immune system, avoid non-specific interactions with serum proteins, and block renal clearance.^11^ While biological barriers such as immunogenicity and nucleases are usually addressed by modifying the RNA chemically, encapsulation of RNA into nanocarriers can both protect and deliver RNA to cells.^272^ Nanomaterials with biodegradability, biocompatibility, and low toxicity are used as RNA carriers. These include lipids, chitosan, cyclodextrin, polyethylenimine (PEI), poly(lactic-*co*-glycolic acid), dendrimers, magnetic nanoparticles, carbon nanotubes, gold nanoparticles, silica nanoparticles, and others (Figure 24).

### Research publications on RNA delivery systems

**Figure 25:**
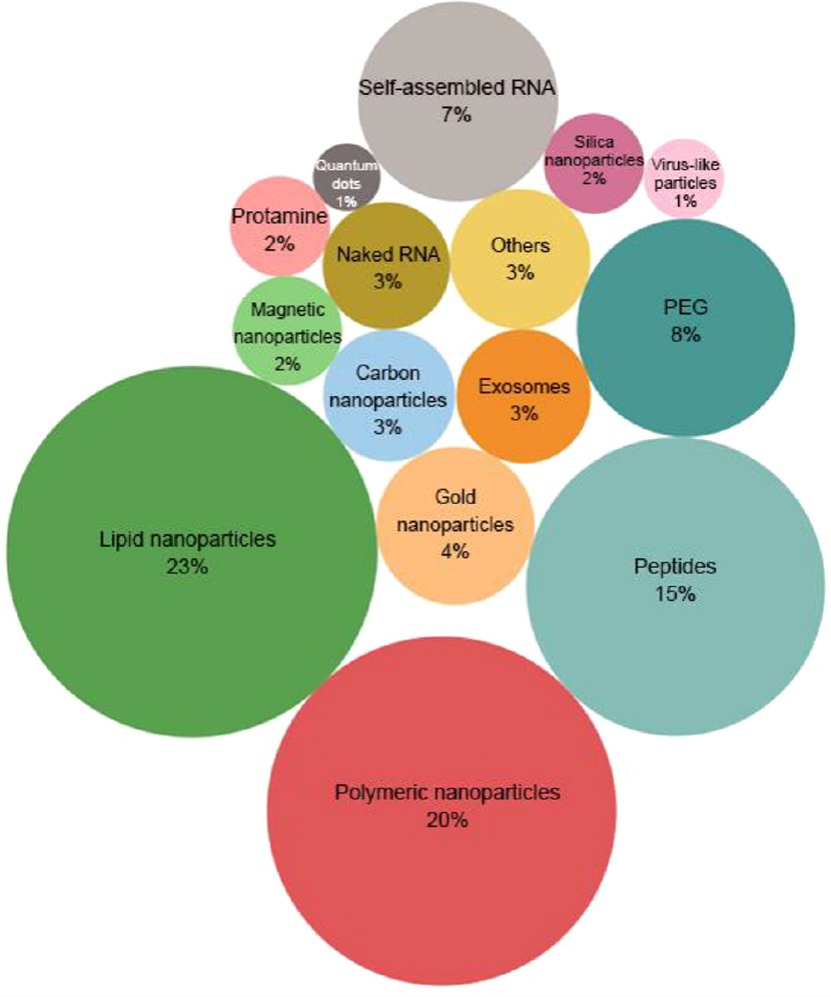
Percentage distribution of RNA nanocarrier-related documents in the CAS Content Collection.^32^

Nearly 7000 scientific publications on RNA delivery systems, including patents and non-patents (journal articles, books, dissertations, meeting abstracts, etc.), are in the CAS Content Collection.^32,273^ Publications of studies involving RNA carriers are dominated by lipid nanoparticles, followed closely by polymeric nanocarriers (Figure 25).

### Lipid Nanoparticles

Lipid nanoparticles, comprising stable complexes between synthetic cationic lipids and anionic nucleic acids, are currently the most widely used non-viral delivery system for nucleic acid drugs and vaccines.^19,274−276^ The advantages of lipid systems include ease of production, biodegradability, protection of entrapped nucleic acids from nuclease degradation and renal clearance, promotion of cellular uptake, and endosomal escape.^277^ Liposomes have been recognized for a long time for their role as immunological adjuvants in vaccines. As early as 1974 it was reported that they enhance the immune response to liposome-entrapped tetanus toxoid.^278^ Subsequently, this was shown to apply to many other antigens including mRNA vaccine products, such as the spike protein of the coronavirus.^279−281^

**Figure 26:**
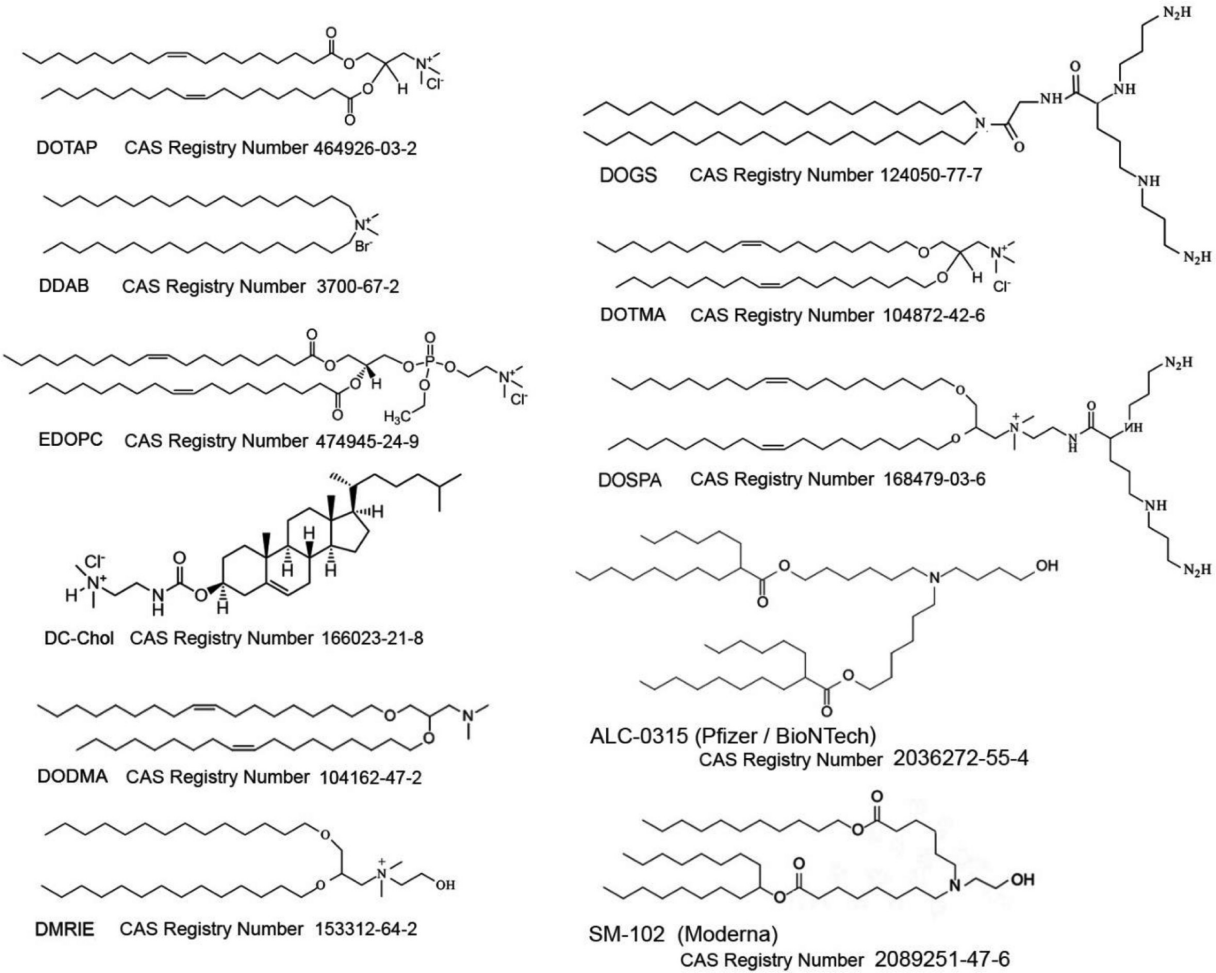
Examples of cationic lipids used as nucleic acid carriers. (An extensive list with the structures of the most frequently used cationic lipids in lipid nanoparticle pharmaceutical formulations according to the CAS Content Collection is available^276^).

Since the first cationic lipids successfully delivered plasmids into cells in 1987, many more have been synthesized and tested as nucleic acid carriers.^19^ Cationic lipids differ from natural lipids by having an ionizable (cationic) headgroup in place of the zwitterionic or anionic headgroup of the natural lipids. They include two hydrophobic alkyl chains or a cholesterol moiety, a positively charged polar headgroup, and a linker joining them. Ionizable lipids are positively charged inside the cell but are neutral in the bloodstream due to pH differences, and they are less toxic than non-ionizable cationic lipids.^282^ Examples of cationic lipid structures used as vectors for gene delivery are provided in Figure 26, and a comprehensive list, including the chemical structures of the ∼50 most frequently used cationic lipids in drug delivery,^32^ is available.^276^

The head groups of cationic lipids are typically amine derivatives such as primary, secondary, and tertiary amines (e.g., DOGS, DC-Chol), quaternary ammonium (e.g., DOTMA, DOTAP, DORIE, DMRIE), combinations of amines (e.g., DOSPA, GAP-DLRIE), and amidinium salts (e.g., diC14-amidine) (Figure 26). Guanidine and imidazole groups^283^ as well as pyridinium, piperazine, and amino acid headgroups such as lysine, arginine, ornithine, and tryptophan^284,285^ have also been used. Headgroups with multiple cationic charges such as DOSPA, DOGS^286^ may be more efficient than single-charged lipids^287^ since the multiple charges of the headgroups condense nucleic acids. However, multivalent cationic lipids bind strongly to the nucleic acid, preventing intracellular release, and they tend to form toxic micelles.^288^ Combinations of quaternary amines and polyamines enhance transfection efficiency. The first cationic lipid to combine these two functionalities, Lipofectamine, is the long-standing gold standard in nucleic acid delivery efficiency. It comprises DOSPA formulated with the helper lipid dioleoylphosphatidylethanolamine (DOPE).^289^

Recently, LNPs have been in the global spotlight as a vital component of COVID-mRNA vaccines, playing a key role as an immunological adjuvant, in effectively protecting and transporting mRNA to cells.^276,279,290^ These vaccines deliver mRNA encoding the SARS-CoV-2 spike protein into the cytoplasm of host cells, which is then translated and acts as an antigen for the development of an immune response. Both vaccines contain ionizable cationic lipids enabling RNA complexation. The hydrocarbon chains of the proprietary cationic lipids of both these vaccines are optimized to enable safe clearance and mRNA delivery efficiency. They also contain a PEGylated lipid to confer “invisibility” from antibody opsonization and phagocytosis. Packing of RNA into lipid nanoparticles is aided by distearoylphosphatidylcholine and cholesterol.

Complexation with cationic lipids stabilizes nucleic acids, protects them from nuclease degradation, and facilitates delivery to the target cells. Lipid nanoparticles carrying nucleic acids adsorb to the cell membrane, are endocytosed, and release the nucleic acids into the cell. Cell membranes have negative charges that attract the cationic lipid nanoparticles and drive the adsorption and fusion of the lipid nanoparticle with the cell membrane. The anionic lipids of the cells are thought to neutralize the charge of the cationic lipid carriers, thereby eliminating the electrostatic interactions between the lipid carriers and the nucleic acids, and facilitating the release of the nucleic acids. Neutralization of the cationic lipids also destroys the nanoparticles by promoting the formation of non-lamellar structures,^291^ which are associated with the efficacy of cationic lipid carriers in delivering nucleic acids into cells.^292^ Despite their benefits, lipid nanoparticles show cell toxicity and stimulate the release of systemic inflammatory cytokines, and lipid aggregates may accumulate in the liver and spleen causing hepatotoxicity.^276,293^

Nearly 20 years after scientists first prepared liposomes (the first-generation lipid nanoparticles), they found similar ∼100 nm extracellular lipid vesicles in most eukaryotic cells. They are exosomes that form naturally in the endosomes and are released from cells normally or as a result of some pathologies. They are formed by invaginations of the endosomal membrane and are subsequently released in the extracellular space via fusion with the plasma membrane.^294,295^ Although their functions are largely unknown, they are believed to be associated with important physiological processes such as the regulation of intercellular communications and signaling, and of transmission of macromolecules between cells.^296^ Exosomes, which are secreted by most cells, may be important in intercellular communication and signaling, and in the transport of proteins, lipids, and nucleic acids between cells. Since they are rich in mRNAs and small RNAs and can transport their contents to recipient cells, they are considered good candidates for RNA delivery. Exosome-mediated delivery of β-secretase-1 siRNA (targeting the enzyme β-secretase, which is important in Alzheimer’s disease) to the brain resulted in highly efficient β-secretase-1 gene knockdown in the mouse brain cortex.^297^ Electroporation has been applied to load exosomes with exogenous siRNA.^298,299^

Exosomes exhibit certain advantages over conventional delivery systems. They are natural transporters^300^ and hence less likely to be toxic or to cause immune responses. Moreover, exosomes can cross biological barriers such as the blood-brain barrier. The unique membrane composition of exosomes is key to their ability to enter target cells. The membranes of exosomes are enriched in cholesterol and phosphatidylserine.^301^ However, vesicles comprising only the lipids from the exosomal membrane are unable to fuse with cells, indicating that exosomal membrane proteins are also important for their activity.^301^ A notable advantage of the exosomes as compared to other nanoparticle delivery vehicles is that they do not lead to a harmful accumulation of therapeutic RNAs in the liver.^302,303^ In addition to exosomes, other biological carriers obtained from living organisms may include red blood cells, extracellular vesicles, platelet membrane-coating vehicles, red blood cell membrane coating nanoparticles, cancer cell membrane-coated nanoparticles, and macrophage-based vehicles.^304,305^ Red blood cells are appropriate as carriers because they lack both mitochondrial and nuclear DNA, so the risk of horizontal gene transfer is avoided.^306^ Macrophages are attractive carriers for solid tumor targeting RNA delivery due to their natural capacities to reside into tumors throughout tumor progression.^307^

### Polymeric Nanoparticles

Polymers comprise the second largest group of nucleic acid delivery vehicles after lipids. Cationic polymers form stable complexes (polyplexes) with anionic nucleic acids and offer a versatile, scalable, and easily adjustable platform for efficient nucleic acid delivery, while minimizing immune responses and cellular toxicity.^308^

**Figure 27:**
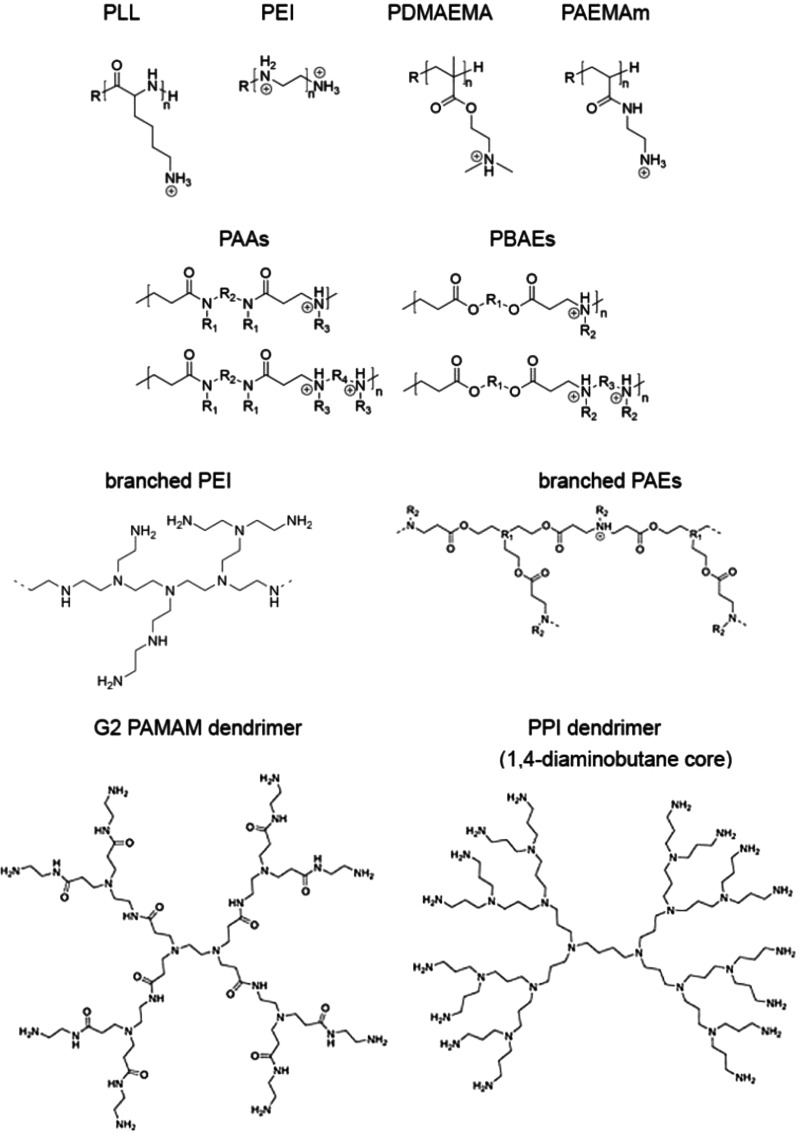
Examples of structures of polymeric nucleic acid carriers.

Linear cationic polymers are the most widely studied polymeric nucleic acid carriers.^309^ PEI, poly(l-lysine) (PLL), poly(2-(dimethylamino)ethyl methacrylate) (PDMAEMA), poly(2-aminoethyl methacrylamide) (PAEMA), poly(amidoamines) (PAAs), and poly(β-amino esters) (PBAEs) have been studied for drug delivery (Figure 27). These linear polymers differ in their cation types (primary amines such as PLL, secondary amines such as PEI, or tertiary amines such as PDMAEMA), and their cationic charge density.

Block linear copolymers comprising polycationic homopolymers and non-ionic hydrophilic blocks compact nucleic acids into polyplex micelles. PEG, hydrophilic acrylamide, acrylate, and methacrylate polymers can arrange the nucleic acid in a micelle core and create a hydrophilic protective shell. For example, the PEG-*b*-PLL diblock copolymer assembles into monodisperse micelles with ASOs.^310^

Branched polycations include a type that has randomly distributed secondary chains branching from the primary polymer backbone. There are also dendrimers—polymers with fractal branching from a core. Nucleic acid carriers include branched PEI, branched PBAEs, as well as PLL, PAMAM, and poly(propyleneimine) (PPI) dendrimers (Figure 27). Branched PEI, one of the most widely studied polycationic nucleic acid carriers, comprises primary, secondary, and tertiary amines, with different p*K*_a_ values. Branched PEI provides efficient nucleic acid binding and extensive buffering capacity, which likely contribute to its outstanding performance.^311^

PAMAM dendrimers, the most widely used dendrimer for nucleic acid delivery, comprise a hydrogen-bonding amide and tertiary amine groups in their cores and primary amine end groups in their coronas. Structural modifications have been attempted to lessen potential toxicity, enhance circulation time, and/or enhance targeting.^312−315^

Natural polymers including proteins and polysaccharides, particularly cationic collagen derivatives and chitosan, have been studied as nucleic acid delivery vehicles.^316^ Chitosan, a linear cationic polysaccharide, has been formulated into polyplexes to deliver miRNA to osteopathic tumors, for multiple myeloma, and for bone regeneration.^317−319^ An advantage of chitosan is that nucleic acids easily dissociate from the polymer upon cellular internalization.^320^ Cationic collagen derivatives have been used for nucleic acid delivery to articular cartilage, for bone regeneration, and for treating tumor metastases.^321−323^

Cyclodextrins (CDs) are natural carbohydrate polymers for the delivery of therapeutic nucleic acids^324^ that have shown clinical success.^325^ CDs are biocompatible cyclic oligosaccharides with a hydrophilic exterior surface and a hydrophobic interior cavity. Cationic CD derivatives assemble with siRNA via electrostatic interactions into stable gene delivery nanostructures of 50–200 nm.^326^

Similar to cationic lipid nanoparticles, polymeric nucleic acid carriers bind non-specifically to the negatively charged cell membrane via electrostatic interactions^327^ and are internalized by endocytosis.^328^ Following endocytosis into early endosomes, polyplexes move through the progressively more acidic endosomes (pH 6.0–4.8). Escaping endosomal confinement is critical for nucleic acid delivery; therefore, scientists are developing and modifying polymer-based systems to promote endosomal escape.^327,329−331^

### Peptides

Peptides are structurally and functionally versatile, biocompatible, and can target cells; thus, they are attractive RNA carriers. Peptides that penetrate the cellular membrane and transfer to the cytoplasm, CPPs, are used most frequently for RNA delivery (Table 13). CPPs have variable sequences, lengths, and polarities, and they can enter cells via multiple pathways, such as by forming a hole in the membrane or by endocytosis. CPPs deliver nucleic acids into cells via chemical conjugation or non-covalent complex formation with nucleic acids. Electrostatic and hydrophobic interactions between CPPs and nucleic acids result in self-assembly into peptide-based nanoparticles.^332,333^ Amphipathic CPPs have been developed using hydrophilic and hydrophobic domains to provide both nucleic acid complexation and membrane interactions.^334^ CPPs are promising, non-viral alternatives to lipid- or polymer-based carriers of therapeutic nucleic acids.^12^

**Table 13:** Examples of CPPs Used in RNA Delivery^335^

CPP	amino acid sequence
Natural CPPs	
HIV Tat	YGRKKRRQRRR
HIV Rev	TRQARRNRRRRWRERQR
FHV coat	RRRRNRTRRNRRRVR
Penetratin	RQIKIWFQNRRMKWKK
MPG	GALFLGFLGAAGSTMGAWSQPKKKRKV

Polyarginines	
PR9	FFLIPKGRRRRRRRRR
SR9	RRRRRRRRR
IR9	GLFEAIEGFIENGWEGMIDGWYGRRRRRRRRR
HR9	CHHHHHRRRRRRRRRHHHHHC

Artificial/Engineered CPPs	
Transportan	CLIKKALAALAKLNIKLLYGASNLTWG
CADY	GLWRALWRLLRSLWRLLWRA
C6	RLLRLLLRLWRRLLRLLR
PF20	LLKLLKKLLKLLKKLLKLL
NAP	KALKLKLALALLAKLKLA
POD	GGG[ARKKAAKA]4

The Tat peptide originated from the Tat protein of the human immunodeficiency virus (HIV).^336−338^ The positively charged oligoarginine peptides promote internalization by forming hydrogen bonds with the membrane sulfates and the nucleic acid phosphate groups.^339,340^ The histidine-rich peptide is efficient in siRNA delivery.^341^ The skin permeating and cell entering (SPACE) peptides facilitate the penetration of conjugated cargoes into the epidermis and dermis.^339,340,342^ Recently, fusion peptides were developed comprising SPACE and cationic oligoarginine linked by a GCG sequence.^343^ Nanocomplexes of fusion peptides and siRNA exhibited enhanced cellular uptake, gene silencing, and retention, possibly due to the synergistic effect of the oligoarginine and SPACE peptides. Oligoarginine electrostatically attracts siRNAs to form nanocomplexes, and the SPACE peptide interacts with the cellular membrane via hydrogen bonding.

Non-covalent complexation of cationic peptides that comprise hydrophilic and hydrophobic domains with RNA provide efficient gene silencing.^344,345^ The hydrophilic domain of positively charged amino acids, such as arginine, lysine, and histidine, provides a net positive charge of at least +8 that condenses the RNA and promotes hydrogen bonding with the anionic cellular membrane to stimulate cellular uptake.^346^ The hydrophobic peptide domain of tryptophan and phenylalanine enhances interactions with the lipid bilayer. Terminal modification of the cationic peptides with hydrophobic molecules such as cholesterol, stearic acid, or cholic acid increases the hydrophobicity.^347,348^ The other terminus of the peptide can be modified with hydrophilic molecules such as PEG. These peptides form a micelle-like structure with siRNA and deliver them efficiently to target cells.^347,348^

Protamines are naturally occurring cationic peptides involved in the condensation of chromatin during spermatogenesis. Comprising >65% positively charged arginines, protamines form non-covalent electrostatic complexes with nucleic acids and protect them from enzymatic degradation. Although protamines are intrinsically disordered peptides, in the presence of nucleic acids they switch from a random coil to a structure with one or more α-helices.^349^ Protamine–RNA complexes show limited efficacy, possibly due to the tight interaction between the protamine and RNA.^350^

### Other Carriers

Gold nanoparticles (AuNP) carriers are biocompatible, protect RNA from degradation, and have tunable shape, size, and optical properties.^351,352^ Two approaches for the design of AuNP nucleic acid carriers are covalent attachment and supramolecular assembly. To covalently conjugate RNA with the AuNP core, a thiol group is introduced onto the RNA^353^ to create RNA monolayers by linking RNAs directly to the AuNP surface via Au–thiol bonds. The dense shell of oligonucleotides on the surface of the AuNPs inhibits nuclease degradation. Since AuNP–RNA conjugates can be delivered transdermally, they may be useful for gene therapy to treat cutaneous tumors, skin inflammation, and other skin disorders. To electrostatically attract the negatively charged nucleic acids, AuNPs have been functionalized by structurally modifying them, adding positively charged molecules such as polymers and amino acids.^354^

Spherical nucleic acids are 3D nucleic acid nanostructures that are functionalized, packed densely, and oriented spherically around a nanoparticle core.^355^ Since they are resistant to nuclease degradation and are taken up efficiently by cells they show promise as therapeutic agents.^356^ Despite their high negative charge (zeta potential less than −30 mV), they rapidly and efficiently enter cells by caveolin-mediated endocytosis and regulate gene expression. Immunomodulatory spherical nucleic acids that stimulate or regulate immunity by engaging toll-like receptors are a promising immunotherapy for cancers including triple-negative breast cancer, prostate cancer, and melanoma.^357^

Magnetic nanoparticles deliver nucleic acid by magnetofection, which is the application of a magnetic field.^358,359^ The nanoparticles need functionalization (i.e., surface modifications) to enhance their performance as a magnetic gene delivery vector. The earliest magnetic core–shell nanoparticles for gene delivery were iron oxide nanoparticles stabilized with high molecular weight PEI, with a hydrodynamic diameter of ∼200 nm and positive zeta potential.^360^ Nucleic acids with magnetic nanoparticles increased transfection efficiency by about 1000-fold compared to non-magnetic vectors. A variety of formulations of PEI-coated superparamagnetic iron oxide nanoparticles complexed with nucleic acids have been reported.^361^ Other polymers and lipids have also been used to functionalize iron oxide nanoparticles for nucleic acid delivery.^362−366^ These include biodegradable polylactide magnetic nanoparticles containing oleate-coated magnetite and surface-modified with PEI-oleate for nucleic acid binding,^367^ and mesoporous silica nanoparticles decorated with magnetite nanocrystals to yield highly efficient transfection agents.^368^

Mesoporous silica nanoparticles (MSNs) are hollow particles that can encapsulate a wide range of drugs after surface modification. Silica nanoparticles allow slow release of the cargo as they degrade slowly in the body to non-toxic byproducts. The advantages of MSNs as drug delivery vehicles include a large surface area, tunable pore sizes, simple surface modifications, and efficient encapsulation of cargo molecules.^369^ Mesoporous and solid silica nanoparticles are synthesized using the sol–gel method in which a silica precursor is hydrolyzed and then condensed to generate spherical particles. Tetraethyl orthosilicate, the usual silica precursor, provides good control by modification of the synthesis conditions. Silica nanoparticles with a spiky nanotopography can enlarge the surface area for binding RNA molecules. To bind the negatively charged nucleic acids and enhance cellular uptake, silica particles are modified with positively charged compounds such as PLL or PEI, or with branched PEI to enhance the binding of the nucleic acids.^370^ After cell entry by endocytosis, the acidic pH in the endosomes changes the charge of PEI resulting in the dissociation and release of the RNA.

Calcium phosphate nanoparticles are promising non-viral vectors for gene therapy, an emerging strategy for effective bone repair and regeneration. The calcium phosphate naturally present in bones makes calcium phosphate nanoparticles a good choice for RNA delivery for bone repair.^371^ There is particular interest in the use of calcium phosphate nanoparticles with RNAi to regulate gene expression in bone tissue engineering because of their osteoinductivity, osteoconductivity, and affinity for nucleic acids.^372^ Calcium phosphate nanoparticles are endocytosed by cells and dissolve in the acidic endosomes and lysosomes, resulting in the release of the nucleic acid cargo.^373^ Calcium phosphate nanocarriers are biocompatible, biodegradable, non-toxic and non-immunogenic, inexpensive, and easily synthesized. However, they become unstable over time because of the growth of crystals,^371^ and they provide only limited nuclease protection. Surface functionalization of calcium phosphate particles can facilitate their endocytosis and subsequent endosomal escape.^371,374^

Although many vehicles or ligands have been used for the delivery of nucleic acids, they may exhibit low immunocompatibility, toxicity, poor stability, inefficient drug release, or restricted tissue accessibility.^375^ However, RNA itself can self-assemble into complex programmable structures.^376,377^ Ligand-conjugated RNA nanoparticles based on bacteriophage phi29 packaging RNA provide a targeted ribozyme delivery system.^378^ RNA nanoparticles can also carry CpG DNA to macrophages.^377^ Origami-like RNA nanostructures are stable and efficient as drug carriers for controlled drug release.^379,380^

Virus-like particles (VLPs) are organized protein complexes resembling a native virus capsid. VLPs are either naturally occurring empty virus shells or are synthesized by the expression of viral structural proteins that self-assemble into a virus-like structure. VLPs are attractive drug delivery platforms due to their biocompatibility, biodegradability, and targeting ability.^381^ VLPs produced *in vivo* are used for various medical applications such as drug and vaccine delivery,^382^ including the COVID-19 vaccine Novavax (NVX-CoV2373) currently in phase III trials.^383^*In vivo* and *in vitro* cargo loading has been demonstrated for VLPs.^384^

Quantum dots (QDs) are semiconductor crystalline nanoparticles with unique tunable optical properties including nearly 100-fold greater brightness and 1000-fold better stability against photobleaching versus organic dyes and luminescent proteins. Their fluorescence emission can be adjusted by the particle size, known as the quantum size effect.^385^ QDs emit narrow wavelength bands under a wide excitation range, can be appropriately functionalized, and are desirable vectors for imaging-guided therapy. Since they can efficiently deliver RNAs into target cells and can be used to track the RNA distribution in cells,^386^ they may serve as an effective theranostic RNA delivery agent.^387,388^ The application of theranostic agents with appropriate targeted, controlled delivery and imaging capabilities has the potential to significantly advance gene therapy.

Carbon nanotubes (CNTs)—tubes made of carbon with nanosized diameters—have attracted increasing attention in biomedicine because of their unique structures and properties, being the strongest and stiffest materials yet found in terms of tensile strength and elastic modulus. With functionalization, CNTs have been used as nanocarriers for nucleic acids including siRNA, oligonucleotides, and RNA/DNA aptamers.^389^ Amino-functionalized multiwalled CNTs deliver proprietary toxic siRNA to human lung carcinoma cells.^390^ Appropriately designed amino-functional segments on the CNTs promote internalization in the cell and gene silencing.^391^ Cationic single-walled CNTs deliver siRNA to Lewis lung carcinoma cells.^392^

Despite the widespread use of carriers for RNA delivery, naked RNA has also been delivered *in vivo*,^393^ including delivery of mRNA by intramuscular, subcutaneous, or intradermal injections; the latter can promote wound healing by *in situ* expression of specific proteins in the skin.^394−398^ Local injections circumvent complications associated with systemic administration such as clearance from the bloodstream.^398^ Naked mRNA administered subcutaneously produces a more efficient translation of the protein than mRNA-loaded nanoparticles.^399,400^

PEGylation allows protein drugs to avoid the immune response,^401^ and it also improves the surface properties of biomolecules and drug delivery systems. It blocks surface access by steric hindrance, increasing the circulation time in blood.^402^ Thus, it may improve pharmacokinetic properties and enhance the efficacy of drugs, including RNA therapeutics.^403^ PEGylation of the RNA nanocarriers reduces non-specific interactions with serum proteins and prevents recognition by the immune system, increasing the blood circulation time. PEGylation stabilizes RNAs, with longer PEGs stabilizing oligonucleotides better than shorter ones.^404^ The aptamer drug Macugen comprises an oligonucleotide modified with branched 40 kDa PEG at the 5′ terminus, thus enhancing the nuclease resistance of the PEG-aptamer conjugate. PEGylation markedly improves the pharmacokinetics and boosts the neutralizing activity of anti-IL-17A aptamers, an important advance in the development of therapeutic aptamers.^405^

The advantages of the various RNA carriers are summarized in Table 14.

**Table 14:** Advantages of the RNA Carriers

RNA delivery system	advantages
lipid nanoparticles	• ease of production
	• biodegradability
	• enhanced RNA stability
	• improved cellular uptake and intracellular release

polymeric nanoparticles	• versatile, adaptable, and scalable
	• low immunogenicity and cellular toxicity

peptides	• versatile
	• biocompatible
	• high target specificity

gold nanoparticles	• adaptable
	• biocompatible
	• protects RNA from degradation

exosomes	• low toxicity and immunogenicity
	• unique membrane composition enhances entrance into target cells
	• no accumulation of therapeutic RNAs in liver

magnetic nanoparticles	• highly efficient transfection efficiency

carbon nanotubes	• strong, yet highly flexible with high tensile strength

virus-like particles	• biocompatibility
	• biodegradability
	• high target specificity

silica nanoparticles	• large surface area and tunable pore size
	• simple surface modifications
	• efficient encapsulation of cargo compounds

quantum dots	• stability
	• adaptability
	• most desirable for imaging-guided therapies

PEGylation	• reduced immune response
	• improves pharmacokinetic properties
	• enhanced efficacy
	• enhanced RNA stability

self-assembled RNA/naked RNA	• highly customizable
	• can provide a more efficient translation than mRNA-loaded nanoparticles for certain routes of drug administration

## Conclusions

Our understanding of the types and functions of RNA has increased dramatically over the past 50 years. Originally identified simply as coding or non-coding, RNA was recognized early as central to the process of transcribing and translating a gene into a protein. Over the last few decades, many new types of RNA have been discovered with specific functions, including snRNA, snoRNA, shRNA, miRNA, tmRNA, siRNA, saRNA, piRNA, and circRNA. These RNA molecules participate in a complex network that regulates how RNA is used in the cell and modifies gene expression. The multiple functions of RNA provide multiple pathways for exploiting RNA as a therapeutic molecule.

RNA can be engineered for its intended therapeutic functions by *in vitro* transcription or solid-support synthesis. Therapeutic mRNAs, which are translated *in vivo* to deliver a therapeutic or vaccine protein, require extensive replacement of uridine with 1-methylpseudouridine to decrease the immune response to the mRNA and improve the translation. ASOs and therapeutic siRNAs have phosphorothioate and 2′-ribose modifications to protect the RNA from degradation and to improve target specificity. Aptamers, whose 3D structure rather than primary sequence determines their function, are heavily modified. For all RNAs, the degree of modification can be adjusted to enhance the effectiveness of the therapeutic RNA.

There are now approved RNA medicines for cardiovascular, metabolic, liver, infectious, neurological, neuromuscular, kidney, and eye diseases, with many more in the research phase. Although there are many types of therapeutic RNAs, ASOs dominate the approved drugs, followed by siRNAs. CRISPR and AOC therapeutics currently have high research interests, still in the pre-clinical stage. The top RNA medicine companies such as Moderna, Ionis, BioNTech, Alnylam, and Sirnaomics all specialize in one type of RNA, but research and treat as many as nine different diseases. RNA therapeutics have the potential to treat a wide range of diseases, from the most common to the extremely rare.

RNA carriers are important in overcoming the physiological barriers of systemic administration of RNA-based drugs. However, they provide a challenge in the translation of RNA medicines to the clinical setting. After the recent success of the lipid nanoparticle-based mRNA vaccines against COVID-19, lipid carriers have become the primary RNA delivery vehicle. However, since lipid nanoparticles can be toxic to cells and stimulate the release of systemic inflammatory cytokines, interest in natural transporters such as exosomes is gaining momentum. Other non-toxic and non-immunogenic carriers, such as structurally and functionally versatile peptides, are also attracting attention. Novel technologies, including carbon nanotubes and other inorganic biocompatible polymers, carbon nanodots, and functionalized or hybrid systems, are also exciting avenues of exploration and evaluation.

Our growing understanding of the many types and functions of RNA has been combined with the ability to synthesize modified RNAs with improved stability and pharmaceutical activity. Nanotechnology-based systems to deliver those RNAs to the cell have resulted in an explosion of new therapeutic options for diseases ranging from viral infections to cancer. This arsenal of multiple specifically targeted RNA therapies has the potential to revolutionize the treatment of human disease.

## Supporting Information Available

Figure S1, number of documents for various RNA types applied in medical studies in the years of 1995–2020, categorized by the type of diseases; Figure S2, top companies with RNA therapeutics and vaccines in clinical trials; Figure S3, frequencies of various types of modifications on RNA sequences and their distributions with respect to disease types obtained from the CAS Content Collection; Figure S4, frequencies of various types of modifications on RNA sequences and their distributions with respect to specific diseases acquired from the CAS Content Collection; Figure S5, the co-occurrence of RNA modifications on the same sequences; Figure S6, number of documents per year related to modified RNAs (data were obtained from a SciFinder^n^ search); Figure S7, modified and rare nucleic acid bases; Table S1, timeline and milestones of RNA research and development (PDF)
Table S2, listing RNA therapies and vaccines for various diseases in the development stages (XLSX)

## Abbreviations Used

AAT: alpha-1 antitrypsin
AATD: alpha-1 antitrypsin deficiency
AOC: antibody–oligonucleotide conjugate
APOCIII: apolipoprotein C-III
ASO: antisense oligomer
asRNA: antisense RNA
AUD: alcohol use disorder
circRNA: circular RNA
CMV: cytomegalovirus
CNT: carbon nanotube
CRISPR: clustered regularly interspaced short palindromic repeats
crRNA: CRISPR RNA
DMD: Duchenne muscular dystrophy
FDA: U.S. Food and Drug Administration
GNA: glycol nucleic acid
LNA: locked nucleic acid
lncRNA: long non-coding RNA
miRNA: micro-RNA
MOE: 2′-*O*-(2-methoxyethyl)
mRNA: messenger RNA
ncRNA: non-coding RNA
PAA: poly(amidoamine)
PAEMA: poly(2-aminoethyl methacrylamide)
PBAE: poly(β-amino ester)
PDMAEMA: poly(2-(dimethylamino)ethyl methacrylate)
PEI: polyethylene imine
piRNA: piwi-interacting RNA
PLL: poly(l-lysine)
PMO: phosphorodiamidate morpholino oligonucleotide
PNA: peptide nucleic acid
RISC: RNA-induced silencing complex
RNAi: RNA interference
rRNA: ribosomal RNA
RSV: respiratory syncytial virus
RT-qPCR: reverse transcriptase-quantitative polymerase chain reaction
saRNA: small activating RNA
SELEX: systematic evolution of ligands by exponential enrichment
sgRNA: single guide RNA
shRNA: short hairpin RNA
siRNA: short/small interfering RNA
snoRNA: small nucleolar RNA
snRNA: small nuclear RNA
tmRNA: transfer messenger RNA
tracrRNA: trans-activating CRISPR RNA
tRNA: transfer RNA

## References

[ref1] Titze-de-Almeida S. S.; Brandão P. R. d. P.; Faber I.; Titze-de-Almeida R. Leading RNA Interference Therapeutics Part 1: Silencing Hereditary Transthyretin Amyloidosis, with a Focus on Patisiran. Molecular Diagnosis Therapy 2020, 24, 49–59. 10.1007/s40291-019-00434-w. 31701435

[ref2] Dolgin E. Injection of Hope. Nature 2019, 574, S10–S12. 10.1038/d41586-019-03072-8. 31619807

[ref3] Ozata D. M.; Gainetdinov I.; Zoch A.; O’Carroll D.; Zamore P. D. Piwi-Interacting RNAs: Small RNAs with Big Functions. Nat. Rev. Genet. 2019, 20, 89–108. 10.1038/s41576-018-0073-3. 30446728

[ref4] Ramat A.; Simonelig M. Functions of Piwi Proteins in Gene Regulation: New Arrows Added to the piRNA Quiver. Trends Genet. 2021, 37, 188–200. 10.1016/j.tig.2020.08.011. 32951946

[ref5] Yoon S.; Rossi J. J. Therapeutic Potential of Small Activating RNAs (saRNAs in Human Cancers. Curr. Pharm. Biotechnol. 2018, 19, 604–610. 10.2174/1389201019666180528084059. 29804529 PMC6204660

[ref6] Jiang F.; Doudna J. A. CRISPR–Cas9 Structures and Mechanisms. Annu. Rev. Biophys. 2017, 46, 505–529. 10.1146/annurev-biophys-062215-010822. 28375731

[ref7] Yao R. W.; Wang Y.; Chen L. L. Cellular Functions of Long Noncoding RNAs. Nat. Cell Biol. 2019, 21, 542–551. 10.1038/s41556-019-0311-8. 31048766

[ref8] Li X.; Yang L.; Chen L. L. The Biogenesis, Functions, and Challenges of Circular RNAs. Mol. Cell 2018, 71, 428–442. 10.1016/j.molcel.2018.06.034. 30057200

[ref9] Wong E.; Goldberg T. Mipomersen (Kynamro): A Novel Antisense Oligonucleotide Inhibitor for the Management of Homozygous Familial Hypercholesterolemia. P & T: a peer-reviewed journal for formulary management 2014, 39, 119–122. 24669178 PMC3956393

[ref10] Davis M. E.; Zuckerman J. E.; Choi C. H. J.; Seligson D.; Tolcher A.; Alabi C. A.; Yen Y.; Heidel J. D.; Ribas A. Evidence of RNAi in Humans from Systemically Administered siRNA Via Targeted Nanoparticles. Nature 2010, 464, 1067–1070. 10.1038/nature08956. 20305636 PMC2855406

[ref11] Whitehead K. A.; Langer R.; Anderson D. G. Knocking Down Barriers: Advances in siRNA Delivery. Nat. Rev. Drug Discov. 2009, 8, 129–138. 10.1038/nrd2742. 19180106 PMC7097568

[ref12] Roberts T. C.; Langer R.; Wood M. J. A. Advances in Oligonucleotide Drug Delivery. Nat. Rev. Drug Discov. 2020, 19, 673–694. 10.1038/s41573-020-0075-7. 32782413 PMC7419031

[ref13] Brenner S.; Meselson M.; Jacob F. An Unstable Intermediate Carrying Information from Genes to Ribosomes for Protein Synthesis. Nature 1961, 190, 576–581. 10.1038/190576a0. 20446365

[ref14] Muthukrishnan S.; Both G. W.; Furuichi Y.; Shatkin A. J. 5′-Terminal 7-Methylguanosine in Eukaryotic Messenger-RNA Is Required for Translation. Nature 1975, 255, 33–37. 10.1038/255033a0. 165427

[ref15] Furuichi Y.; Miura K. I. Blocked Structure at 5′ Terminus of Messenger-RNA from Cytoplasmic Polyhedrosis-Virus. Nature 1975, 253, 374–375. 10.1038/253374a0. 163011

[ref16] Dimitriadis G. J. Translation of Rabbit Globin mRNA Introduced by Liposomes into Mouse Lymphocytes. Nature 1978, 274, 923–924. 10.1038/274923a0. 683336

[ref17] Zamecnik P. C.; Stephenson M. L. Inhibition of Rous Sarcoma Virus Replication and Cell Transformation by a Specific Oligodeoxynucleotide. Proc. Natl. Acad. Sci. U. S. A. 1978, 75, 280–284. 10.1073/pnas.75.1.280. 75545 PMC411230

[ref18] Krieg P. A.; Melton D. A. Functional Messenger-RNAs Are Produced by SP6 Invitro Transcription of Cloned cDNAs. Nucleic Acids Res. 1984, 12, 7057–7070. 10.1093/nar/12.18.7057. 6207484 PMC320142

[ref19] Felgner P. L.; Gadek T. R.; Holm M.; Roman R.; Chan H. W.; Wenz M.; Northrop J. P.; Ringold G. M.; Danielsen M. Lipofection - a Highly Efficient, Lipid-Mediated DNA-Transfection Procedure. Proc. Natl. Acad. Sci. U. S. A. 1987, 84, 7413–7417. 10.1073/pnas.84.21.7413. 2823261 PMC299306

[ref20] Malone R. W.; Felgner P. L.; Verma I. M. Cationic Liposome-Mediated RNA Transfection. Proc. Natl. Acad. Sci. U. S. A. 1989, 86, 6077–6081. 10.1073/pnas.86.16.6077. 2762315 PMC297778

[ref21] Fire A.; Xu S. Q.; Montgomery M. K.; Kostas S. A.; Driver S. E.; Mello C. C. Potent and Specific Genetic Interference by Double-Stranded RNA in Caenorhabditis Elegans. Nature 1998, 391, 806–811. 10.1038/35888. 9486653

[ref22] Piascik P. Fomiversen Sodium Approved to Treat CMV Retinitis. J. Am. Pharm. Assoc. (Wash) 1999, 39, 84–85. 10.1016/S1086-5802(16)30428-4. 9990192

[ref23] Bernstein E.; Caudy A. A.; Hammond S. M.; Hannon G. J. Role for a Bidentate Ribonuclease in the Initiation Step of RNA Interference. Nature 2001, 409, 363–366. 10.1038/35053110. 11201747

[ref24] Kariko K.; Buckstein M.; Ni H. P.; Weissman D. Suppression of RNA Recognition by Toll-Like Receptors: The Impact of Nucleoside Modification and the Evolutionary Origin of RNA. Immunity 2005, 23, 165–175. 10.1016/j.immuni.2005.06.008. 16111635

[ref25] Weide B.; Pascolo S.; Scheel B.; Derhovanessian E.; Pflugfelder A.; Eigentler T. K.; Pawelec G.; Hoerr I.; Rammensee H.-G.; Garbe C. Direct Injection of Protamine-Protected mRNA: Results of a Phase 1/2 Vaccination Trial in Metastatic Melanoma Patients. J. Immunotherapy 2009, 32, 498–507. 10.1097/CJI.0b013e3181a00068. 19609242

[ref26] Adams D.; Gonzalez-Duarte A.; O’Riordan W. D.; Yang C. C.; Ueda M.; Kristen A. V.; Tournev I.; Schmidt H. H.; Coelho T.; Berk J. L.; Lin K. P.; Vita G.; Attarian S.; Planté-Bordeneuve V.; Mezei M. M.; Campistol J. M.; Buades J.; Brannagan T. H. III; Kim B. J.; Oh J.; Parman Y.; Sekijima Y.; Hawkins P. N.; Solomon S. D.; Polydefkis M.; Dyck P. J.; Gandhi P. J.; Goyal S.; Chen J.; Strahs A. L.; Nochur S. V.; Sweetser M. T.; Garg P. P.; Vaishnaw A. K.; Gollob J. A.; Suhr O. B. Patisiran, an RNAi Therapeutic, for Hereditary Transthyretin Amyloidosis. N. Engl. J. Med. 2018, 379, 11–21. 10.1056/NEJMoa1716153. 29972753

[ref27] Allen D.; Rosenberg M.; Hendel A. Using Synthetically Engineered Guide RNAs to Enhance CRISPR Genome Editing Systems in Mammalian Cells. Front. Genome Editing 2021, 2, 617910 10.3389/fgeed.2020.617910. PMC8525374 34713240

[ref28] Vaccines and Related Biological Products Advisory Committee Meeting. Moderna COVID-19 Vaccine. Fda Briefing Document. https://www.fda.gov/media/144434/download (accessed December 22, 2020).

[ref29] Emergency Use Authorization (Eua) of the Pfizer-Biontech COVID-19 Vaccine to Prevent Coronavirus Disease 2019 (COVID-19) in Individuals 16 Years of Age and Older. https://www.fda.gov/media/144414/download (accessed December 22, 2020).

[ref30] Fda Approves First COVID-19 Vaccine. https://www.fda.gov/news-events/press-announcements/fda-approves-first-covid-19-vaccine (accessed Dec 27, 2021).

[ref31] Castanotto D.; Zhang X.; Rüger J.; Alluin J.; Sharma R.; Pirrotte P.; Joenson L.; Ioannou S.; Nelson M. S.; Vikeså J.; Hansen B. R.; Koch T.; Jensen M. A.; Rossi J. J.; Stein C. A. A Multifunctional Lna Oligonucleotide-Based Strategy Blocks Ar Expression and Transactivation Activity in PCa Cells. Molecular Therapy - Nucleic Acids 2021, 23, 63–75. 10.1016/j.omtn.2020.10.032. 33335793 PMC7723773

[ref32] CAS Content Collection. https://www.cas.org/about/cas-content (accessed Jan 5, 2022).

[ref33] Cooper G. M., RNA Synthesis and Processing. In The Cell: A Molecular Approach; Sinauer Associates: Sunderland, MA, 2000.

[ref34] Alberts B.; Johnson A.; Lewis J.; et al. How Cells Read the Genome: From DNA to Protein. In Molecular Biology of the Cell, 4th ed.; New York: Garland Science, New York 2002.

[ref35] Valadkhan S.; Gunawardane L. S. Role of Small Nuclear RNAs in Eukaryotic Gene Expression. Essays Biochem. 2013, 54, 79–90. 10.1042/bse0540079. 23829528 PMC11246792

[ref36] Matera A. G.; Terns R. M.; Terns M. P. Non-Coding RNAs: Lessons from the Small Nuclear and Small Nucleolar RNAs. Nat. Rev. Mol. Cell Biol. 2007, 8, 209–220. 10.1038/nrm2124. 17318225

[ref37] Morais P.; Adachi H.; Yu Y. T. Spliceosomal snRNA Epitranscriptomics. Front. Genet. 2021, 12, 652129 10.3389/fgene.2021.652129. 33737950 PMC7960923

[ref38] Wajahat M.; Bracken C. P.; Orang A. Emerging Functions for snoRNAs and snoRNA-Derived Fragments. Int. J. Mol. Sci. 2021, 22, 10193 10.3390/ijms221910193. 34638533 PMC8508363

[ref39] van der Werf J.; Chin C. V.; Fleming N. I. snoRNA in Cancer Progression, Metastasis and Immunotherapy Response. Biology (Basel) 2021, 10, 809 10.3390/biology10080809. 34440039 PMC8389557

[ref40] Liang J.; Wen J.; Huang Z.; Chen X. P.; Zhang B. X.; Chu L. Small Nucleolar RNAs: Insight into Their Function in Cancer. Front. Oncol. 2019, 9, 587 10.3389/fonc.2019.00587. 31338327 PMC6629867

[ref41] Roy K.; Chanfreau G. F. The Diverse Functions of Fungal RNAse Iii Enzymes in RNA Metabolism. Enzymes 2012, 31, 213–235. 10.1016/B978-0-12-404740-2.00010-0. 27166447

[ref42] Williams G. T.; Farzaneh F. Are snoRNAs and snoRNA Host Genes New Players in Cancer?. Nat. Rev. Cancer 2012, 12, 84–88. 10.1038/nrc3195. 22257949

[ref43] Holley C. L.; Topkara V. K. An Introduction to Small Non-Coding RNAs: miRNA and snoRNA. Cardiovasc. Drugs Ther. 2011, 25, 151–159. 10.1007/s10557-011-6290-z. 21573765

[ref44] Bachellerie J. P.; Cavaillé J.; Hüttenhofer A. The Expanding snoRNA World. Biochimie 2002, 84, 775–790. 10.1016/S0300-9084(02)01402-5. 12457565

[ref45] Ro-Choi T. S. Nucleolar snoRNA and Ribosome Production. Mol. Cells 1997, 7, 451–467. 9339887

[ref46] Bridges M. C.; Daulagala A. C.; Kourtidis A. Lnccation: lncRNA Localization and Function. J. Cell Biol. 2021, 220, e202009045 10.1083/jcb.202009045. 33464299 PMC7816648

[ref47] Ferrè F.; Colantoni A.; Helmer-Citterich M. Revealing Protein-lncRNA Interaction. Brief Bioinform. 2016, 17, 106–116. 10.1093/bib/bbv031. 26041786 PMC4719072

[ref48] Jathar S.; Kumar V.; Srivastava J.; Tripathi V. Technological Developments in lncRNA Biology. Adv. Exp. Med. Biol. 2017, 1008, 283–323. 10.1007/978-981-10-5203-3_10. 28815544

[ref49] Li J.; Meng H.; Bai Y.; Wang K. Regulation of lncRNA and Its Role in Cancer Metastasis. Oncol. Res. 2016, 23, 205–217. 10.3727/096504016X14549667334007. 27098144 PMC7838649

[ref50] Peng W. X.; Koirala P.; Mo Y. Y. lncRNA-Mediated Regulation of Cell Signaling in Cancer. Oncogene 2017, 36, 5661–5667. 10.1038/onc.2017.184. 28604750 PMC6450570

[ref51] Statello L.; Guo C. J.; Chen L. L.; Huarte M. Gene Regulation by Long Non-Coding RNAs and Its Biological Functions. Nat. Rev. Mol. Cell Biol. 2021, 22, 96–118. 10.1038/s41580-020-00315-9. 33353982 PMC7754182

[ref52] Yang Z.; Jiang S.; Shang J.; Jiang Y.; Dai Y.; Xu B.; Yu Y.; Liang Z.; Yang Y. lncRNA: Shedding Light on Mechanisms and Opportunities in Fibrosis and Aging. Ageing Res. Rev. 2019, 52, 17–31. 10.1016/j.arr.2019.04.001. 30954650

[ref53] Backes C.; Meese E.; Keller A. Specific miRNA Disease Biomarkers in Blood, Serum and Plasma: Challenges and Prospects. Mol. Diagn. Ther. 2016, 20, 509–518. 10.1007/s40291-016-0221-4. 27378479

[ref54] Bernardo B. C.; Ooi J. Y.; Lin R. C.; McMullen J. R. miRNA Therapeutics: A New Class of Drugs with Potential Therapeutic Applications in the Heart. Future Med. Chem. 2015, 7, 1771–1792. 10.4155/fmc.15.107. 26399457

[ref55] Cai Y.; Yu X.; Hu S.; Yu J. A Brief Review on the Mechanisms of miRNA Regulation. Genomics Proteomics Bioinformatics 2009, 7, 147–154. 10.1016/S1672-0229(08)60044-3. 20172487 PMC5054406

[ref56] Cao T.; Zhen X. C. Dysregulation of miRNA and Its Potential Therapeutic Application in Schizophrenia. CNS Neurosci. Ther. 2018, 24, 586–597. 10.1111/cns.12840. 29529357 PMC6490029

[ref57] Lee Y. S.; Dutta A. Micrornas in Cancer. Annu. Rev. Pathol. 2009, 4, 199–227. 10.1146/annurev.pathol.4.110807.092222. 18817506 PMC2769253

[ref58] Lu T. X.; Rothenberg M. E. Microrna. J. Allergy Clin. Immunol. 2018, 141, 1202–1207. 10.1016/j.jaci.2017.08.034. 29074454 PMC5889965

[ref59] Tiwari A.; Mukherjee B.; Dixit M. Microrna Key to Angiogenesis Regulation: miRNA Biology and Therapy. Curr. Cancer Drug Targets 2018, 18, 266–277. 10.2174/1568009617666170630142725. 28669338

[ref60] Piatek M. J.; Werner A. Endogenous siRNAs: Regulators of Internal Affairs. Biochem. Soc. Trans. 2014, 42, 1174–1179. 10.1042/BST20140068. 25110021 PMC4289821

[ref61] Crooke S. T.; Witztum J. L.; Bennett C. F.; Baker B. F. RNA-Targeted Therapeutics. Cell Metab. 2018, 27, 714–739. 10.1016/j.cmet.2018.03.004. 29617640

[ref62] Schütze N. siRNA Technology. Mol. Cell. Endocrinol. 2004, 213, 115–119. 10.1016/j.mce.2003.10.078. 15062558

[ref63] Rossi J. J.; Rossi D. J. siRNA Drugs: Here to Stay. Mol. Ther. 2021, 29, 431–432. 10.1016/j.ymthe.2021.01.015. 33472033 PMC7854346

[ref64] Pushparaj P. N.; Aarthi J. J.; Manikandan J.; Kumar S. D. siRNA, miRNA, and shRNA: In Vivo Applications. J. Dent. Res. 2008, 87, 992–1003. 10.1177/154405910808701109. 18946005

[ref65] Qureshi A.; Tantray V. G.; Kirmani A. R.; Ahangar A. G. A Review on Current Status of Antiviral siRNA. Rev. Med. Virol 2018, 28, e1976 10.1002/rmv.1976. 29656441 PMC7169094

[ref66] Hu B.; Weng Y.; Xia X. H.; Liang X. J.; Huang Y. Clinical Advances of siRNA Therapeutics. J. Gene Med. 2019, 21, e3097 10.1002/jgm.3097. 31069898

[ref67] Gavrilov K.; Saltzman W. M. Therapeutic siRNA: Principles, Challenges, and Strategies. Yale J. Biol. Med. 2012, 85, 187–200. 22737048 PMC3375670

[ref68] Hu B.; Zhong L.; Weng Y.; Peng L.; Huang Y.; Zhao Y.; Liang X. J. Therapeutic siRNA: State of the Art. Signal Transduct. Target. Ther. 2020, 5, 101 10.1038/s41392-020-0207-x. 32561705 PMC7305320

[ref69] Singh A.; Trivedi P.; Jain N. K. Advances in siRNA Delivery in Cancer Therapy. Artif Cells Nanomed. Biotechnol. 2018, 46, 274–283. 10.1080/21691401.2017.1307210. 28423924

[ref70] Nikam R. R.; Gore K. R. Journey of siRNA: Clinical Developments and Targeted Delivery. Nucleic Acid Ther. 2018, 28, 209–224. 10.1089/nat.2017.0715. 29584585

[ref71] Saw P. E.; Song E. W. siRNA Therapeutics: A Clinical Reality. Sci. China Life Sci. 2020, 63, 485–500. 10.1007/s11427-018-9438-y. 31054052

[ref72] Czech B.; Munafò M.; Ciabrelli F.; Eastwood E. L.; Fabry M. H.; Kneuss E.; Hannon G. J. piRNA-Guided Genome Defense: From Biogenesis to Silencing. Annu. Rev. Genet. 2018, 52, 131–157. 10.1146/annurev-genet-120417-031441. 30476449 PMC10784713

[ref73] Ishizu H.; Nagao A.; Siomi H. Gatekeepers for Piwi-piRNA Complexes to Enter the Nucleus. Curr. Opin. Genet. Dev. 2011, 21, 484–490. 10.1016/j.gde.2011.05.001. 21764576

[ref74] Iwasaki Y. W.; Siomi M. C.; Siomi H. Piwi-Interacting RNA: Its Biogenesis and Functions. Annu. Rev. Biochem. 2015, 84, 405–433. 10.1146/annurev-biochem-060614-034258. 25747396

[ref75] Lenart P.; Novak J.; Bienertova-Vasku J. Piwi-piRNA Pathway: Setting the Pace of Aging by Reducing DNA Damage. Mech. Ageing Dev. 2018, 173, 29–38. 10.1016/j.mad.2018.03.009. 29580825

[ref76] Liu Y.; Dou M.; Song X.; Dong Y.; Liu S.; Liu H.; Tao J.; Li W.; Yin X.; Xu W. The Emerging Role of the piRNA/piwi Complex in Cancer. Mol. Cancer 2019, 18, 123 10.1186/s12943-019-1052-9. 31399034 PMC6688334

[ref77] Mani S. R.; Juliano C. E. Untangling the Web: The Diverse Functions of the piwi/piRNA Pathway. Mol. Reprod. Dev. 2013, 80, 632–664. 10.1002/mrd.22195. 23712694 PMC4234069

[ref78] Yuan Z. H.; Zhao Y. M. The Regulatory Functions of piRNA/piwi in Spermatogenesis. Yi Chuan 2017, 39, 683–691. 10.16288/j.yczz.17-245. 28903896

[ref79] Chang W.; Wang J. Exosomes and Their Noncoding RNA Cargo Are Emerging as New Modulators for Diabetes Mellitus. Cells 2019, 8, 853 10.3390/cells8080853. PMC6721737 31398847

[ref80] Chu Y. L.; Li H.; Ng P. L. A.; Kong S. T.; Zhang H.; Lin Y.; Tai W. C. S.; Yu A. C. S.; Yim A. K. Y.; Tsang H. F.; Cho W. C. S.; Wong S. C. C. The Potential of Circulating Exosomal RNA Biomarkers in Cancer. Expert Rev. Mol. Diagn. 2020, 20, 665–678. 10.1080/14737159.2020.1745064. 32188269

[ref81] Mathivanan S.; Ji H.; Simpson R. J. Exosomes: Extracellular Organelles Important in Intercellular Communication. J. Proteomics 2010, 73, 1907–1920. 10.1016/j.jprot.2010.06.006. 20601276

[ref82] Xie Y.; Dang W.; Zhang S.; Yue W.; Yang L.; Zhai X.; Yan Q.; Lu J. The Role of Exosomal Noncoding RNAs in Cancer. Mol. Cancer 2019, 18, 37 10.1186/s12943-019-0984-4. 30849983 PMC6408816

[ref83] Zhang J.; Li S.; Li L.; Li M.; Guo C.; Yao J.; Mi S. Exosome and Exosomal Microrna: Trafficking, Sorting, and Function. Genomics Proteomics Bioinformatics 2015, 13, 17–24. 10.1016/j.gpb.2015.02.001. 25724326 PMC4411500

[ref84] Crick F. H. On Protein Synthesis. Symp. Soc. Exp Biol. 1958, 12, 138–163. 13580867

[ref85] Palade G. E. A Small Particulate Component of the Cytoplasm. Journal of Biophysical and Biochem. Cytol. 1955, 1, 59–68. 10.1083/jcb.1.1.59. PMC2223592 14381428

[ref86] Holley R. W.; Apgar J.; Everett G. A.; Madison J. T.; Marquisee M.; Merrill S. H.; Penswick J. R.; Zamir A. Structure of a Ribonucleic Acid. Science 1965, 147, 1462–1465. 10.1126/science.147.3664.1462. 14263761

[ref87] Esteller M. Non-Coding RNAs in Human Disease. Nat. Rev. Genet. 2011, 12, 861–874. 10.1038/nrg3074. 22094949

[ref88] Hombach S.; Kretz M. Non-Coding RNAs: Classification, Biology and Functioning. Adv. Exp. Med. Biol. 2016, 937, 3–17. 10.1007/978-3-319-42059-2_1. 27573892

[ref89] Yang J. X.; Rastetter R. H.; Wilhelm D. Non-Coding RNAs: An Introduction. Adv. Exp. Med. Biol. 2016, 886, 13–32. 10.1007/978-94-017-7417-8_2. 26659485

[ref90] Xu J.; Bai J.; Xiao J. Computationally Modeling ncRNA-ncRNA Crosstalk. Adv. Exp. Med. Biol. 2018, 1094, 77–86. 10.1007/978-981-13-0719-5_8. 30191489

[ref91] Liu N.; Wang Z. Z.; Zhao M.; Zhang Y.; Chen N. H. Role of Non-Coding RNA in the Pathogenesis of Depression. Gene 2020, 735, 144276 10.1016/j.gene.2019.144276. 31816363

[ref92] Anastasiadou E.; Jacob L. S.; Slack F. J. Non-Coding RNA Networks in Cancer. Nat. Rev. Cancer 2018, 18, 5–18. 10.1038/nrc.2017.99. 29170536 PMC6337726

[ref93] Panni S.; Lovering R. C.; Porras P.; Orchard S. Non-Coding RNA Regulatory Networks. Biochim. Biophys. Acta Gene Regul. Mech. 2020, 1863, 194417 10.1016/j.bbagrm.2019.194417. 31493559

[ref94] Mattick J. S.; Makunin I. V. Non-Coding RNA. Hum. Mol. Genet. 2006, 15, R17–R29. 10.1093/hmg/ddl046. 16651366

[ref95] Jiao A. L.; Slack F. J. RNA-Mediated Gene Activation. Epigenetics 2014, 9, 27–36. 10.4161/epi.26942. 24185374 PMC3928182

[ref96] Kwok A.; Raulf N.; Habib N. Developing Small Activating RNA as a Therapeutic: Current Challenges and Promises. Ther. Deliv. 2019, 10, 151–164. 10.4155/tde-2018-0061. 30909853

[ref97] Laham-Karam N.; Laitinen P.; Turunen T. A.; Ylä-Herttuala S. Activating the Chromatin by Noncoding RNAs. Antioxid. Redox Signal. 2018, 29, 813–831. 10.1089/ars.2017.7248. 28699365

[ref98] Li L. C. Small RNA-Guided Transcriptional Gene Activation (RNAa) in Mammalian Cells. Adv. Exp. Med. Biol. 2017, 983, 1–20. 10.1007/978-981-10-4310-9_1. 28639188

[ref99] Li S. M.; Hu J. Small Activating RNAs: Towards Development of New Therapeutic Agents and Clinical Treatments. Curr. Pharm. Biotechnol. 2018, 19, 622–630. 10.2174/1389201019666180802145134. 30070178

[ref100] Zheng L.; Wang L.; Gan J.; Zhang H. RNA Activation: Promise as a New Weapon against Cancer. Cancer Lett. 2014, 355, 18–24. 10.1016/j.canlet.2014.09.004. 25261049

[ref101] Zhao X.; Voutila J.; Habib N.; et al. RNA Activation. In Innovative Medicine: Basic Research and Development; Nakao K., Minato N., Uemoto S., Eds.; Springer: Tokyo, 2015. 29787043

[ref102] Yu C. Y.; Kuo H. C. The Emerging Roles and Functions of Circular RNAs and Their Generation. J. Biomed Sci. 2019, 26, 29 10.1186/s12929-019-0523-z. 31027496 PMC6485060

[ref103] Eguchi Y.; Itoh T.; Tomizawa J.-i. Antisense RNA. Annu. Rev. Biochem. 1991, 60, 631–652. 10.1146/annurev.bi.60.070191.003215. 1715680

[ref104] Damase T. R.; Sukhovershin R.; Boada C.; Taraballi F.; Pettigrew R. I.; Cooke J. P. The Limitless Future of RNA Therapeutics. Front. Bioeng. Biotechnol. 2021, 9, 628137 10.3389/fbioe.2021.628137. 33816449 PMC8012680

[ref105] Hanna J.; Hossain G. S.; Kocerha J. The Potential for Microrna Therapeutics and Clinical Research. Front. Genet. 2019, 10, 00478 10.3389/fgene.2019.00478. PMC6532434 31156715

[ref106] Bobba C. M.; Fei Q.; Shukla V.; Lee H.; Patel P.; Putman R. K.; Spitzer C.; Tsai M.; Wewers M. D.; Lee R. J.; Christman J. W.; Ballinger M. N.; Ghadiali S. N.; Englert J. A. Nanoparticle Delivery of Microrna-146a Regulates Mechanotransduction in Lung Macrophages and Mitigates Injury During Mechanical Ventilation. Nat. Commun. 2021, 12, 289 10.1038/s41467-020-20449-w. 33436554 PMC7804938

[ref107] Dovgan I.; Koniev O.; Kolodych S.; Wagner A. Antibody–Oligonucleotide Conjugates as Therapeutic, Imaging, and Detection Agents. Bioconjugate Chem. 2019, 30, 2483–2501. 10.1021/acs.bioconjchem.9b00306. 31339691

[ref108] DeLong R. Ushering in a New Era of RNA-Based Therapies. Commun. Biol. 2021, 4, 577 10.1038/s42003-021-02150-w. 34002014 PMC8129104

[ref109] Liu D. R.; Levy J.; Ma Y.; Wei H.; Comander J.; Tachida Y.; Pierce E. A.; Liu Q.; Pendse N. Targeted Base Editing of the Ush2a Gene. Patent WO2021222318, 2021.

[ref110] Darmostuk M.; Rimpelova S.; Gbelcova H.; Ruml T. Current Approaches in SELEX: An Update to Aptamer Selection Technology. Biotechnol. Adv. 2015, 33, 1141–1161. 10.1016/j.biotechadv.2015.02.008. 25708387

[ref111] Lamberti G.; Barba A. A. Drug Delivery of siRNA Therapeutics. Pharmaceutics 2020, 12, 178 10.3390/pharmaceutics12020178. PMC7076510 32093141

[ref112] Heiser A.; Coleman D.; Dannull J.; Yancey D.; Maurice M. A.; Lallas C. D.; Dahm P.; Niedzwiecki D.; Gilboa E.; Vieweg J. Autologous Dendritic Cells Transfected with Prostate-Specific Antigen RNA Stimulate CTL Responses against Metastatic Prostate Tumors. J. Clin. Invest. 2002, 109, 409–417. 10.1172/JCI0214364. 11828001 PMC150859

[ref113] Sahin U.; Karikó K.; Türeci Ö. mRNA-Based Therapeutics — Developing a New Class of Drugs. Nat. Rev. Drug Discovery 2014, 13, 759–780. 10.1038/nrd4278. 25233993

[ref114] Saeedi S.; Israel S.; Nagy C.; Turecki G. The Emerging Role of Exosomes in Mental Disorders. Transl. Psychiatry 2019, 9, 122 10.1038/s41398-019-0459-9. 30923321 PMC6438960

[ref115] Condrat C. E.; Thompson D. C.; Barbu M. G.; Bugnar O. L.; Boboc A.; Cretoiu D.; Suciu N.; Cretoiu S. M.; Voinea S. C. miRNAs as Biomarkers in Disease: Latest Findings Regarding Their Role in Diagnosis and Prognosis. Cells 2020, 9, 276 10.3390/cells9020276. PMC7072450 31979244

[ref116] Tribolet L.; Kerr E.; Cowled C.; Bean A. G. D.; Stewart C. R.; Dearnley M.; Farr R. J. MicroRNA Biomarkers for Infectious Diseases: From Basic Research to Biosensing. Front. Microbiol. 2020, 11, 01197 10.3389/fmicb.2020.01197. PMC7286131 32582115

[ref117] Ravkin H. D.; Givton O.; Geffen D. B.; Rubin E. Direct Comparison Shows That mRNA-Based Diagnostics Incorporate Information Which Cannot Be Learned Directly from Genomic Mutations. BMC Bioinformatics 2020, 21, 196 10.1186/s12859-020-3512-z. 32429832 PMC7236449

[ref118] Kaminski M. M.; Abudayyeh O. O.; Gootenberg J. S.; Zhang F.; Collins J. J. CRISPR-Based Diagnostics. Nat. Biomed. Eng. 2021, 5, 643–656. 10.1038/s41551-021-00760-7. 34272525

[ref119] Mermade 192x Synthesizer. https://shop.biosearchtech.com/nucleic-acid-chemistry-reagents-and-instruments/dna-and-rna-synthesis-instruments-and-accessories/dna-and-rna-oligonucleotide-synthesizers/mermade-192x/mermade-192x-synthesizer/p/NACINS-007 (accessed Nov 20, 2021).

[ref120] Tremblay F.; Bondurant L. D.; McIninch J. D.; Castoreno A.; Schlegel M. K.; Kaittanis C. New Double Stranded RNA Agent Capable of Inhibiting Expression of Apolipoprotein C3 in Cell, Used for Treating Subject Having Disorder E.G. Hypertriglyceridemia, Non-Alcoholic Fatty Liver Disease, and Obesity That Would Benefit from Reduction in Apolipoprotein C3 Expression. Patents WO2021167841-A1, US2021292756-A1, US11162103-B2, 2021

[ref121] The Basics: In Vitro Transcription. https://www.thermofisher.com/us/en/home/references/ambion-tech-support/probe-labeling-systems/general-articles/the-basics-in-vitro-transcription.html (accessed 25 Nov 2021).

[ref122] Moderna. https://www.modernatx.com/about-us (accessed Nov 20, 2021).

[ref123] Biontech. https://biontech.de/ (accessed Nov 20, 2021).

[ref124] Curevac. https://www.curevac.com/en/ (accessed Nov 20, 2021).

[ref125] Stemirna Therapeutics. https://www.stemirna.com/en/about/index.aspx (accessed Nov 20, 2021).

[ref126] Cartesian Therapeutics. https://www.cartesiantherapeutics.com/rna-cell-therapy/ (accessed Nov 20, 2021).

[ref127] Alnylam https://www.alnylam.com/about-alnylam/ (accessed Nov 20, 2021).

[ref128] Dicerna https://dicerna.com/ (accessed Nov 20, 2021).

[ref129] siRNAomics https://sirnaomics.com/overview/ (accessed Nov 20, 2021).

[ref130] Arrowhead Pharmaceuticals. https://arrowheadpharma.com/about/ (accessed Nov 20, 2021).

[ref131] Silence Therapeutics. https://silence-therapeutics.com/about-us/default.aspx (accessed Nov 20, 2021).

[ref132] Ionis: A Force for Life. https://www.ionispharma.com/about/ (accessed 20 Niv 2021).

[ref133] Sarepta Therapeutics. https://www.sarepta.com/about-us (accessed Nov 20, 2021).

[ref134] Noxxon Pharma. https://www.noxxon.com/ (accessed Nov 20, 2021).

[ref135] Beam Therapeutics. https://beamtx.com/ (accessed Nov 20, 2021).

[ref136] Astrazeneca. https://www.astrazeneca.com/our-company.html (accessed Nov 20, 2021).

[ref137] Curevac Pipeline. https://www.curevac.com/en/pipeline/ (accessed Nov 20, 2021).

[ref138] Cardiovascular Diseases. https://www.who.int/health-topics/cardiovascular-diseases#tab=tab_1 (accessed Nov 20, 2021).

[ref139] Fda Approves Novartis Leqvio (Inclisiran), First-in-Class siRNA to Lower Cholesterol and Keep It Low with Two Doses a Year. https://www.novartis.com/news/media-releases/fda-approves-novartis-leqvio-inclisiran-first-class-sirna-lower-cholesterol-and-keep-it-low-two-doses-year (accessed Dec 27, 2021).

[ref140] Delivery Platforms. https://www.alnylam.com/our-science/sirna-delivery-platforms/ (accessed Nov 20, 2021).

[ref141] Study to Investigate Safety, Tolerability, Pk and Pd Response of Sln360 in Subjects with Elevated Lipoprotein(a). https://clinicaltrials.gov/ct2/show/NCT04606602?term=NCT04606602&draw=2&rank=1 (accessed Nov 20, 2021).

[ref142] Alnylam Pipeline. https://www.alnylam.com/alnylam-rnai-pipeline/ (accessed Nov 20, 2021).

[ref143] Zilebesiran (Aln-Agt), in Development for the Treatment of Hypertension. https://www.alnylam.com/wp-content/uploads/2021/06/2021-RNAi-Roundtables-Zilebesiran_FINAL.pdf (accessed Nov 20, 2021).

[ref144] Moderna’s Pipeline. https://www.modernatx.com/pipeline (accessed Nov 20, 2021).

[ref145] Anttila V.; Saraste A.; Knuuti J.; Jaakkola P.; Hedman M.; Svedlund S.; Lagerström-Fermér M.; Kjaer M.; Jeppsson A.; Gan L.-M. Synthetic mRNA Encoding Vegf-a in Patients Undergoing Coronary Artery Bypass Grafting: Design of a Phase 2a Clinical Trial. Mol. Ther. - Methods Clin. Dev. 2020, 18, 464–472. 10.1016/j.omtm.2020.05.030. 32728595 PMC7369517

[ref146] Taimeh Z.; Loughran J.; Birks E. J.; Bolli R. Vascular Endothelial Growth Factor in Heart Failure. Nat. Rev. Cardiol. 2013, 10, 519–530. 10.1038/nrcardio.2013.94. 23856679

[ref147] A Study to Evaluate ALN-AGT01 in Patients with Hypertension. https://clinicaltrials.gov/ct2/show/NCT03934307?term=NCT03934307&draw=2&rank=1 (accessed Nov 20, 2021).

[ref148] Azd8601 Study in Cabg Patients. https://clinicaltrials.gov/ct2/show/NCT03370887?term=NCT03370887&draw=2&rank=1 (accessed Nov 20, 2021).

[ref149] Saklayen M. G. The Global Epidemic of the Metabolic Syndrome. Curr. Hypertens. Rep. 2018, 20, 12 10.1007/s11906-018-0812-z. 29480368 PMC5866840

[ref150] Diabetes. https://www.who.int/health-topics/diabetes#tab=tab_1 (accessed Nov 20, 2021).

[ref151] The Ionis Antisense Pipeline. https://www.ionispharma.com/ionis-innovation/pipeline/ (accessed Nov 20, 2021).

[ref152] Morgan E. S.; Tai L. J.; Pham N. C.; Overman J. K.; Watts L. M.; Smith A.; Jung S. W.; Gajdošík M.; Krššák M.; Krebs M.; Geary R. S.; Baker B. F.; Bhanot S. Antisense Inhibition of Glucagon Receptor by Ionis-Gcgr(Rx) Improves Type 2 Diabetes without Increase in Hepatic Glycogen Content in Patients with Type 2 Diabetes on Stable Metformin Therapy. Diabetes Care 2019, 42, 585–593. 10.2337/dc18-1343. 30765435

[ref153] Paik J.; Duggan S. Volanesorsen: First Global Approval. Drugs 2019, 79, 1349–1354. 10.1007/s40265-019-01168-z. 31301033

[ref154] Witztum J. L.; Gaudet D.; Freedman S. D.; Alexander V. J.; Digenio A.; Williams K. R.; Yang Q.; Hughes S. G.; Geary R. S.; Arca M.; Stroes E. S. G.; Bergeron J.; Soran H.; Civeira F.; Hemphill L.; Tsimikas S.; Blom D. J.; O’Dea L.; Bruckert E. Volanesorsen and Triglyceride Levels in Familial Chylomicronemia Syndrome. N. Engl. J. Med. 2019, 381, 531–542. 10.1056/NEJMoa1715944. 31390500

[ref155] Strnad P.; McElvaney N. G.; Lomas D. A. Alpha(1)-Antitrypsin Deficiency. N Engl J. Med. 2020, 382, 1443–1455. 10.1056/NEJMra1910234. 32268028

[ref156] Wooddell C. I.; Blomenkamp K.; Peterson R. M.; Subbotin V. M.; Schwabe C.; Hamilton J.; Chu Q.; Christianson D. R.; Hegge J. O.; Kolbe J.; Hamilton H. L.; Branca-Afrazi M. F.; Given B. D.; Lewis D. L.; Gane E.; Kanner S. B.; Teckman J. H. Development of an RNAi Therapeutic for Alpha-1-Antitrypsin Liver Disease. JCI Insight 2020, 5, 135348 10.1172/jci.insight.135348. 32379724 PMC7406265

[ref157] Precise, Versatile Editing Platform. https://beamtx.com/our-portfolio/ (accessed Nov 20, 2021).

[ref158] Understanding the Human Genome. https://beamtx.com/our-science/ (accessed Nov 20, 2021).

[ref159] Methylmalonic Acidemia. https://medlineplus.gov/genetics/condition/methylmalonic-acidemia/ (accessed Nov 20, 2021).

[ref160] Moderna Announces First Patient Dosed in Phase 1/2 Study of mRNA-3705 for Methylmalonic Acidemia. https://investors.modernatx.com/news-releases/news-release-details/moderna-announces-first-patient-dosed-phase-12-study-mrna-3705 (accessed Nov 20, 2021).

[ref161] Safety, Tolerability and Efficacy of Isis-Gcgrrx in Type 2 Diabetes. https://clinicaltrials.gov/ct2/show/NCT01885260?term=NCT01885260&draw=2&rank=1 (accessed Nov 20, 2021).

[ref162] Study of Aro-Aat in Patients with Alpha-1 Antitrypsin Deficiency Associated Liver Disease (Aatd). https://clinicaltrials.gov/ct2/show/NCT03946449?term=NCT03946449&draw=2&rank=1 (accessed Nov 20, 2021).

[ref163] A Study to Assess Safety, Pharmacokinetics, and Pharmacodynamics of mRNA-3705 in Participants with Isolated Methylmalonic Acidemia. https://clinicaltrials.gov/ct2/show/NCT04899310?term=NCT04899310&draw=2&rank=1 (accessed Nov 20, 2021).

[ref164] Moon A. M.; Singal A. G.; Tapper E. B. Contemporary Epidemiology of Chronic Liver Disease and Cirrhosis. Clin Gastroenterol Hepatol 2020, 18, 2650–2666. 10.1016/j.cgh.2019.07.060. 31401364 PMC7007353

[ref165] Asrani S. K.; Devarbhavi H.; Eaton J.; Kamath P. S. Burden of Liver Diseases in the World. J. Hepatol 2019, 70, 151–171. 10.1016/j.jhep.2018.09.014. 30266282

[ref166] Acute Hepatic Porphyria (Ahp). https://liverfoundation.org/acute-hepatic-porphyria-ahp/#what-are-some-symptoms-of-ahp (accessed Nov 20, 2021).

[ref167] Alnylam Announces Approval of Givlaari (Givosiran) by the U.S. Food and Drug Administration (FDA). https://investors.alnylam.com/press-release?id=24281 (accessed Nov 20, 2021).

[ref168] A Study to Assess Safety, Tolerability, Pk and Pd of Azd2693 in Non-Alcoholic Steatohepatitis Patients. https://clinicaltrials.gov/ct2/show/NCT04483947?term=NCT04483947&draw=2&rank=1 (accessed Nov 20, 2021).

[ref169] BasuRay S.; Wang Y.; Smagris E.; Cohen J. C.; Hobbs H. H. Accumulation of Pnpla3 on Lipid Droplets Is the Basis of Associated Hepatic Steatosis. Proc. Natl. Acad. Sci. U. S. A. 2019, 116, 9521–9526. 10.1073/pnas.1901974116. 31019090 PMC6511016

[ref170] FDA Approves Givosiran for Acute Hepatic Porphyria. https://www.fda.gov/drugs/resources-information-approved-drugs/fda-approves-givosiran-acute-hepatic-porphyria (accessed Nov 20, 2021).

[ref171] Sung H.; Ferlay J.; Siegel R. L.; Laversanne M.; Soerjomataram I.; Jemal A.; Bray F. Global Cancer Statistics 2020: Globocan Estimates of Incidence and Mortality Worldwide for 36 Cancers in 185 Countries. CA: A Cancer Journal for Clinicians 2021, 71, 209–249. 10.3322/caac.21660. 33538338

[ref172] Noxxon ’S Pipeline. https://www.noxxon.com/index.php?option=com_content&view=article&id=15&Itemid=503 (accessed Nov 20, 2021).

[ref173] Nox-A12 https://www.noxxon.com/index.php?option=com_content&view=article&id=21&Itemid=478 (accessed Nov 20, 2021).

[ref174] Guo F.; Wang Y.; Liu J.; Mok S. C.; Xue F.; Zhang W. Cxcl12/Cxcr4: A Symbiotic Bridge Linking Cancer Cells and Their Stromal Neighbors in Oncogenic Communication Networks. Oncogene 2016, 35, 816–826. 10.1038/onc.2015.139. 25961926

[ref175] siRNAomics Pipeline. https://sirnaomics.com/pipeline/ (accessed Nov 20, 2021).

[ref176] siRNAomics Receives Fda Approval of Ind for Phase 1 Clinical Trial of Systemic RNAi Therapeutic Stp707 for Solid Tumor Treatment. https://sirnaomics.com/news/sirnaomics-receives-fda-approval-of-ind-for-phase-1-clinical-trial-of-systemic-rnai-therapeutic-stp707-for-solid-tumor-treatment/ (accessed Nov 20, 2021).

[ref177] Cartesian’s Pipeline. https://www.cartesiantherapeutics.com/clinical-trials/ (accessed Nov 20, 2021).

[ref178] What Is Multiple Myeloma? https://www.cancer.org/cancer/multiple-myeloma/about/what-is-multiple-myeloma.html (accessed Nov 20, 2021).

[ref179] Glioblastoma Treatment with Irradiation and Olaptesed Pegol (Nox-A12) in Mgmt Unmethylated Patients (Gloria). https://clinicaltrials.gov/ct2/show/NCT04121455?term=NCT04121455&draw=2&rank=1 (accessed Nov 20, 2021).

[ref180] Olaptesed with Pembrolizumab and Nanoliposomal Irinotecan or Gemcitabine/Nab-Paclitaxel in Mss Pancreatic Cancer (Optimus). https://clinicaltrials.gov/ct2/show/NCT04901741?term=NCT04901741&draw=2&rank=1 (accessed Nov 20, 2021).

[ref181] Ph. 1, Evaluation of Safety, Tolerability, PK, Anti-Tumor Activity of STP707 IV in Subjects with Solid Tumors. https://clinicaltrials.gov/ct2/show/NCT05037149?term=NCT05037149&draw=2&rank=1 (accessed Nov 20, 2021).

[ref182] Descartes-11 in Multiple Myeloma. https://clinicaltrials.gov/ct2/show/NCT03994705?term=NCT03994705&draw=2&rank=1 (accessed Nov 20, 2021).

[ref183] Who Coronavirus (COVID-19) Dashboard. https://covid19.who.int/ (accessed Feb 23, 2022).

[ref184] Pfizer-Biontech COVID-19 Vaccine Comirnaty® Receives Full U.S. FDA Approval for Individuals 16 Years and Older. https://www.pfizer.com/news/press-release/press-release-detail/pfizer-biontech-covid-19-vaccine-comirnatyr-receives-full (accessed Nov 20, 2021).

[ref185] Moderna COVID-19 Vaccine. https://www.fda.gov/emergency-preparedness-and-response/coronavirus-disease-2019-covid-19/moderna-covid-19-vaccine (accessed Nov 20, 2021).

[ref186] Coronavirus (COVID-19) Vaccinations. https://ourworldindata.org/covid-vaccinations?country=OWID_WRL (accessed Feb 23, 2022).

[ref187] Ab-729 (GalNAc-RNAi). http://www.arbutusbio.com/portfolio/ab-729-galnac-rnai.php (accessed Nov 20, 2021).

[ref188] Kapoor R.; Kottilil S. Strategies to Eliminate Hbv Infection. Future Virol 2014, 9, 565–585. 10.2217/fvl.14.36. 25309617 PMC4190097

[ref189] Moderna Announces First Participant Dosed in Phase 1/2 Study of Its Quadrivalent Seasonal Flu mRNA Vaccine. https://investors.modernatx.com/news-releases/news-release-details/moderna-announces-first-participant-dosed-phase-12-study-its (accessed Nov 20, 2021).

[ref190] Moderna Receives Fda Fast Track Designation for Respiratory Syncytial Virus (RSV) Vaccine (mRNA-1345). https://investors.modernatx.com/news-releases/news-release-details/moderna-receives-fda-fast-track-designation-respiratory (accessed Nov 20, 2021).

[ref191] Xu L.; Ma Z.; Li Y.; Pang Z.; Xiao S. Antibody Dependent Enhancement: Unavoidable Problems in Vaccine Development. Adv. Immunol. 2021, 151, 99–133. 10.1016/bs.ai.2021.08.003. 34656289 PMC8438590

[ref192] Safety, Tolerability, and Pharmacokinetics of Ab-836 in Healthy Subjects and Subjects with Chronic Hbv Infection. https://clinicaltrials.gov/ct2/show/NCT04775797?term=NCT04775797&draw=2&rank=1 (accessed Nov 20, 2021).

[ref193] A Study of mRNA-1010 Seasonal Influenza Vaccine in Healthy Adults. https://clinicaltrials.gov/ct2/show/NCT04956575?term=NCT04956575&draw=2&rank=1 (accessed Nov 20, 2021).

[ref194] A Dose Escalation Study to Evaluate Safety, Reactogenicity, and Immunogenicity of mRNA-1345 in Healthy Adults and in Children Who Are Respiratory Syncytial Virus (Rsv)-Seropositive. https://clinicaltrials.gov/ct2/show/NCT04528719?term=NCT04528719&draw=2&rank=1 (accessed Nov 20, 2021).

[ref195] Neuromuscular Disorders. https://www.cedars-sinai.org/health-library/diseases-and-conditions/n/neuromuscular-disorders.html (accessed Nov 20, 2021).

[ref196] Neurological Disorders. https://dphhs.mt.gov/schoolhealth/chronichealth/neurologicaldisorders (accessed Nov 20, 2021).

[ref197] Fda Grants Accelerated Approval to First Drug for Duchenne Muscular Dystrophy. https://www.fda.gov/news-events/press-announcements/fda-grants-accelerated-approval-first-drug-duchenne-muscular-dystrophy (accessed Nov 20, 2021).

[ref198] Fda Grants Accelerated Approval to First Targeted Treatment for Rare Duchenne Muscular Dystrophy Mutation. https://www.fda.gov/news-events/press-announcements/fda-grants-accelerated-approval-first-targeted-treatment-rare-duchenne-muscular-dystrophy-mutation (accessed Nov 20, 2021).

[ref199] Fda Approves Targeted Treatment for Rare Duchenne Muscular Dystrophy Mutation. https://www.fda.gov/news-events/press-announcements/fda-approves-targeted-treatment-rare-duchenne-muscular-dystrophy-mutation (accessed Feb 23, 2022).

[ref200] Ottesen E. W. Iss-N1Makes the First Fda-Approved Drug for Spinal Muscular Atrophy. Transl. Neurosci. 2017, 8, 1–6. 10.1515/tnsci-2017-0001. 28400976 PMC5382937

[ref201] Gales L. Tegsedi (Inotersen): An Antisense Oligonucleotide Approved for the Treatment of Adult Patients with Hereditary Transthyretin Amyloidosis. Pharmaceuticals 2019, 12, 78 10.3390/ph12020078. PMC6631675 31117178

[ref202] Gomez-Aguado I.; Rodriguez-Castejon J.; Vicente-Pascual M.; Rodriguez-Gascon A.; Solinis M. A.; del Pozo-Rodriguez A. Nanomedicines to Deliver mRNA: State of the Art and Future Perspectives. Nanomaterials 2020, 10, 364 10.3390/nano10020364. PMC7075285 32093140

[ref203] Building an Industry-Leading Genetic Medicine Pipeline. https://www.sarepta.com/products-pipeline/pipeline (accessed Nov 20, 2021).

[ref204] What Is Als? https://www.als.org/understanding-als/what-is-als (accessed Nov 20, 2021).

[ref205] Delivering on the RNA Revolution. https://www.aviditybiosciences.com/pipeline/pipeline-overview/ (accessed Nov 20, 2021).

[ref206] Holt I.; Jacquemin V.; Fardaei M.; Sewry C. A.; Butler-Browne G. S.; Furling D.; Brook J. D.; Morris G. E. Muscleblind-Like Proteins: Similarities and Differences in Normal and Myotonic Dystrophy Muscle. Am. J. Pathol. 2009, 174, 216–227. 10.2353/ajpath.2009.080520. 19095965 PMC2631334

[ref207] Akinc A.; Maier M. A.; Manoharan M.; Fitzgerald K.; Jayaraman M.; Barros S.; Ansell S.; Du X. Y.; Hope M. J.; Madden T. D.; Mui B. L.; Semple S. C.; Tam Y. K.; Ciufolini M.; Witzigmann D.; Kulkarni J. A.; van der Meel R.; Cullis P. R. The Onpattro Story and the Clinical Translation of Nanomedicines Containing Nucleic Acid-Based Drugs. Nat. Nanotechnol. 2019, 14, 1084–1087. 10.1038/s41565-019-0591-y. 31802031

[ref208] A Study of Biib067 When Initiated in Clinically Presymptomatic Adults with a Confirmed Superoxide Dismutase 1 Mutation (Atlas). https://clinicaltrials.gov/ct2/show/NCT04856982?term=NCT04856982&draw (accessed Nov 20, 2021).

[ref209] Study of Aoc 1001 in Adult Myotonic Dystrophy Type 1 (Dm1) Patients (Marina). https://clinicaltrials.gov/ct2/show/NCT05027269?term=NCT05027269&draw=2&rank=1 (accessed Nov 20, 2021).

[ref210] Blindness and Vision Impairment. https://www.who.int/news-room/fact-sheets/detail/blindness-and-visual-impairment (accessed Nov 20, 2021).

[ref211] Wong W. L.; Su X.; Li X.; Cheung C. M.; Klein R.; Cheng C. Y.; Wong T. Y. Global Prevalence of Age-Related Macular Degeneration and Disease Burden Projection for 2020 and 2040: A Systematic Review and Meta-Analysis. Lancet Glob. Health 2014, 2, e106–16. 10.1016/S2214-109X(13)70145-1. 25104651

[ref212] Nagpal M.; Nagpal K.; Nagpal P. N. A Comparative Debate on the Various Anti-Vascular Endothelial Growth Factor Drugs: Pegaptanib Sodium (Macugen), Ranibizumab (Lucentis) and Bevacizumab (Avastin). Indian J. Ophthalmol. 2007, 55, 437–439. 10.4103/0301-4738.36478. 17951900 PMC2635991

[ref213] Adamis A. P.; Altaweel M.; Bressler N. M.; Cunningham E. T. Jr.; Davis M. D.; Goldbaum M.; Gonzales C.; Guyer D. R.; Barrett K.; Patel M. Changes in Retinal Neovascularization after Pegaptanib (Macugen) Therapy in Diabetic Individuals. Ophthalmology 2006, 113, 23–28. 10.1016/j.ophtha.2005.10.012. 16343627

[ref214] Jaffe G. J.; Westby K.; Csaky K. G.; Monés J.; Pearlman J. A.; Patel S. S.; Joondeph B. C.; Randolph J.; Masonson H.; Rezaei K. A. C5 Inhibitor Avacincaptad Pegol for Geographic Atrophy Due to Age-Related Macular Degeneration: A Randomized Pivotal Phase 2/3 Trial. Ophthalmology 2021, 128, 576–586. 10.1016/j.ophtha.2020.08.027. 32882310

[ref215] A Phase 3 Safety and Efficacy Study of Intravitreal Administration of Zimura (Complement C5 Inhibitor). https://clinicaltrials.gov/ct2/show/NCT04435366?term=NCT04435366&draw=2&rank=1 (accessed Nov 20, 2021).

[ref216] Study with Qr-504a to Evaluate Safety, Tolerability & Corneal Endothelium Molecular Biomarker(S) in Subjects with Fecd3 (Fuchs Focus). https://clinicaltrials.gov/ct2/show/NCT05052554 (accessed Nov 20, 2021).

[ref217] Qr-504a for Fuchs Endothelial Corneal Dystrophy. https://www.proqr.com/qr-504a-for-fuchs-endothelial-corneal-dystrophy (accessed Nov 20, 2021).

[ref218] 850 Million People Worldwide Have Kidney Disease. https://www.webmd.com/kidney-stones/news/20180705/850-million-people-worldwide-have-kidney-disease (accessed Nov 20, 2021).

[ref219] Scott L. J.; Keam S. J. Lumasiran: First Approval. Drugs 2021, 81, 277–282. 10.1007/s40265-020-01463-0. 33405070

[ref220] Garrelfs S. F.; Frishberg Y.; Hulton S. A.; Koren M. J.; O’Riordan W. D.; Cochat P.; Deschênes G.; Shasha-Lavsky H.; Saland J. M.; Van’t Hoff W. G.; Fuster D. G.; Magen D.; Moochhala S. H.; Schalk G.; Simkova E.; Groothoff J. W.; Sas D. J.; Meliambro K. A.; Lu J.; Sweetser M. T.; Garg P. P.; Vaishnaw A. K.; Gansner J. M.; McGregor T. L.; Lieske J. C. Lumasiran, an RNAi Therapeutic for Primary Hyperoxaluria Type 1. N. Engl. J. Med. 2021, 384, 1216–1226. 10.1056/NEJMoa2021712. 33789010

[ref221] Lee E. C.; Valencia T.; Allerson C.; Schairer A.; Flaten A.; Yheskel M.; Kersjes K.; Li J.; Gatto S.; Takhar M.; Lockton S.; Pavlicek A.; Kim M.; Chu T.; Soriano R.; Davis S.; Androsavich J. R.; Sarwary S.; Owen T.; Kaplan J.; Liu K.; Jang G.; Neben S.; Bentley P.; Wright T.; Patel V. Discovery and Preclinical Evaluation of Anti-Mir-17 Oligonucleotide Rgls4326 for the Treatment of Polycystic Kidney Disease. Nat. Commun. 2019, 10, 4148 10.1038/s41467-019-11918-y. 31515477 PMC6742637

[ref222] Schairer A.; Valencia T.; Lockton S.; Gatto S.; Kim M.; Wallace D.; Lee E. Efficacy of RGLS4326 in Human Primary 3D-Cyst Cultures Derived from Autosomal Dominant Polycystic Kidney Disease (ADPKD) Donors http://regulusrx.com/wp-content/uploads/2018/11/ASN-2018-RGLS4326-Poster-3021589-FINAL.pdf (accessed Nov 20, 2021).

[ref223] Ji F. P.; Wen L.; Zhang Y. P.; Liu E. P.; Wen J. G., Serum Complement Factor B Is Associated with Disease Activity and Progression of Idiopathic Membranous Nephropathy Concomitant with Iga Nephropathy. Int. Urol. Nephrol. 2021. 10.1007/s11255-021-02997-2 34585312

[ref224] A Study to Evaluate Lumasiran in Patients with Advanced Primary Hyperoxaluria Type 1 (Illuminate-C). https://clinicaltrials.gov/ct2/show/NCT04152200?term=NCT04152200&draw=2&rank=1 (accessed Nov 20, 2021).

[ref225] A Study of Rgls4326 in Patients with Autosomal Dominant Polycystic Kidney Disease. https://clinicaltrials.gov/ct2/show/NCT04536688?term=NCT04536688&draw=2&rank=1 (accessed Nov 20, 2021).

[ref226] A Study to Evaluate the Effectiveness and Safety of IONIS-FB-LRx, an Antisense Inhibitor of Complement Factor B, in Adult Participants with Primary IgA Nephropathy. https://clinicaltrials.gov/ct2/show/NCT04014335?term=NCT04014335&draw=2&rank=1 (accessed Nov 20, 2021).

[ref227] The Global Impact of Respiratory Disease. https://www.who.int/gard/publications/The_Global_Impact_of_Respiratory_Disease.pdf (accessed Nov 20, 2021).

[ref228] Cartesian Therapeutics Initiates Clinical Trial of First RNA-Engineered Cell Therapy for Acute Respiratory Distress Syndrome and COVID-19 https://www.prnewswire.com/news-releases/cartesian-therapeutics-initiates-clinical-trial-of-first-rna-engineered-cell-therapy-for-acute-respiratory-distress-syndrome-and-covid-19-301121921.html (accessed Nov 20, 2021).

[ref229] Study of Descartes-30 in Acute Respiratory Distress Syndrome. https://clinicaltrials.gov/ct2/show/NCT04524962?term=NCT04524962&draw=2&rank=1 (accessed Nov 20, 2021).

[ref230] Ratjen F.; Bell S. C.; Rowe S. M.; Goss C. H.; Quittner A. L.; Bush A. Cystic Fibrosis. Nat. Rev. Dis. Primers 2015, 1, 15010–15010. 10.1038/nrdp.2015.10. 27189798 PMC7041544

[ref231] Translate Bio Pipeline - Present and Future Focus. https://translate.bio/pipeline/ (accessed Nov 20, 2021).

[ref232] Chow M. Y. T.; Qiu Y.; Lam J. K. W. Inhaled RNA Therapy: From Promise to Reality. Trends Pharmacol. Sci. 2020, 41, 715–729. 10.1016/j.tips.2020.08.002. 32893004 PMC7471058

[ref233] Translate Bio Announces Results from Second Interim Data Analysis from Ongoing Phase 1/2 Clinical Trial of Mrt5005 in Patients with Cystic Fibrosis (CF). https://investors.translate.bio/news-releases/news-release-details/translate-bio-announces-results-second-interim-data-analysis (accessed Nov 20, 2021).

[ref234] Arrowhead’s Pipeline - Novel Drugs Totreat Intractable Diseases. https://arrowheadpharma.com/pipeline/ (accessed Nov 20, 2021).

[ref235] Arrowhead Pauses Aro-Enac Phase 1/2 Clinical Study. https://ir.arrowheadpharma.com/news-releases/news-release-details/arrowhead-pauses-aro-enac-phase-12-clinical-study (accessed Nov 20, 2021).

[ref236] Study to Evaluate the Safety & Tolerability of Mrt5005 Administered by Nebulization in Adults with Cystic Fibrosis (Restore-Cf). https://clinicaltrials.gov/ct2/show/NCT03375047?term=NCT03375047&draw=2&rank=1 (accessed Nov 20, 2021).

[ref237] Study of Aro-Enac in Healthy Volunteers and in Patients with Cystic Fibrosis. https://clinicaltrials.gov/ct2/show/NCT04375514?term=NCT04375514&draw=2&rank=1 (accessed Nov 20, 2021).

[ref238] Salinas Cisneros G.; Thein S. L. Recent Advances in the Treatment of Sickle Cell Disease. Front. Physiol. 2020, 11, 435 10.3389/fphys.2020.00435. 32508672 PMC7252227

[ref239] Galanello R.; Origa R. Beta-Thalassemia. Orphanet J. Rare Dis. 2010, 5, 11 10.1186/1750-1172-5-11. 20492708 PMC2893117

[ref240] Beam Therapeutics Provides Business and Pipeline Updates and Reports Third Quarter 2021 Financial Results. https://investors.beamtx.com/node/7596 (accessed Nov 20, 2021).

[ref241] Silence Therapeutics Pipeline - Our mRNAi Gold Pipeline Targets. https://silence-therapeutics.com/our-pipeline/default.aspx (accessed Nov 20, 2021).

[ref242] SLN-124. https://thalassaemia.org.cy/clinical-trial-updates/sln-124/ (accessed Nov 20, 2021).

[ref243] A Study Investigate the Safety, Tolerability, Pharmacokinetic, and Pharmacodynamic Response of Sln124 in Adults with Alpha/Beta-Thalassaemia and Very Low- and Low-Risk Myelodysplastic Syndrome. https://clinicaltrials.gov/ct2/show/NCT04718844?term=NCT04718844&draw=2&rank=1 (accessed Nov 20, 2021).

[ref244] A Study for Safety and Efficacy Evaluation of Various Doses of Stp705 in Reducing Keloid Recurrence. https://clinicaltrials.gov/ct2/show/NCT04844840?term=NCT04844840&draw=2&rank=1 (accessed Nov 20, 2021).

[ref245] siRNAomics Doses First Patient in Phase 2 Study of Stp705 for Keloid Scar Prevention. https://sirnaomics.com/news/sirnaomics-doses-first-patient-in-phase-2-study-of-stp705-for-keloid-scar-prevention/ (accessed Nov 20, 2021).

[ref246] Janeway C. A. Jr.; Travers P; Walport M.; et al. Autoimmune Responses Are Directed against Self Antigens. Immunobiology: The Immune System in Health and Disease, 5th ed.; Garland Science, New York, 2001.

[ref247] Moderna Builds on Clinical Validation of Systemic Delivery with Two Additional Development Candidates in New Autoimmune Therapeutic Area. https://investors.modernatx.com/news-releases/news-release-details/moderna-builds-clinical-validation-systemic-delivery-two (accessed Nov 20, 2021).

[ref248] Global Alcohol Action Plan 2022–2030 to Strengthen Implementation of the Global Strategy to Reduce the Harmful Use of Alcohol. https://cdn.who.int/media/docs/default-source/alcohol/action-plan-on-alcohol_first-draft-final_formatted.pdf?sfvrsn=b690edb0_1&download=true (accessed Nov 20, 2021).

[ref249] Alcohol Use Disorder (AUD). https://dicerna.com/patients/alcohol-use-disorder-aud/ (accessed Nov 20, 2021).

[ref250] A New and Unique Approach to Potentially Improving Treatment Outcomes for People with Alcohol Use Disorder (Aud). https://dicerna.com/pipeline/dcr-aud/ (accessed Nov 20, 2021).

[ref251] A Study to Evaluate the Safety, Tolerability, Pharmacokinetics, and Pharmacodynamics of mRNA-6231 in Healthy Adults. https://clinicaltrials.gov/ct2/show/NCT04916431?term=NCT04916431&draw=2&rank=1 (accessed Nov 20, 2021).

[ref252] Study of DCR-AUD in Healthy Volunteers. https://clinicaltrials.gov/ct2/show/NCT05021640?term=NCT05021640&draw=2&rank=1 (accessed Nov 20, 2021).

[ref253] Lehninger A. L. Biochemistry: The Molecular Basis of Cell Structure and Function, 2nd ed.; Worth Publishers: New York, NY, 1975.

[ref254] McIninch J. D.; Keating M.; Schlegel M. K.; Castoreno A.; Jadhav V. R.; Kaittanis C.; Castellanos-Rizaldos E.; Pandya B. A. Compositions and Methods for Silencing VEGF-A Expression. Patent WO/2021/163066, 2021.

[ref255] Parr C. J. C.; Wada S.; Kotake K.; Kameda S.; Matsuura S.; Sakashita S.; Park S.; Sugiyama H.; Kuang Y.; Saito H. N1-Methylpseudouridine Substitution Enhances the Performance of Synthetic mRNA Switches in Cells. Nucleic Acids Res. 2020, 48, e35–e35. 10.1093/nar/gkaa070. 32090264 PMC7102939

[ref256] Shatkin A. J. Capping of Eucaryotic mRNAs. Cell 1976, 9, 645–653. 10.1016/0092-8674(76)90128-8. 1017010

[ref257] Chan J. H.; Lim S.; Wong W. F. Antisense Oligonucleotides: From Design to Therapeutic Application. Clin. Exp. Pharmacol. Physiol. 2006, 33, 533–540. 10.1111/j.1440-1681.2006.04403.x. 16700890

[ref258] Phosphorothioate Oligonucleotides. https://www.sigmaaldrich.com/US/en/technical-documents/technical-article/genomics/gene-expression-and-silencing/phosphorothioates (accessed Nov 20, 2021).

[ref259] Putney S. D.; Benkovic S. J.; Schimmel P. R. A DNA Fragment with an Alpha-Phosphorothioate Nucleotide at One End Is Asymmetrically Blocked from Digestion by Exonuclease Iii and Can Be Replicated in Vivo. Proc. Natl. Acad. Sci. U. S. A. 1981, 78, 7350–7354. 10.1073/pnas.78.12.7350. 6278470 PMC349264

[ref260] Cantley W.; McIninch J. D.; Castoreno A.; Kaittanis C.; Schlegel M. K. Compositions and Methods for Silencing Scn9a Expression. Patent WO/2021/207189, 2021.

[ref261] Nielsen P. E.; Egholm M. An Introduction to Peptide Nucleic Acid. Curr. Issues Mol. Biol. 1999, 1, 89–104. 11475704

[ref262] Blocking Groups and Annotations for Registry Sequences. https://www.cas.org/sites/default/files/documents/blocking.pdf (accessed Jan 1, 2022).

[ref263] Chao Y.; Li H.-B.; Zhou J. Multiple Functions of RNA Methylation in T Cells: A Review. Front. Immunol. 2021, 12, 627455 10.3389/fimmu.2021.627455. 33912158 PMC8071866

[ref264] Perry R. P.; Kelley D. E. Existence of Methylated Messenger RNA in Mouse L Cells. Cell 1974, 1, 37–42. 10.1016/0092-8674(74)90153-6.

[ref265] Summerton J. Morpholino Antisense Oligomers: The Case for an RNAse H-Independent Structural Type. Biochim. Biophys. Acta 1999, 1489, 141–158. 10.1016/S0167-4781(99)00150-5. 10807004

[ref266] Fitzgerald K.; Tremblay F.; McIninch J. D. G Protein-Coupled Receptor 146 (Gpr146) iRNA Compositions and Methods of Use Thereof. Patent WO/2021/174056, 2021.

[ref267] Springer A. D. GalNAc-siRNA Conjugates: Leading the Way for Delivery of RNAi Therapeutics. Nucleic Acid Ther. 2018, 28, 109–118. 10.1089/nat.2018.0736. 29792572 PMC5994659

[ref268] Fitzgerald K.; Tremblay F.; McIninch J. D. G Protein-Coupled Receptor 146 (Gpr146) iRNA Compositions and Methods of Use Thereof. Patent WO/2021/174056, 2021.

[ref269] Pubchem Substance Record for Sid 135267507, Aiw6036fas. https://pubchem.ncbi.nlm.nih.gov/substance/135267507 (accessed Dec 9, 2021).

[ref270] Pegaptanib. https://en.wikipedia.org/wiki/Pegaptanib#/media/File:Pegaptanib_sodium_skeletal.svg (accessed Dec 8, 2021).

[ref271] Vieira Araujo Soares Da Silva P. M. siRNA Molecules, Methods of Production and Uses Thereof. U.S. Patent US20210332364, 2021.

[ref272] Kanasty R.; Dorkin J. R.; Vegas A.; Anderson D. Delivery Materials for siRNA Therapeutics. Nat. Mater. 2013, 12, 967–977. 10.1038/nmat3765. 24150415

[ref273] CASData. https://www.cas.org/cas-data (accessed Nov 25, 2021).

[ref274] Yang N. Nonviral Gene Delivery System. Int. J. Pharmaceutical Investigation 2012, 2, 97–98. 10.4103/2230-973X.104388. PMC3555013 23373000

[ref275] Koynova R.; Tenchov B. Cationic Lipids: Molecular Structure/Transfection Activity Relationships and Interactions with Biomembranes. In Nucleic Acid Transfection; Bielke W., Erbacher C., Eds.; Springer-Verlag: Berlin, Heidelberg, 2010; Vol. 296, pp 51–93. 10.1007/128_2010_67 21504100

[ref276] Tenchov R.; Bird R.; Curtze A. E.; Zhou Q. Lipid Nanoparticles—from Liposomes to mRNA Vaccine Delivery, a Landscape of Research Diversity and Advancement. ACS Nano 2021, 15, 16982–17015. 10.1021/acsnano.1c04996. 34181394

[ref277] Mitchell M. J.; Billingsley M. M.; Haley R. M.; Wechsler M. E.; Peppas N. A.; Langer R. Engineering Precision Nanoparticles for Drug Delivery. Nat. Rev. Drug Disc. 2021, 20, 101–124. 10.1038/s41573-020-0090-8. PMC7717100 33277608

[ref278] Allison A. C.; Gregoriadis G. Liposomes as Immunological Adjuvants. Nature 1974, 252, 252–252. 10.1038/252252a0. 4424229

[ref279] Gregoriadis G. Liposomes and mRNA: Two Technologies Together Create a COVID-19 Vaccine. Med. Drug Disc. 2021, 12, 100104 10.1016/j.medidd.2021.100104.

[ref280] Attia M. A.; Essa E. A.; Elebyary T. T.; Faheem A. M.; Elkordy A. A. Brief on Recent Application of Liposomal Vaccines for Lower Respiratory Tract Viral Infections: From Influenza to COVID-19 Vaccines. Pharmaceuticals (Basel) 2021, 14, 1173 10.3390/ph14111173. 34832955 PMC8619292

[ref281] Rosales-Mendoza S.; Wong-Arce A.; de Lourdes; Betancourt-Mendiola M. RNA-Based Vaccines against Sars-Cov-2. In Biomedical Innovations to Combat COVID-19; Rosales-Mendoza S., Comas-Garcia M., Gonzalez-Ortega O., Eds.; Academic Press, 2022; Chap. 8, pp 129–152.

[ref282] Hajj K. A.; Ball R. L.; Deluty S. B.; Singh S. R.; Strelkova D.; Knapp C. M.; Whitehead K. A. Branched-Tail Lipid Nanoparticles Potently Deliver mRNA *in Vivo* Due to Enhanced Ionization at Endosomal pH. Small 2019, 15, e1805097 10.1002/smll.201805097. 30637934

[ref283] Heyes J. A.; Niculescu-Duvaz D.; Cooper R. G.; Springer C. J. Synthesis of Novel Cationic Lipids: Effect of Structural Modification on the Efficiency of Gene Transfer. J. Med. Chem. 2002, 45, 99–114. 10.1021/jm010918g. 11754582

[ref284] Karmali P. P.; Chaudhuri A. Cationic Liposomes as Non-Viral Carriers of Gene Medicines: Resolved Issues, Open Questions, and Future Promises. Med. Res. Rev. 2007, 27, 696–722. 10.1002/med.20090. 17022036

[ref285] Karmali P. P.; Kumar V. V.; Chaudhuri A. Design, Syntheses and in Vitro Gene Delivery Efficacies of Novel Mono-, Di- and Trilysinated Cationic Lipids: A Structure-Activity Investigation. J. Med. Chem. 2004, 47, 2123–2132. 10.1021/jm030541+. 15056009

[ref286] Behr J P; Demeneix B; Loeffler J P; Perez-Mutul J Efficient Gene-Transfer into Mammalian Primary Endocrine-Cells with Lipopolyamine-Coated DNA. Proc. Natl. Acad. Sci. U. S. A. 1989, 86, 6982–6986. 10.1073/pnas.86.18.6982. 2780554 PMC297976

[ref287] Ferrari M. E.; Nguyen C. M.; Zelphati O.; Tsai Y. L.; Felgner P. L. Analytical Methods for the Characterization of Cationic Lipid Nucleic Acid Complexes. Hum. Gene Ther. 1998, 9, 341–351. 10.1089/hum.1998.9.3-341. 9508052

[ref288] de Lima M. C. P.; Neves S.; Filipe A.; Duzgunes N.; Simoes S. Cationic Liposomes for Gene Delivery: From Biophysics to Biological Applications. Curr. Med. Chem. 2003, 10, 1221–1231. 10.2174/0929867033457430. 12678796

[ref289] Wheeler C. J.; Felgner P. L.; Tsai Y. J.; Marshall J.; Sukhu L.; Doh S. G.; Hartikka J.; Nietupski J.; Manthorpe M.; Nichols M.; Plewe M.; Liang X. W.; Norman J.; Smith A.; Cheng S. H. A Novel Cationic Lipid Greatly Enhances Plasmid DNA Delivery and Expression in Mouse Lung. Proc. Natl. Acad. Sci. U. S. A. 1996, 93, 11454–11459. 10.1073/pnas.93.21.11454. 8876156 PMC38078

[ref290] Li Y.; Tenchov R.; Smoot J.; Liu C.; Watkins S.; Zhou Q. A Comprehensive Review of the Global Efforts on COVID-19 Vaccine Development. ACS Central Sci. 2021, 7, 512–533. 10.1021/acscentsci.1c00120. PMC8029445 34056083

[ref291] Tarahovsky Y. S.; Arsenault A. L.; MacDonald R. C.; McIntosh T. J.; Epand R. M. Electrostatic Control of Phospholipid Polymorphism. Biophys. J. 2000, 79, 3193–3200. 10.1016/S0006-3495(00)76552-0. 11106623 PMC1301194

[ref292] Koynova R.; Wang L.; MacDonald R. C. An Intracellular Lamellar - Nonlamellar Phase Transition Rationalizes the Superior Performance of Some Cationic Lipid Transfection Agents. Proc. Natl. Acad. Sci. U. S. A. 2006, 103, 14373–14378. 10.1073/pnas.0603085103. 16983097 PMC1599970

[ref293] Kedmi R.; Ben-Arie N.; Peer D. The Systemic Toxicity of Positively Charged Lipid Nanoparticles and the Role of Toll-Like Receptor 4 in Immune Activation. Biomaterials 2010, 31, 6867–6875. 10.1016/j.biomaterials.2010.05.027. 20541799

[ref294] Kalluri R.; LeBleu V. S. The Biology, Function, and Biomedical Applications of Exosomes. Science 2020, 367, aau6977 10.1126/science.aau6977. PMC7717626 32029601

[ref295] Edgar J. R. Q&A: What Are Exosomes, Exactly?. BMC Biol. 2016, 14, 46 10.1186/s12915-016-0268-z. 27296830 PMC4906597

[ref296] Zhang Y.; Liu Y.; Liu H.; Tang W. H. Exosomes: Biogenesis, Biologic Function and Clinical Potential. Cell Biosci. 2019, 9, 19 10.1186/s13578-019-0282-2. 30815248 PMC6377728

[ref297] Alvarez-Erviti L.; Seow Y.; Yin H.; Betts C.; Lakhal S.; Wood M. J. A. Delivery of siRNA to the Mouse Brain by Systemic Injection of Targeted Exosomes. Nat. Biotechnol. 2011, 29, 341–345. 10.1038/nbt.1807. 21423189

[ref298] Shtam T. A.; Kovalev R. A.; Varfolomeeva E. Y.; Makarov E. M.; Kil Y. V.; Filatov M. V. Exosomes Are Natural Carriers of Exogenous siRNA to Human Cells in Vitro. Cell Commun. Signal. 2013, 11, 88 10.1186/1478-811X-11-88. 24245560 PMC3895799

[ref299] Lamichhane T. N.; Raiker R. S.; Jay S. M. Exogenous DNA Loading into Extracellular Vesicles Via Electroporation Is Size-Dependent and Enables Limited Gene Delivery. Mol. Pharmaceutics 2015, 12, 3650–3657. 10.1021/acs.molpharmaceut.5b00364. PMC4826735 26376343

[ref300] Zhou X.; Brown B. A.; Siegel A. P.; El Masry M. S.; Zeng X.; Song W.; Das A.; Khandelwal P.; Clark A.; Singh K.; Guda P. R.; Gorain M.; Timsina L.; Xuan Y.; Jacobson S. C.; Novotny M. V.; Roy S.; Agarwal M.; Lee R. J.; Sen C. K.; Clemmer D. E.; Ghatak S. Exosome-Mediated Crosstalk between Keratinocytes and Macrophages in Cutaneous Wound Healing. ACS Nano 2020, 14, 12732–12748. 10.1021/acsnano.0c03064. 32931251 PMC7970718

[ref301] Subra C.; Laulagnier K.; Perret B.; Record M. Exosome Lipidomics Unravels Lipid Sorting at the Level of Multivesicular Bodies. Biochimie 2007, 89, 205–212. 10.1016/j.biochi.2006.10.014. 17157973

[ref302] Ha D.; Yang N.; Nadithe V. Exosomes as Therapeutic Drug Carriers and Delivery Vehicles across Biological Membranes: Current Perspectives and Future Challenges. Acta Pharm. Sin B 2016, 6, 287–296. 10.1016/j.apsb.2016.02.001. 27471669 PMC4951582

[ref303] Butreddy A.; Kommineni N.; Dudhipala N. Exosomes as Naturally Occurring Vehicles for Delivery of Biopharmaceuticals: Insights from Drug Delivery to Clinical Perspectives. Nanomaterials (Basel) 2021, 11, 1481 10.3390/nano11061481. 34204903 PMC8229362

[ref304] Zhang Y.; Sun C.; Wang C.; Jankovic K. E.; Dong Y. Lipids and Lipid Derivatives for RNA Delivery. Chem. Rev. 2021, 121, 12181–12277. 10.1021/acs.chemrev.1c00244. 34279087 PMC10088400

[ref305] Wang C.; Zhang Y.; Dong Y. Lipid Nanoparticle-mRNA Formulations for Therapeutic Applications. Acc. Chem. Res. 2021, 54, 4283–4293. 10.1021/acs.accounts.1c00550. 34793124 PMC10068911

[ref306] Shi J.; Kundrat L.; Pishesha N.; Bilate A.; Theile C.; Maruyama T.; Dougan S. K.; Ploegh H. L.; Lodish H. F. Engineered Red Blood Cells as Carriers for Systemic Delivery of a Wide Array of Functional Probes. Proc. Natl. Acad. Sci. U. S. A. 2014, 111, 10131–10136. 10.1073/pnas.1409861111. 24982154 PMC4104923

[ref307] Li S.; Feng S.; Ding L.; Liu Y.; Zhu Q.; Qian Z.; Gu Y. Nanomedicine Engulfed by Macrophages for Targeted Tumor Therapy. Int. J. Nanomed. 2016, 11, 4107–4124. 10.2147/IJN.S110146. PMC5003564 27601898

[ref308] Kumar R.; Santa Chalarca C. F.; Bockman M. R.; Bruggen C. V.; Grimme C. J.; Dalal R. J.; Hanson M. G.; Hexum J. K.; Reineke T. M. Polymeric Delivery of Therapeutic Nucleic Acids. Chem. Rev. 2021, 121, 11527–11652. 10.1021/acs.chemrev.0c00997. 33939409

[ref309] Zhu L.; Mahato R. I. Lipid and Polymeric Carrier-Mediated Nucleic Acid Delivery. Expert Opin. Drug Deliv. 2010, 7, 1209–1226. 10.1517/17425247.2010.513969. 20836625 PMC2945687

[ref310] Kataoka K.; Togawa H.; Harada A.; Yasugi K.; Matsumoto T.; Katayose S. Spontaneous Formation of Polyion Complex Micelles with Narrow Distribution from Antisense Oligonucleotide and Cationic Block Copolymer in Physiological Saline. Macromolecules 1996, 29, 8556–8557. 10.1021/ma961217+.

[ref311] Neu M.; Fischer D.; Kissel T. Recent Advances in Rational Gene Transfer Vector Design Based on Poly(Ethylene Imine) and Its Derivatives. J. Gene Med. 2005, 7, 992–1009. 10.1002/jgm.773. 15920783

[ref312] Vandamme T. F.; Brobeck L. Poly(Amidoamine) Dendrimers as Ophthalmic Vehicles for Ocular Delivery of Pilocarpine Nitrate and Tropicamide. J. Controlled Release 2005, 102, 23–38. 10.1016/j.jconrel.2004.09.015. 15653131

[ref313] Urbiola K.; Blanco-Fernández L.; Ogris M.; Rödl W.; Wagner E.; Tros de Ilarduya C. Novel PAMAM-PEG-Peptide Conjugates for siRNA Delivery Targeted to the Transferrin and Epidermal Growth Factor Receptors. J. Personalized Med. 2018, 8, 4 10.3390/jpm8010004. PMC5872078 29315261

[ref314] Singh P.; Gupta U.; Asthana A.; Jain N. K. Folate and Folate–PEG–PAMAM Dendrimers: Synthesis, Characterization, and Targeted Anticancer Drug Delivery Potential in Tumor Bearing Mice. Bioconjugate Chem. 2008, 19, 2239–2252. 10.1021/bc800125u. 18950215

[ref315] Tang Y.; Li Y.-B.; Wang B.; Lin R.-Y.; van Dongen M.; Zurcher D. M.; Gu X.-Y.; Banaszak Holl M. M.; Liu G.; Qi R. Efficient in Vitro siRNA Delivery and Intramuscular Gene Silencing Using PEG-Modified PAMAM Dendrimers. Mol. Pharmaceutics 2012, 9, 1812–1821. 10.1021/mp3001364. PMC3375318 22548294

[ref316] Piotrowski-Daspit A. S.; Kauffman A. C.; Bracaglia L. G.; Saltzman W. M. Polymeric Vehicles for Nucleic Acid Delivery. Adv. Drug Deliv. Rev. 2020, 156, 119–132. 10.1016/j.addr.2020.06.014. 32585159 PMC7736472

[ref317] Gaur S.; Wen Y.; Song J. H.; Parikh N. U.; Mangala L. S.; Blessing A. M.; Ivan C.; Wu S. Y.; Varkaris A.; Shi Y.; Lopez-Berestein G.; Frigo D. E.; Sood A. K.; Gallick G. E. Chitosan Nanoparticle-Mediated Delivery of miRNA-34a Decreases Prostate Tumor Growth in the Bone and Its Expression Induces Non-Canonical Autophagy. Oncotarget 2015, 6, 29161 10.18632/oncotarget.4971. 26313360 PMC4745718

[ref318] Cosco D.; Cilurzo F.; Maiuolo J.; Federico C.; Di Martino M. T.; Cristiano M. C.; Tassone P.; Fresta M.; Paolino D. Delivery of Mir-34a by Chitosan/Plga Nanoplexes for the Anticancer Treatment of Multiple Myeloma. Sci. Rep. 2015, 5, 17579. 26620594 10.1038/srep17579 PMC4665167

[ref319] Chen X.; Gu S.; Chen B.-F.; Shen W.-L.; Yin Z.; Xu G.-W.; Hu J.-J.; Zhu T.; Li G.; Wan C.; Ouyang H.-W.; Lee T.-L.; Chan W.-Y. Nanoparticle Delivery of Stable Mir-199a-5p Agomir Improves the Osteogenesis of Human Mesenchymal Stem Cells Via the Hif1a Pathway. Biomaterials 2015, 53, 239–250. 10.1016/j.biomaterials.2015.02.071. 25890723

[ref320] Köping-Höggård M.; Tubulekas I.; Guan H.; Edwards K.; Nilsson M.; Vårum K. M.; Artursson P. Chitosan as a Nonviral Gene Delivery System. Structure–Property Relationships and Characteristics Compared with Polyethylenimine in Vitro and after Lung Administration in Vivo. Gene Ther. 2001, 8, 1108–1121. 10.1038/sj.gt.3301492. 11526458

[ref321] Curtin C. M.; Tierney E. G.; McSorley K.; Cryan S.-A.; Duffy G. P.; O’Brien F. J. Combinatorial Gene Therapy Accelerates Bone Regeneration: Non-Viral Dual Delivery of Vegf and Bmp2 in a Collagen-Nanohydroxyapatite Scaffold. Adv. Healthcare Mater. 2015, 4, 223–227. 10.1002/adhm.201400397. 25125073

[ref322] Capito R. M.; Spector M. Collagen Scaffolds for Nonviral IGF-1 Gene Delivery in Articular Cartilage Tissue Engineering. Gene Ther. 2007, 14, 721–732. 10.1038/sj.gt.3302918. 17315042

[ref323] Cohen-Sacks H.; Elazar V.; Gao J.; Golomb A.; Adwan H.; Korchov N.; Levy R. J.; Berger M. R.; Golomb G. Delivery and Expression of pDNA Embedded in Collagen Matrices. J. Controlled Release 2004, 95, 309–320. 10.1016/j.jconrel.2003.11.001. 14980779

[ref324] Aoife M. O.; Mahony; Martin J. O.; Neill; Bruno M. D. C. G.; Raphael D.; John F. C.; Caitriona M. O.; Driscoll Cyclodextrins for Non-Viral Gene and siRNA Delivery. Pharmaceutical Nanotechnol. 2013, 1, 6–14. 10.2174/2211738511301010006.

[ref325] Chaturvedi K.; Ganguly K.; Kulkarni A. R.; Kulkarni V. H.; Nadagouda M. N.; Rudzinski W. E.; Aminabhavi T. M. Cyclodextrin-Based siRNA Delivery Nanocarriers: A State-of-the-Art Review. Expert Opin. Drug Deliv. 2011, 8, 1455–1468. 10.1517/17425247.2011.610790. 21867463

[ref326] Singh R. P.; Hidalgo T.; Cazade P.-A.; Darcy R.; Cronin M. F.; Dorin I.; O’Driscoll C. M.; Thompson D. Self-Assembled Cationic B-Cyclodextrin Nanostructures for siRNA Delivery. Mol. Pharmaceutics 2019, 16, 1358–1366. 10.1021/acs.molpharmaceut.8b01307. 30721074

[ref327] Mintzer M. A.; Simanek E. E. Nonviral Vectors for Gene Delivery. Chem. Rev. 2009, 109, 259–302. 10.1021/cr800409e. 19053809

[ref328] El-Sayed A.; Harashima H. Endocytosis of Gene Delivery Vectors: From Clathrin-Dependent to Lipid Raft-Mediated Endocytosis. Mol. Ther. 2013, 21, 1118–1130. 10.1038/mt.2013.54. 23587924 PMC3677298

[ref329] Bus T.; Traeger A.; Schubert U. S. The Great Escape: How Cationic Polyplexes Overcome the Endosomal Barrier. J. Mater. Chem. B 2018, 6, 6904–6918. 10.1039/C8TB00967H. 32254575

[ref330] Degors I. M. S.; Wang C.; Rehman Z. U.; Zuhorn I. S. Carriers Break Barriers in Drug Delivery: Endocytosis and Endosomal Escape of Gene Delivery Vectors. Acc. Chem. Res. 2019, 52, 1750–1760. 10.1021/acs.accounts.9b00177. 31243966 PMC6639780

[ref331] Liang K.; Such G. K.; Zhu Z.; Dodds S. J.; Johnston A. P. R.; Cui J.; Ejima H.; Caruso F. Engineering Cellular Degradation of Multilayered Capsules through Controlled Cross-Linking. ACS Nano 2012, 6, 10186–10194. 10.1021/nn3039353. 23121317

[ref332] Boisguérin P.; Deshayes S.; Gait M. J.; O’Donovan L.; Godfrey C.; Betts C. A.; Wood M. J. A.; Lebleu B. Delivery of Therapeutic Oligonucleotides with Cell Penetrating Peptides. Adv. Drug Deliv. Rev. 2015, 87, 52–67. 10.1016/j.addr.2015.02.008. 25747758 PMC7102600

[ref333] Kurrikoff K.; Langel Ü. Recent Cpp-Based Applications in Medicine. Expert Opin. Drug Deliv. 2019, 16, 1183–1191. 10.1080/17425247.2019.1665021. 31526146

[ref334] Boisguérin P.; Konate K.; Josse E.; Vivès E.; Deshayes S. Peptide-Based Nanoparticles for Therapeutic Nucleic Acid Delivery. Biomedicines 2021, 9, 583 10.3390/biomedicines9050583. 34065544 PMC8161338

[ref335] Huang Y.-W.; Lee H.-J.; Tolliver L. M.; Aronstam R. S. Delivery of Nucleic Acids and Nanomaterials by Cell-Penetrating Peptides: Opportunities and Challenges. BioMed. Res. Int. 2015, 2015, 834079 10.1155/2015/834079. 25883975 PMC4391616

[ref336] Vivès E.; Brodin P.; Lebleu B. A Truncated Hiv-1 Tat Protein Basic Domain Rapidly Translocates through the Plasma Membrane and Accumulates in the Cell Nucleus. J. Biol. Chem. 1997, 272, 16010–16017. 10.1074/jbc.272.25.16010. 9188504

[ref337] Yi A.; Sim D.; Lee Y.-J.; Sarangthem V.; Park R.-W. Development of Elastin-Like Polypeptide for Targeted Specific Gene Delivery in Vivo. J. Nanobiotechnol. 2020, 18, 15 10.1186/s12951-020-0574-z. PMC6969399 31952530

[ref338] Kasai H.; Inoue K.; Imamura K.; Yuvienco C.; Montclare J. K.; Yamano S. Efficient siRNA Delivery and Gene Silencing Using a Lipopolypeptide Hybrid Vector Mediated by a Caveolae-Mediated and Temperature-Dependent Endocytic Pathway. J. Nanobiotechnol. 2019, 17, 11 10.1186/s12951-019-0444-8. PMC6341701 30670041

[ref339] Guo F.; Fu Q.; Zhou K.; Jin C.; Wu W.; Ji X.; Yan Q.; Yang Q.; Wu D.; Li A.; Yang G. Matrix Metalloprotein-Triggered, Cell Penetrating Peptide-Modified Star-Shaped Nanoparticles for Tumor Targeting and Cancer Therapy. J. Nanobiotechnol. 2020, 18, 48 10.1186/s12951-020-00595-5. PMC7076984 32183823

[ref340] Koren E.; Torchilin V. P. Cell-Penetrating Peptides: Breaking through to the Other Side. Trends Mol. Med. 2012, 18, 385–393. 10.1016/j.molmed.2012.04.012. 22682515

[ref341] Langlet-Bertin B.; Leborgne C.; Scherman D.; Bechinger B.; Mason A. J.; Kichler A. Design and Evaluation of Histidine-Rich Amphipathic Peptides for siRNA Delivery. Pharm. Res. 2010, 27, 1426–1436. 10.1007/s11095-010-0138-2. 20393870

[ref342] Hsu T.; Mitragotri S. Delivery of siRNA and Other Macromolecules into Skin and Cells Using a Peptide Enhancer. Proc. Natl. Acad. Sci. U. S. A. 2011, 108, 15816–15821. 10.1073/pnas.1016152108. 21903933 PMC3179050

[ref343] Ryu Y. C.; Kim K. A.; Kim B. C.; Wang H.-M. D.; Hwang B. H. Novel Fusion Peptide-Mediated siRNA Delivery Using Self-Assembled Nanocomplex. J. Nanobiotechnol. 2021, 19, 44 10.1186/s12951-021-00791-x. PMC7881583 33579303

[ref344] Shukla R. S.; Qin B.; Cheng K. Peptides Used in the Delivery of Small Noncoding RNA. Mol. Pharmaceutics 2014, 11, 3395–3408. 10.1021/mp500426r. PMC4186677 25157701

[ref345] Simeoni F.; Morris M. C.; Heitz F.; Divita G. Insight into the Mechanism of the Peptide-Based Gene Delivery System Mpg: Implications for Delivery of siRNA into Mammalian Cells. Nucleic Acids Res. 2003, 31, 2717–2724. 10.1093/nar/gkg385. 12771197 PMC156720

[ref346] Fang B.; Guo H. Y.; Zhang M.; Jiang L.; Ren F. Z. The Six Amino Acid Antimicrobial Peptide bLFcin6 Penetrates Cells and Delivers siRNA. Febs J. 2013, 280, 1007–1017. 10.1111/febs.12093. 23241223

[ref347] Qin B.; Chen Z.; Jin W.; Cheng K. Development of Cholesteryl Peptide Micelles for siRNA Delivery. J. Controlled Release 2013, 172, 159–168. 10.1016/j.jconrel.2013.07.033. PMC3925743 23968830

[ref348] Guo J.; Cheng W. P.; Gu J.; Ding C.; Qu X.; Yang Z.; O’Driscoll C. Systemic Delivery of Therapeutic Small Interfering RNA Using a pH-Triggered Amphiphilic Poly-L-Lysine Nanocarrier to Suppress Prostate Cancer Growth in Mice. Eur. J. Pharm. Sci. 2012, 45, 521–532. 10.1016/j.ejps.2011.11.024. 22186295

[ref349] Warrant R. W.; Kim S.-H. A-Helix–Double Helix Interaction Shown in the Structure of a Protamine-Transfer RNA Complex and a Nucleoprotamine Model. Nature 1978, 271, 130–135. 10.1038/271130a0. 622153

[ref350] Schlake T.; Thess A.; Fotin-Mleczek M.; Kallen K. J. Developing mRNA-Vaccine Technologies. RNA Biol. 2012, 9, 1319–1330. 10.4161/rna.22269. 23064118 PMC3597572

[ref351] Rosi N. L.; Giljohann D. A.; Thaxton C. S.; Lytton-Jean A. K.; Han M. S.; Mirkin C. A. Oligonucleotide-Modified Gold Nanoparticles for Intracellular Gene Regulation. Science 2006, 312, 1027–30. 10.1126/science.1125559. 16709779

[ref352] Artiga A.; Serrano-Sevilla I.; De Matteis L.; Mitchell S. G.; de la Fuente J. M. Current Status and Future Perspectives of Gold Nanoparticle Vectors for siRNA Delivery. J. Mater. Chem. B 2019, 7, 876–896. 10.1039/C8TB02484G. 32255093

[ref353] Giljohann D. A.; Seferos D. S.; Prigodich A. E.; Patel P. C.; Mirkin C. A. Gene Regulation with Polyvalent siRNA–Nanoparticle Conjugates. J. Am. Chem. Soc. 2009, 131, 2072–2073. 10.1021/ja808719p. 19170493 PMC2843496

[ref354] Ding Y.; Jiang Z.; Saha K.; Kim C. S.; Kim S. T.; Landis R. F.; Rotello V. M. Gold Nanoparticles for Nucleic Acid Delivery. Mol. Ther.: J. Am. Soc. Gene Ther. 2014, 22, 1075–1083. 10.1038/mt.2014.30. PMC4048892 24599278

[ref355] Cutler J. I.; Auyeung E.; Mirkin C. A. Spherical Nucleic Acids. J. Am. Chem. Soc. 2012, 134, 1376–1391. 10.1021/ja209351u. 22229439

[ref356] Seferos D. S.; Giljohann D. A.; Hill H. D.; Prigodich A. E.; Mirkin C. A. Nano-Flares: Probes for Transfection and mRNA Detection in Living Cells. J. Am. Chem. Soc. 2007, 129, 15477–15479. 10.1021/ja0776529. 18034495 PMC3200543

[ref357] Wang S.; Qin L.; Yamankurt G.; Skakuj K.; Huang Z.; Chen P.-C.; Dominguez D.; Lee A.; Zhang B.; Mirkin C. A. Rational Vaccinology with Spherical Nucleic Acids. Proc. Natl. Acad. Sci. U. S. A. 2019, 116, 10473–10481. 10.1073/pnas.1902805116. 31068463 PMC6535021

[ref358] Plank C.; Zelphati O.; Mykhaylyk O. Magnetically Enhanced Nucleic Acid Delivery. Ten Years of Magnetofection—Progress and Prospects. Adv. Drug Deliv. Rev. 2011, 63, 1300–1331. 10.1016/j.addr.2011.08.002. 21893135 PMC7103316

[ref359] Mykhaylyk O.; Sanchez-Antequera Y.; Vlaskou D.; Cerda M. B.; Bokharaei M.; Hammerschmid E.; Anton M.; Plank C. Magnetic Nanoparticle and Magnetic Field Assisted siRNA Delivery in Vitro. Methods Mol. Biol. 2015, 1218, 53–106. 10.1007/978-1-4939-1538-5_5. 25319646

[ref360] Scherer F.; Anton M.; Schillinger U.; Henke J.; Bergemann C.; Krüger A.; Gänsbacher B.; Plank C. Magnetofection: Enhancing and Targeting Gene Delivery by Magnetic Force in Vitro and in Vivo. Gene Ther. 2002, 9, 102–109. 10.1038/sj.gt.3301624. 11857068

[ref361] Steitz B.; Hofmann H.; Kamau S. W.; Hassa P. O.; Hottiger M. O.; von Rechenberg B.; Hofmann-Amtenbrink M.; Petri-Fink A. Characterization of Pei-Coated Superparamagnetic Iron Oxide Nanoparticles for Transfection: Size Distribution, Colloidal Properties and DNA Interaction. J. Magn. Magn. Mater. 2007, 311, 300–305. 10.1016/j.jmmm.2006.10.1194.

[ref362] Tai M. F.; Chi K. M.; Lau K. H. W.; Baylink D. J.; Chen S. T. Generation of Magnetic Retroviral Vectors with Magnetic Nanoparticles. Rev. Adv. Mater. Sci. 2003, 5, 319–323.

[ref363] Ito A.; Takahashi T.; Kameyama Y.; Kawabe Y.; Kamihira M. Magnetic Concentration of a Retroviral Vector Using Magnetite Cationic Liposomes. Tissue Eng. C: Methods 2009, 15, 57–64. 10.1089/ten.tec.2008.0275. 18991483

[ref364] Namiki Y.; Namiki T.; Yoshida H.; Ishii Y.; Tsubota A.; Koido S.; Nariai K.; Mitsunaga M.; Yanagisawa S.; Kashiwagi H.; Mabashi Y.; Yumoto Y.; Hoshina S.; Fujise K.; Tada N. A Novel Magnetic Crystal–Lipid Nanostructure for Magnetically Guided in Vivo Gene Delivery. Nat. Nanotechnol. 2009, 4, 598–606. 10.1038/nnano.2009.202. 19734934

[ref365] Pan B.; Cui D.; Sheng Y.; Ozkan C.; Gao F.; He R.; Li Q.; Xu P.; Huang T. Dendrimer-Modified Magnetic Nanoparticles Enhance Efficiency of Gene Delivery System. Cancer Res. 2007, 67, 8156–8163. 10.1158/0008-5472.CAN-06-4762. 17804728

[ref366] Zheng X.; Lu J.; Deng L.; Xiong Y.; Chen J. Preparation and Characterization of Magnetic Cationic Liposome in Gene Delivery. Int. J. Pharm. 2009, 366, 211–217. 10.1016/j.ijpharm.2008.09.019. 18848871

[ref367] Chorny M.; Fishbein I.; Alferiev I.; Levy R. J. Magnetically Responsive Biodegradable Nanoparticles Enhance Adenoviral Gene Transfer in Cultured Smooth Muscle and Endothelial Cells. Mol. Pharmaceutics 2009, 6, 1380–1387. 10.1021/mp900017m. PMC3349935 19496618

[ref368] Yiu H. H.; McBain S. C.; Lethbridge Z. A.; Lees M. R.; Dobson J. Preparation and Characterization of Polyethylenimine-Coated Fe3o4-Mcm-48 Nanocomposite Particles as a Novel Agent for Magnet-Assisted Transfection. J. Biomed Mater. Res. A 2010, 92, 386–392. 10.1002/jbm.a.32363. 19191315

[ref369] Hom C.; Lu J.; Liong M.; Luo H.; Li Z.; Zink J. I.; Tamanoi F. Mesoporous Silica Nanoparticles Facilitate Delivery of siRNA to Shutdown Signaling Pathways in Mammalian Cells. Small 2010, 6, 1185–1190. 10.1002/smll.200901966. 20461725 PMC2953950

[ref370] Song H.; Yu M.; Lu Y.; Gu Z.; Yang Y.; Zhang M.; Fu J.; Yu C. Plasmid DNA Delivery: Nanotopography Matters. J. Am. Chem. Soc. 2017, 139, 18247–18254. 10.1021/jacs.7b08974. 29151352

[ref371] Bose S.; Tarafder S. Calcium Phosphate Ceramic Systems in Growth Factor and Drug Delivery for Bone Tissue Engineering: A Review. Acta Biomater. 2012, 8, 1401–1421. 10.1016/j.actbio.2011.11.017. 22127225 PMC3418064

[ref372] Levingstone T. J.; Herbaj S.; Redmond J.; McCarthy H. O.; Dunne N. J. Calcium Phosphate Nanoparticles-Based Systems for RNAi Delivery: Applications in Bone Tissue Regeneration. Nanomaterials (Basel) 2020, 10, 146 10.3390/nano10010146. PMC7023416 31947548

[ref373] Feray B. Gene Delivery by Hydroxyapatite and Calcium Phosphate Nanoparticles: A Review of Novel and Recent Applications. 10.5772/intechopen.71062 (accessed Aug 6, 2021).

[ref374] Wagner D. E.; Bhaduri S. B. Progress and Outlook of Inorganic Nanoparticles for Delivery of Nucleic Acid Sequences Related to Orthopedic Pathologies: A Review. Tissue Eng. B: Reviews 2012, 18, 1–14. 10.1089/ten.teb.2011.0081. 21707439

[ref375] van den Boorn J. G.; Schlee M.; Coch C.; Hartmann G. siRNA Delivery with Exosome Nanoparticles. Nat. Biotechnol. 2011, 29, 325–326. 10.1038/nbt.1830. 21478846

[ref376] Lu H.; Wang J.; Wang T.; Zhong J.; Bao Y.; Hao H. Recent Progress on Nanostructures for Drug Delivery Applications. J. Nanomater. 2016, 2016, 5762431 10.1155/2016/5762431.

[ref377] Khisamutdinov E. F.; Li H.; Jasinski D. L.; Chen J.; Fu J.; Guo P. Enhancing Immunomodulation on Innate Immunity by Shape Transition among RNA Triangle, Square and Pentagon Nanovehicles. Nucleic Acids Res. 2014, 42, 9996–10004. 10.1093/nar/gku516. 25092921 PMC4150753

[ref378] Hoeprich S.; Zhou Q.; Guo S.; Shu D.; Qi G.; Wang Y.; Guo P. Bacterial Virus Phi29 pRNA as a Hammerhead Ribozyme Escort to Destroy Hepatitis B Virus. Gene Ther. 2003, 10, 1258–1267. 10.1038/sj.gt.3302002. 12858191

[ref379] Geary C.; Rothemund P. W. K.; Andersen E. S. A Single-Stranded Architecture for Cotranscriptional Folding of RNA Nanostructures. Science 2014, 345, 799–804. 10.1126/science.1253920. 25124436

[ref380] Li H.; Lee T.; Dziubla T.; Pi F.; Guo S.; Xu J.; Li C.; Haque F.; Liang X.-J.; Guo P. RNA as a Stable Polymer to Build Controllable and Defined Nanostructures for Material and Biomedical Applications. Nano Today 2015, 10, 631–655. 10.1016/j.nantod.2015.09.003. 26770259 PMC4707685

[ref381] Rohovie M. J.; Nagasawa M.; Swartz J. R. Virus-Like Particles: Next-Generation Nanoparticles for Targeted Therapeutic Delivery. Bioeng. Transl. Med. 2017, 2, 43–57. 10.1002/btm2.10049. 29313023 PMC5689521

[ref382] Qian C.; Liu X.; Xu Q.; Wang Z.; Chen J.; Li T.; Zheng Q.; Yu H.; Gu Y.; Li S.; Xia N. Recent Progress on the Versatility of Virus-Like Particles. Vaccines 2020, 8, 139 10.3390/vaccines8010139. PMC7157238 32244935

[ref383] Keech C.; Albert G.; Cho I.; Robertson A.; Reed P.; Neal S.; Plested J. S.; Zhu M.; Cloney-Clark S.; Zhou H.; Smith G.; Patel N.; Frieman M. B.; Haupt R. E.; Logue J.; McGrath M.; Weston S.; Piedra P. A.; Desai C.; Callahan K.; Lewis M.; Price-Abbott P.; Formica N.; Shinde V.; Fries L.; Lickliter J. D.; Griffin P.; Wilkinson B.; Glenn G. M. Phase 1–2 Trial of a Sars-Cov-2 Recombinant Spike Protein Nanoparticle Vaccine. N. Engl. J. Med. 2020, 383, 2320–2332. 10.1056/NEJMoa2026920. 32877576 PMC7494251

[ref384] Bajaj S.; Banerjee M. Engineering Virus Capsids into Biomedical Delivery Vehicles: Structural Engineering Problems in Nanoscale. J. Biomed. Nanotechnol. 2015, 11, 53–69. 10.1166/jbn.2015.1959. 26301300

[ref385] Resch-Genger U.; Grabolle M.; Cavaliere-Jaricot S.; Nitschke R.; Nann T. Quantum Dots Versus Organic Dyes as Fluorescent Labels. Nat. Methods 2008, 5, 763–775. 10.1038/nmeth.1248. 18756197

[ref386] Wegner K. D.; Hildebrandt N. Quantum Dots: Bright and Versatile in Vitro and in Vivo Fluorescence Imaging Biosensors. Chem. Soc. Rev. 2015, 44, 4792–4834. 10.1039/C4CS00532E. 25777768

[ref387] Knipe J. M.; Peters J. T.; Peppas N. A. Theranostic Agents for Intracellular Gene Delivery with Spatiotemporal Imaging. Nano Today 2013, 8, 21–38. 10.1016/j.nantod.2012.12.004. 23606894 PMC3627379

[ref388] Wang Z.; Liu G.; Zheng H.; Chen X. Rigid Nanoparticle-Based Delivery of Anti-Cancer siRNA: Challenges and Opportunities. Biotechnology Advances 2014, 32, 831–843. 10.1016/j.biotechadv.2013.08.020. 24013011 PMC3947394

[ref389] Kim Y. D.; Park T. E.; Singh B.; Maharjan S.; Choi Y. J.; Choung P. H.; Arote R. B.; Cho C. S. Nanoparticle-Mediated Delivery of siRNA for Effective Lung Cancer Therapy. Nanomedicine (Lond) 2015, 10, 1165–88. 10.2217/nnm.14.214. 25929572

[ref390] Podesta J. E.; Al-Jamal K. T.; Herrero M. A.; Tian B.; Ali-Boucetta H.; Hegde V.; Bianco A.; Prato M.; Kostarelos K. Antitumor Activity and Prolonged Survival by Carbon-Nanotube-Mediated Therapeutic siRNA Silencing in a Human Lung Xenograft Model. Small 2009, 5, 1176–1185. 10.1002/smll.200990047. 19306454

[ref391] Al-Jamal K. T.; Toma F. M.; Yilmazer A.; Ali-Boucetta H.; Nunes A.; Herrero M.-A.; Tian B.; Eddaoudi A.; Al-Jamal W.T.; Bianco A.; Prato M.; Kostarelos K. Enhanced Cellular Internalization and Gene Silencing with a Series of Cationic Dendron-Multiwalled Carbon Nanotube:siRNA Complexes. FASEB J. 2010, 24, 4354–4365. 10.1096/fj.09-141036. 20647548

[ref392] Zhang Z.; Yang X.; Zhang Y.; Zeng B.; Wang S.; Zhu T.; Roden R. B.; Chen Y.; Yang R. Delivery of Telomerase Reverse Transcriptase Small Interfering RNA in Complex with Positively Charged Single-Walled Carbon Nanotubes Suppresses Tumor Growth. Clin. Cancer Res. 2006, 12, 4933–4939. 10.1158/1078-0432.CCR-05-2831. 16914582

[ref393] Wadhwa A.; Aljabbari A.; Lokras A.; Foged C.; Thakur A. Opportunities and Challenges in the Delivery of mRNA-Based Vaccines. Pharmaceutics 2020, 12, 102 10.3390/pharmaceutics12020102. PMC7076378 32013049

[ref394] Pardi N.; Tuyishime S.; Muramatsu H.; Kariko K.; Mui B. L.; Tam Y. K.; Madden T. D.; Hope M. J.; Weissman D. Expression Kinetics of Nucleoside-Modified mRNA Delivered in Lipid Nanoparticles to Mice by Various Routes. J. Controlled Release 2015, 217, 345–351. 10.1016/j.jconrel.2015.08.007. PMC4624045 26264835

[ref395] Zhang C.; Maruggi G.; Shan H.; Li J. Advances in mRNA Vaccines for Infectious Diseases. Front. Immunol. 2019, 10, 594 10.3389/fimmu.2019.00594. 30972078 PMC6446947

[ref396] Pardi N.; Hogan M. J.; Pelc R. S.; Muramatsu H.; Andersen H.; DeMaso C. R.; Dowd K. A.; Sutherland L. L.; Scearce R. M.; Parks R.; Wagner W.; Granados A.; Greenhouse J.; Walker M.; Willis E.; Yu J.-S.; McGee C. E.; Sempowski G. D.; Mui B. L.; Tam Y. K.; Huang Y.-J.; Vanlandingham D.; Holmes V. M.; Balachandran H.; Sahu S.; Lifton M.; Higgs S.; Hensley S. E.; Madden T. D.; Hope M. J.; Karikó K.; Santra S.; Graham B. S.; Lewis M. G.; Pierson T. C.; Haynes B. F.; Weissman D. Zika Virus Protection by a Single Low-Dose Nucleoside-Modified mRNA Vaccination. Nature 2017, 543, 248–251. 10.1038/nature21428. 28151488 PMC5344708

[ref397] Zakrewsky M.; Kumar S.; Mitragotri S. Nucleic Acid Delivery into Skin for the Treatment of Skin Disease: Proofs-of-Concept, Potential Impact, and Remaining Challenges. J. Controlled Release 2015, 219, 445–456. 10.1016/j.jconrel.2015.09.017. PMC5192040 26385169

[ref398] Golombek S.; Pilz M.; Steinle H.; Kochba E.; Levin Y.; Lunter D.; Schlensak C.; Wendel H. P.; Avci-Adali M. Intradermal Delivery of Synthetic mRNA Using Hollow Microneedles for Efficient and Rapid Production of Exogenous Proteins in Skin. Mol. Ther. - Nucleic Acids 2018, 11, 382–392. 10.1016/j.omtn.2018.03.005. 29858073 PMC5992458

[ref399] Van Lint S.; Goyvaerts C.; Maenhout S.; Goethals L.; Disy A.; Benteyn D.; Pen J.; Bonehill A.; Heirman C.; Breckpot K.; Thielemans K. Preclinical Evaluation of Trimix and Antigen mRNA-Based Antitumor Therapy. Cancer Res. 2012, 72, 1661–1671. 10.1158/0008-5472.CAN-11-2957. 22337996

[ref400] Sultana N.; Magadum A.; Hadas Y.; Kondrat J.; Singh N.; Youssef E.; Calderon D.; Chepurko E.; Dubois N.; Hajjar R. J.; Zangi L. Optimizing Cardiac Delivery of Modified mRNA. Mol. Ther. 2017, 25, 1306–1315. 10.1016/j.ymthe.2017.03.016. 28389322 PMC5474881

[ref401] Davis F. F. The Origin of Pegnology. Adv. Drug Deliv Rev. 2002, 54, 457–458. 10.1016/S0169-409X(02)00021-2. 12052708

[ref402] Zalipsky S. Chemistry of Polyethylene-Glycol Conjugates with Biologically-Active Molecules. Adv. Drug Deliv. Rev. 1995, 16, 157–182. 10.1016/0169-409X(95)00023-Z.

[ref403] Ikeda Y.; Nagasaki Y. Impacts of PEGylation on the Gene and Oligonucleotide Delivery System. J. Appl. Polym. Sci. 2014, 131, 40293 10.1002/app.40293.

[ref404] Bouchard P. R.; Hutabarat R. M.; Thompson K. M. Discovery and Development of Therapeutic Aptamers. Annu. Rev. Pharmacol. Toxicol. 2010, 50, 237–257. 10.1146/annurev.pharmtox.010909.105547. 20055704

[ref405] Haruta K.; Otaki N.; Nagamine M.; Kayo T.; Sasaki A.; Hiramoto S.; Takahashi M.; Hota K.; Sato H.; Yamazaki H. A Novel PEGylation Method for Improving the Pharmacokinetic Properties of Anti-Interleukin-17a RNA Aptamers. Nucleic Acid Ther. 2017, 27, 36–44. 10.1089/nat.2016.0627. 27827561 PMC5312557

