# Supplementary material for: The Progress and Promise of RNA Medicine—An Arsenal of Targeted Treatments
Source: J Med Chem. 2022 May 9;65(10):6975–7015. doi: 10.1021/acs.jmedchem.2c00024 (PMC9115888; doi:10.1021/acs.jmedchem.2c00024)
Supplement: Supplementary file 1 — jm2c00024_si_001.pdf [file jm2c00024_si_001.pdf]

Supporting Information

The progress and promise of RNA medicine—an arsenal of targeted treatments

Janet M. Sasso[†1], Barbara J. B. Ambrose[†1], Rumiana Tenchov[†1], Ruchira S. Datta[1], Matthew T. Basel[2], Robert K. DeLong[*3] and Qiongqiong Angela Zhou[*1]

[1] CAS, a division of the American Chemical Society, 2540 Olentangy River Rd, Columbus, OH 43202, USA
[2] College of Veterinary Medicine, Kansas State University, Manhattan, KS 66506, USA
[3] Nanotechnology Innovation Center Kansas State, Kansas State University, Manhattan, KS 66506, USA

[†]Authors J.M.S., B.J.B.A., and R.T. contributed equally to this paper.

*Corresponding authors:
Robert K. DeLong robertdelong@vet.k-state.edu
Qiongqiong Angela Zhou qzhou@cas.org

Contents:

1. Figure S1. Number of documents for various RNA types applied in medical studies in the years of 1995-2020, categorized by the type of diseases
2. Figure S2. Top companies with RNA therapeutics and vaccines in clinical trials
3. Figure S3. Frequencies of various types of modifications on RNA sequences and their distributions with respect to disease types obtained from the CAS Content Collection
4. Figure S4. Frequencies of various types of modifications on RNA sequences and their distributions with respect to specific diseases acquired from the CAS Content Collection
5. Figure S5. The co-occurrence of RNA modifications on the same sequences
6. Figure S6. Document numbers per year related to modified RNAs. Data were obtained from a SciFinder^{n} search
7. Figure S7. Modified and rare nucleic acid bases
8. Supplemental Table 1: Timeline and milestones of RNA research and development
9. Supplemental Table 2: RNA therapies and vaccines for various diseases in the development stages *(submitted separately as an Excel file)*

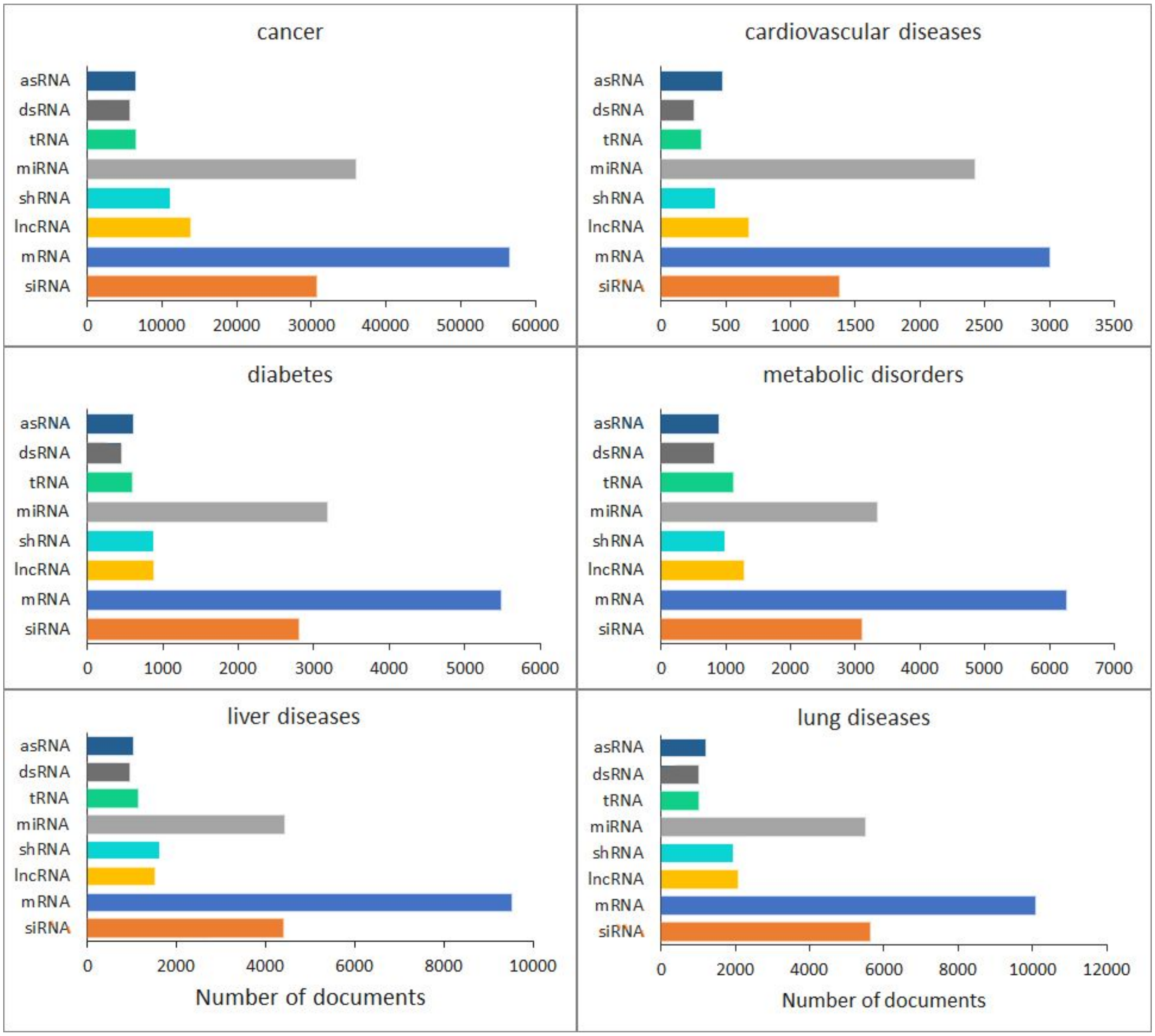

Figure S1. Number of documents for various RNA types applied in medical studies in the years of 1995-2020, categorized by the type of diseases

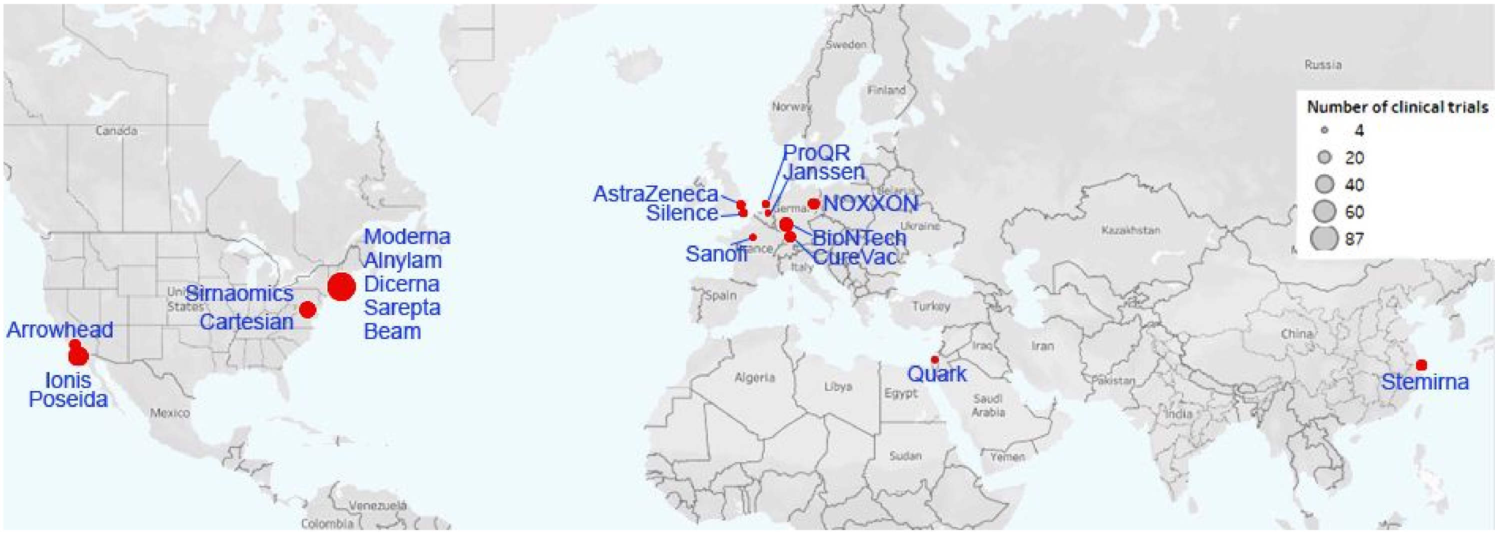

Figure S2. Top companies with RNA therapeutics and vaccines in clinical trials

Modification	**Grand total**	DNA-containing	deoxythymidine	deoxyguanosine	deoxyadenosine	deoxycytidine	deoxyuridine	2'-O-methylguanosine	2'-O-methyladenosine	2'-O-methyluridine	2'-O-methylcytidine	2'-O-methylcytidine	2'-O-methylinosine	5-methylcytidine	2'-O-(2-methoxyethyl)	modified base	modified uridine	modified guanosine	modified adenosine	modified cytidine	modified inosine	2'-deoxy	uncommon link	uncommon base	2'-fluoro	modified link	P-thio	3'->3'	5'->5'	2'->5'	5'-ester	3'-phosphorothioate	5'-phosphorothioate	5'-phosphoramidate
Proliferative disorders	8688	1002	977	242	198	179	39	461	454	306	301	195	7	81	36	733	98	123	92	95	1	360	337	99	308	362	360	279	34	6	237	6	7	1
Infection	4515	396	376	113	111	97	30	220	223	170	181	112	4	73	20	404	82	111	77	82	4	161	163	58	127	208	206	115	16	5	100	6	6	3
Inflammation	3911	345	330	124	83	77	13	213	216	147	147	86	5	39	17	352	52	82	52	50	1	171	170	51	148	159	159	128	18	1	125	2	2	4
Nervous system disease	3889	375	365	79	64	68	14	235	228	144	139	90	4	37	28	335	51	45	49	45	1	187	176	35	171	156	156	147	16	8	145	4	1	2
Digestive system disease	3792	437	430	89	84	79	11	215	211	150	150	66	5	42	23	320	53	46	45	47	1	164	138	31	144	158	158	118	9	4	109	2	1	2
Cardiovascular disease	3718	312	301	66	59	60	10	239	230	158	152	79	4	25	21	317	55	52	58	49	1	185	164	37	169	145	145	135	18	11	130	4	2	1
Eye disease	3328	311	305	23	21	23	7	281	281	73	73	22	4	5	3	299	16	16	17	14	1	256	251	28	245	54	54	236	14	8	229	1	0	0
Immune disease	2804	241	232	57	52	47	13	167	166	116	118	61	7	25	14	254	27	26	27	24	2	124	121	31	102	129	128	92	8	3	83	4	2	0
Urogenital system disease	2706	301	298	66	51	47	10	172	166	110	104	51	2	13	5	232	33	31	32	28	1	136	112	19	123	99	99	96	4	1	93	0	0	2
Skin disease	2678	252	244	59	49	52	6	167	164	109	104	58	4	23	6	227	22	23	23	21	1	131	118	36	111	110	110	101	13	5	87	1	1	1
Hematopoietic disorders	2655	278	272	56	50	51	9	159	156	106	102	62	5	28	14	238	23	21	22	20	1	123	110	25	109	121	121	100	8	0	84	0	0	0
Central nervous system disease	2513	230	227	48	41	41	6	157	153	95	92	51	4	25	19	216	29	27	28	27	1	128	112	26	114	103	103	102	10	6	91	4	1	0
Musculoskeletal disease	2323	178	172	42	33	36	8	143	142	110	108	58	5	20	13	202	40	40	39	37	1	110	100	33	91	103	102	78	11	0	67	0	0	0
Connective tissue disease	2311	177	171	60	51	44	6	129	129	107	97	60	6	30	17	189	43	39	41	39	1	102	87	30	91	101	100	65	15	4	60	1	1	1
Vascular disease	2296	183	179	38	35	37	4	161	154	95	90	50	4	14	10	211	33	31	33	27	1	130	108	22	120	85	85	95	5	2	85	2	1	0
Metabolic disorders	2136	189	184	45	47	45	5	109	105	104	102	64	2	33	31	177	44	39	42	35	0	75	46	13	74	126	125	33	5	5	37	4	1	2
Endocrine system disease	2098	199	193	42	39	37	4	132	125	97	93	45	4	21	12	178	27	26	26	23	1	96	80	20	82	100	100	69	1	0	59	1	2	1
Liver disease	2074	231	227	47	51	45	10	113	113	91	92	40	5	29	14	176	35	29	27	30	1	81	67	19	71	97	97	58	5	2	50	0	1	1
Reproductive system disease	1883	231	229	53	37	33	6	125	117	72	69	21	2	7	3	158	12	10	10	10	1	103	84	18	90	66	66	77	3	0	67	0	0	0
Degenerative disease	1860	116	110	28	23	26	5	82	79	53	52	104	5	43	38	173	64	63	63	59	1	54	89	10	52	86	86	34	6	3	72	2	2	3
Rheumatic diseases	1827	139	133	31	30	31	6	122	121	91	90	35	5	12	4	157	21	21	20	18	1	95	77	31	77	83	82	68	9	0	48	0	0	0
Respiratory system disease	1781	140	134	50	41	42	7	103	100	78	70	41	4	14	8	150	20	20	21	18	1	72	76	31	60	76	76	55	18	5	43	3	2	0
Autoimmune disease	1761	143	134	44	40	37	12	105	102	72	72	44	4	16	7	157	20	20	19	16	1	76	72	17	67	79	78	54	5	2	48	3	2	0
Pancreatic disease	1687	193	189	40	34	33	4	117	112	72	66	21	2	10	6	141	13	10	11	12	1	88	70	14	77	58	58	66	4	2	57	2	0	1
Geriatric disease	1655	154	152	9	7	6	2	147	148	31	31	7	1	1	0	150	4	4	5	2	0	137	132	11	134	25	25	128	6	3	126	1	0	0
Injury	1563	126	117	30	29	31	8	101	111	62	71	29	3	19	6	129	15	14	16	16	0	76	78	18	70	56	56	65	11	6	58	1	0	0
Neurodegenerative disease	1554	93	88	26	21	23	5	66	63	50	49	85	5	28	24	139	60	59	59	55	1	40	70	9	38	70	70	20	5	3	58	2	2	3
Diabetes mellitus	1549	140	136	37	34	33	4	91	85	74	70	41	4	19	10	127	24	23	23	20	1	64	51	12	57	81	81	41	1	0	39	1	2	1
Lung disease	1532	116	113	44	37	36	5	86	84	71	63	38	4	14	8	129	17	17	18	15	1	60	58	30	48	70	70	45	17	4	29	3	2	0
Heart disease	1381	95	93	27	24	21	5	82	78	73	71	40	4	12	11	116	33	33	36	30	1	59	39	6	56	73	73	24	4	4	32	3	0	0
Brain disease	1357	116	113	27	25	24	5	81	80	54	54	35	4	16	14	115	22	21	21	20	1	68	51	8	65	60	60	44	5	2	42	2	1	0
Blood disease	1329	116	113	29	25	29	5	86	86	64	61	27	2	13	8	116	10	10	10	7	0	66	54	11	59	64	64	50	2	0	46	0	0	0
Body fluid disorders	1083	92	90	10	10	9	3	88	88	27	28	11	1	1	1	95	8	8	10	9	0	79	80	11	74	23	23	74	2	1	68	1	0	0
Genetic disorders	987	86	85	14	10	14	2	62	60	47	45	28	2	15	12	86	14	13	17	13	0	46	31	3	44	54	54	23	0	0	28	2	1	0
Bladder disease	984	107	105	27	21	22	7	63	59	37	31	25	2	4	2	83	17	17	16	14	1	46	43	9	40	32	32	39	2	0	34	0	0	0

Figure S3. Frequencies of various types of modifications on RNA sequences obtained from the CAS Content Collection and their distributions with respect to disease types

Modification	Grand total	DNA-containing	deoxythymidine	deoxyguanosine	deoxyadenosine	deoxycytidine	2'-O-methylguanosine	2'-O-methyladenosine	2'-O-methyluridine	2'-O-methylcytidine	2'-deoxy	2'-fl	P-thio	3'->3'	5'-ester	5-methyluridine	2'-substituted	3'-ester	5-methylcytidine	3'-glycosylated	2'-O-(2-methoxyethyl)	5'-phosphate	modified link	modified base	modified uridine	modified guanosine	modified adenosine	modified cytidine	uncommon base	uncommon link	unavailable	stereoisomer
Age-related macular degeneration	1636	154	152	9	7	6	147	148	31	31	137	134	25	128	126	7	5	11	1	6	0	2	25	150	4	4	5	2	11	132	14	6
Pancreatic neoplasm	1382	173	171	37	31	31	96	91	57	51	71	60	45	58	46	15	15	1	7	1	3	2	45	115	7	6	6	7	14	61	18	3
Melanoma	1322	150	145	27	19	12	86	84	52	50	78	59	57	65	46	18	27	8	6	5	1	4	57	112	7	7	7	7	24	69	28	0
Atherosclerosis	1196	90	88	24	23	20	77	70	58	55	61	55	55	34	27	39	13	14	9	12	6	8	55	109	26	25	25	21	10	41	21	6
Glaucoma	1015	98	95	8	5	9	89	88	23	23	77	72	15	79	74	3	7	8	1	1	0	3	15	89	2	2	2	3	17	83	17	7
Hepatitis C	764	49	49	11	34	30	25	29	21	25	17	6	52	17	8	32	12	5	32	2	3	0	52	59	29	28	28	29	13	22	18	2
Hepatitis B	622	43	38	14	17	13	24	36	29	41	18	14	46	13	6	13	6	8	25	3	8	3	46	55	9	7	10	9	17	15	19	2
Non-small-cell lung carcinoma	605	63	63	10	9	8	48	46	18	17	39	34	12	36	33	4	5	0	1	0	1	1	12	59	3	3	3	3	5	38	6	1
Duchenne muscular dystrophy	562	7	7	4	4	4	19	19	19	19	6	6	22	1	35	49	2	1	8	1	5	0	22	56	42	43	43	39	0	38	38	3
Hypertension	530	44	43	13	11	13	31	27	19	16	26	22	20	19	17	12	6	3	4	2	3	2	20	40	11	10	11	9	5	23	9	4
Multiple myeloma	491	55	55	7	6	7	31	30	15	15	25	22	20	22	19	13	5	0	2	0	4	1	20	41	6	6	5	6	5	23	7	4
Amyotrophic lateral sclerosis	471	33	32	12	11	12	23	22	16	16	16	15	24	8	13	27	1	3	9	0	9	4	24	41	16	16	15	12	3	18	11	4
Retinitis pigmentosa	453	37	35	7	5	7	33	32	10	10	33	33	14	25	24	9	0	1	4	0	2	3	14	35	4	4	3	4	4	27	4	4
Influenza	450	61	59	43	11	10	13	10	12	8	5	5	8	2	3	9	0	2	2	1	0	6	8	58	8	42	8	9	0	7	4	1
Cystic fibrosis	418	41	40	16	15	7	27	27	22	15	21	19	11	19	10	6	4	1	2	1	1	2	11	36	2	2	2	2	9	20	11	2
Thrombosis	410	32	32	8	7	9	29	27	21	19	24	21	19	18	11	8	3	7	4	6	3	0	19	38	3	3	3	3	3	20	7	3
Acute myeloid leukemia	346	39	39	5	5	6	22	21	15	14	19	16	16	16	12	7	5	3	1	0	1	1	16	28	1	1	1	2	5	18	5	4
Nonalcoholic fatty liver disease	256	20	20	3	5	4	15	14	11	10	10	10	15	5	6	8	0	2	6	2	3	1	15	22	8	6	7	6	2	7	4	1
Solid neoplasm	240	35	35	5	8	5	15	14	13	11	8	7	9	6	6	1	2	1	1	1	1	3	9	20	0	0	0	0	2	7	5	2
Spinal muscular atrophy	248	3	3	0	0	0	4	4	4	4	3	1	22	3	3	26	2	1	20	1	20	0	22	28	7	7	7	7	2	9	9	0
Nonalcoholic steatohepatitis	191	12	12	2	4	3	12	10	10	8	7	7	11	3	4	5	0	1	3	1	1	1	11	15	7	5	6	5	1	5	3	1

Figure S4. Frequencies of various types of modifications on RNA sequences acquired from the CAS Content Collection and their distributions with respect to specific diseases

Modification	DNA-containing	PNA-containing	complex	copolymer	covalent bridge	homopolymer	labeled	metal complex	modified base	modified link	polyA-containing	radical ion	stereoisomer	uncommon base	uncommon link
DNA-containing		5	9	0	9	15	25	27	43408	31476	0	0	892	7541	9763
PNA-containing	5		0	0	0	0	0	0	17	0	0	0	1	2	15
complex	9	0		0	0	0	0	0	39	2	0	0	15	2	5
copolymer	0	0	0		0	1	0	0	1	0	0	0	0	0	0
covalent bridge	9	0	0	0		0	0	0	154	42	0	0	6	1	35
homopolymer	15	0	0	1	0		0	0	62	8	0	0	13	0	18
labeled	25	0	0	0	0	0		0	169	3	0	0	20	0	18
metal complex	27	0	0	0	0	0	0		97	5	0	0	7	2	18
modified base	43408	17	39	1	154	62	169	97		46270	5	31	1616	8344	14114
modified link	31476	0	2	0	42	8	3	5	46270		0	0	445	6072	6600
polyA-containing	0	0	0	0	0	0	0	0	5	0		0	0	1	5
radical ion	0	0	0	0	0	0	0	0	31	0	0		0	0	0
stereoisomer	892	1	15	0	6	13	20	7	1616	445	0	0		99	511
uncommon base	7541	2	2	0	1	0	0	2	8344	6072	1	0	99		11425
uncommon link	9763	15	5	0	35	18	18	18	14114	6600	5	0	511	11425	

Figure S5. The cooccurrence of RNA modifications on the same sequences

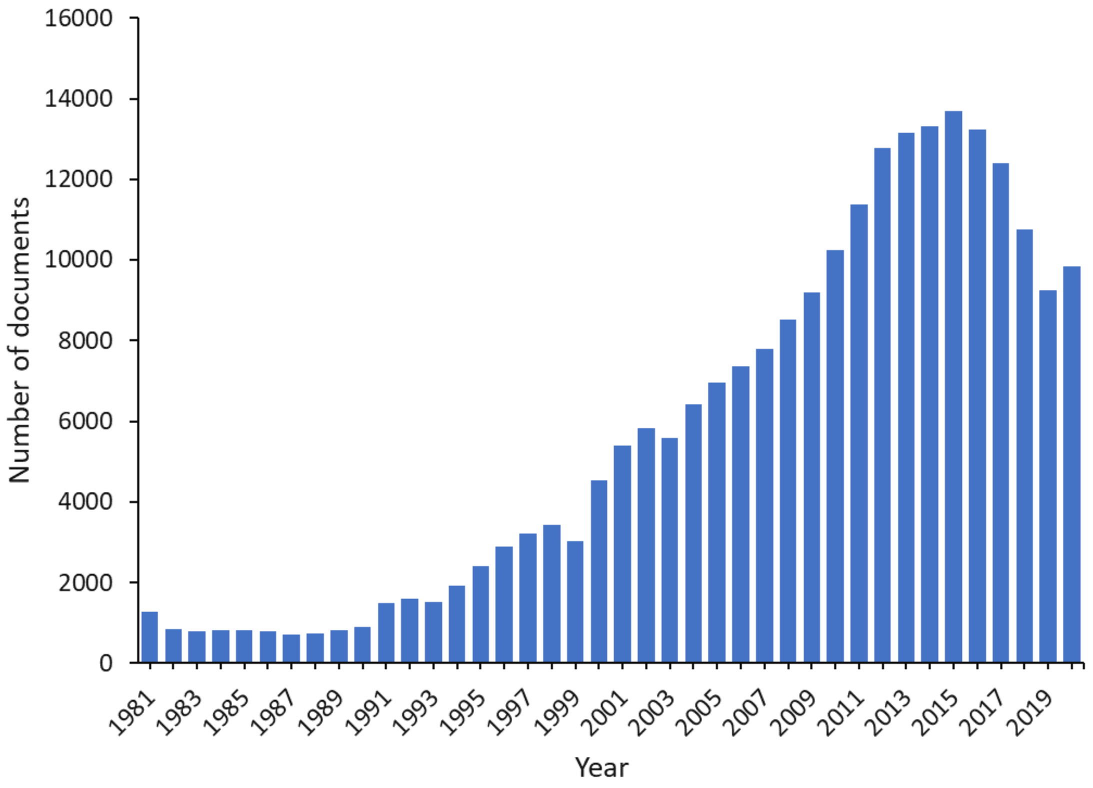

Figure S6. Document numbers per year related to modified RNAs. Data were obtained from a SciFinder^{n} search

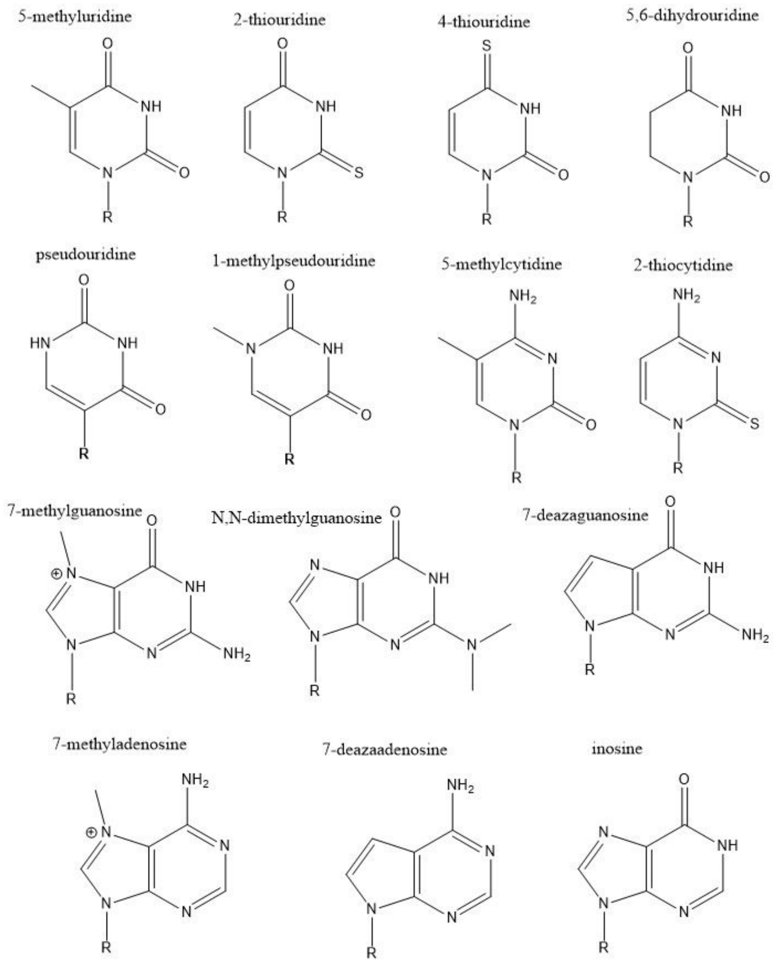

Figure S7. Modified and rare nucleosides. R = D-ribose.

Table S1. Timeline of the RNA-related advances and milestones

Year	RNA-related advances and milestones
1961	Discovery of messenger RNA [1]
1961	Elaboration of protamine-complexed RNA delivery to increase RNA infectivity [2]
1963	Discovering of interferon induction by foreign mRNA [3]
1969	First protein produced via in vitro translation of mRNA in living cells [4, 5]
1975	Discovery of mRNA cap (highly methylated modification of the 5' end) [6, 7]
1978	Liposome-entrapped mRNA delivery into mouse lymphocytes [8, 9]
1978	Inhibition of respiratory syncytial virus (RSV) using antisense oligonucleotides [10]
1984	mRNA synthesized in lab by SP6 in vitro transcription of cloned cDNAs by RNA polymerase [11]
1989	Development of cationic lipid-mediated mRNA delivery [12, 13]
1990	In vivo injection of mRNA into skeletal muscle of mice led to protein expression [14]
1992	Intrahypothalamic injection of vasopressin mRNA to rat brain corrects diabetes insipidus [15]
1993	First mRNA vaccine for infectious disease tested in mice (influenza) [16]
1995	First vaccination with mRNAs vaccine vector encoding cancer antigens tested in mice [17]
1996	Dendritic cells pulsed with mRNA found to be potent antigen-presenting cells [18]
1997	Merix: first mRNA-based company founded
1998	First antisense RNA drug Fomivirsen for cytomegalovirus retinitis approved [19]
1998	RNA interference discovered [20]
1999	First antitumor T cell response after injection with mRNA in vivo detected [21]
2001	Discovery of interferon induction by dsRNA activated TLR3 [22]
2001	Initiation of first clinical trial with mRNA using ex vivo transfected dendritic cells [23, 24]
2001	*Dicer* endoribonuclease facilitates activation of RNA-induced silencing complex (*RISC*), which is essential for RNA interference [25]
2002	First use of RNAi to destruct HCV in mice [26]
2003	siRNA as a therapeutic agent in mammals: RNAi targeting Fas protects mice from hepatitis [27]
2004	First RNA aptamer drug pegaptanib approved [28]
2004	Discovery of interferon induction by ssRNA-activated TLR7 and TLR8 [24]
2005	Pseudouridine modification found to stabilize RNA [29]

2006	Fire and Mello win Nobel Prize in Physiology or Medicine for their work on RNAi
2008	First clinical trial based on the direct application of mRNA (melanoma patients vaccinated with total tumor mRNA) [30]
2008	Development of zinc finger mRNA for gene editing [31]
2009	Direct injection (gene gun) delivery of mRNA for human cancer immunotherapy [30]
2009	Adoptive immunotherapy using injection of T cells transfected ex vivo with CAR mRNA [32, 33]
2010	First clinical trial based on RNAi demonstrating that siRNA administration can result in inhibition of a specific target gene in human [34]
2011	Protein replacement preclinical study: nucleoside-modified mRNA corrects disease [35]
2011	Development of targeted genome editing using TALEN mRNA for gene editing [36]
2012	First LNP-formulated mRNA vaccine tested in mice [37]
2013	Development of CRISPR–Cas9 mRNA for gene editing [38]
2013	*Science* magazine proclaims cancer immunotherapy as breakthrough of the year
2013	First mRNA vaccine for infectious disease in clinical trial (rabies) [39]
2015	First LNP-formulated mRNA vaccine in clinical trial (influenza) [40, 41]
2017	Personalized neo-epitope mRNA anticancer vaccine tested in patients with melanoma [42]
2018	First siRNA drug Patisiran approved [43]
2020	Charpentier and Doudna win a Nobel Prize in Chemistry for their work on CRISPR-Cas9
2020	mRNA vaccines for COVID-19 received EUA [44, 45]
2021	mRNA vaccine for COVID-19 received final FDA approval [46]

References

1. Brenner, S., Meselson, M.,Jacob, F., An unstable intermediate carrying information from genes to ribosomes for protein synthesis. *Nature* **1961,** *190*, 576-+.
2. Smull, C. E., Mallette, M. F.,Ludwig, E. H., The use of basic proteins to increase the infectivity of enterovirus ribonucleic acid. *Biochemical and Biophysical Research Communications* **1961,** *5*, 247-249.
3. Isaacs, A., Cox, R. A.,Rotem, Z., Foreign nucleic acids as the stimulus to make interferon. *Lancet* **1963,** *2*, 113-6.
4. Lockard, R. E.,Lingrel, J. B., Synthesis of mouse hemoglobin beta-chains in a rabbit reticulocyte cell-free system programmed with mouse reticulocyte 9S RNA. *Biochemical and Biophysical Research Communications* **1969,** *37*, 204-&.

5. Gurdon, J. B., Lane, C. D., Woodland, H. R.,Marbaix, G., Use of frog eggs and oocytes for study of messenger RNA and its translation in living cells *Nature* **1971,** *233*, 177-&.
6. Muthukrishnan, S., Both, G. W., Furuichi, Y.,Shatkin, A. J., 5'-Terminal 7-methylguanosine in eukaryotic messenger-RNA is required for translation. *Nature* **1975,** *255*, 33-37.
7. Furuichi, Y.,Miura, K. I., Blocked structure at 5' terminus of messenger-RNA from cytoplasmic polyhedrosis-virus. *Nature* **1975,** *253*, 374-375.
8. Papahadjopoulos, D., Vail, W. J., Jacobson, K.,Poste, G., Cochleate lipid cylinders: formation by fusion of unilamellar lipid vesicles. *Biochim Biophys Acta* **1975,** *394*, 483-91.
9. Dimitriadis, G. J., Translation of rabbit globin mRNA introduced by liposomes into mouse lymphocytes. *Nature* **1978,** *274*, 923-924.
10. Zamecnik, P. C.,Stephenson, M. L., Inhibition of Rous sarcoma virus replication and cell transformation by a specific oligodeoxynucleotide. *Proceedings of the National Academy of Sciences of the United States of America* **1978,** *75*, 280-284.
11. Krieg, P. A.,Melton, D. A., Functional messenger-RNAs are produced by SP6 invitro transcription of cloned cDNAs. *Nucleic Acids Research* **1984,** *12*, 7057-7070.
12. Felgner, P. L., Gadek, T. R., Holm, M., Roman, R., Chan, H. W., Wenz, M., Northrop, J. P., Ringold, G. M.,Danielsen, M., Lipofection - A Highly Efficient, Lipid-Mediated DNA-Transfection Procedure. *Proceedings of the National Academy of Sciences of the United States of America* **1987,** *84*, 7413-7417.
13. Malone, R. W., Felgner, P. L.,Verma, I. M., Cationic liposome-mediated RNA transfection. *Proceedings of the National Academy of Sciences of the United States of America* **1989,** *86*, 6077-6081.
14. Wolff, J. A., Malone, R. W., Williams, P., Chong, W., Acsadi, G., Jani, A.,Felgner, P. L., Direct gene transfer into mouse muscle in vivo. *Science* **1990,** *247*, 1465-8.
15. Jirikowski, G. F., Sanna, P. P., Maciejewskilenoir, D.,Bloom, F. E., Reversal of diabetes-insipidus in Brattleboro rats - intrahypothalamic injection of vasopressin messenger-RNA. *Science* **1992,** *255*, 996-998.
16. Martinon, F., Krishnan, S., Lenzen, G., Magne, R., Gomard, E., Guillet, J. G., Levy, J. P.,Meulien, P., INDUCTION OF VIRUS-SPECIFIC CYTOTOXIC T-LYMPHOCYTES IN-VIVO BY LIPOSOME-ENTRAPPED MESSENGER-RNA. *European Journal of Immunology* **1993,** *23*, 1719-1722.
17. Conry, R. M., Lobuglio, A. F., Wright, M., Sumerel, L., Pike, M. J., Feng, J. N., Benjamin, R., Lu, D.,Curiel, D. T., Characterization of a messenger-RNA polynucleotide vaccine vector. *Cancer Research* **1995,** *55*, 1397-1400.
18. Boczkowski, D., Nair, S. K., Snyder, D.,Gilboa, E., Dendritic cells pulsed with RNA are potent antigen-presenting cells in vitro and in vivo. *J Exp Med* **1996,** *184*, 465-72.
19. Piascik, P., Fomiversen sodium approved to treat CMV retinitis. *J Am Pharm Assoc (Wash)* **1999,** *39*, 84-5.
20. Fire, A., Xu, S. Q., Montgomery, M. K., Kostas, S. A., Driver, S. E.,Mello, C. C., Potent and specific genetic interference by double-stranded RNA in Caenorhabditis elegans. *Nature* **1998,** *391*, 806-811.
21. Zhou, W. Z., Hoon, D. S., Huang, S. K., Fujii, S., Hashimoto, K., Morishita, R.,Kaneda, Y., RNA melanoma vaccine: induction of antitumor immunity by human glycoprotein 100 mRNA immunization. *Hum Gene Ther* **1999,** *10*, 2719-24.
22. Alexopoulou, L., Holt, A. C., Medzhitov, R.,Flavell, R. A., Recognition of double-stranded RNA and activation of NF-κB by Toll-like receptor 3. *Nature* **2001,** *413*, 732-738.
23. Heiser, A., Coleman, D., Dannull, J., Yancey, D., Maurice, M. A., Lallas, C. D., Dahm, P., Niedzwiecki, D., Gilboa, E.,Vieweg, J., Autologous dendritic cells transfected with prostate-specific antigen RNA stimulate CTL responses against metastatic prostate tumors. *J Clin Invest* **2002,** *109*, 409-17.
24. Diebold, S. S., Kaisho, T., Hemmi, H., Akira, S.,Reis e Sousa, C., Innate antiviral responses by means of TLR7-mediated recognition of single-stranded RNA. *Science* **2004,** *303*, 1529-31.

25. Bernstein, E., Caudy, A. A., Hammond, S. M.,Hannon, G. J., Role for a bidentate ribonuclease in the initiation step of RNA interference. *Nature* **2001,** *409*, 363-366.
26. McCaffrey, A. P., Meuse, L., Pham, T. T., Conklin, D. S., Hannon, G. J.,Kay, M. A., RNA interference in adult mice. *Nature* **2002,** *418*, 38-9.
27. Song, E., Lee, S. K., Wang, J., Ince, N., Ouyang, N., Min, J., Chen, J., Shankar, P.,Lieberman, J., RNA interference targeting Fas protects mice from fulminant hepatitis. *Nat Med* **2003,** *9*, 347-51.
28. Gragoudas, E. S., Adamis, A. P., Cunningham, E. T., Jr., Feinsod, M.,Guyer, D. R., Pegaptanib for neovascular age-related macular degeneration. *N Engl J Med* **2004,** *351*, 2805-16.
29. Kariko, K., Buckstein, M., Ni, H. P.,Weissman, D., Suppression of RNA recognition by Toll-like receptors: The impact of nucleoside modification and the evolutionary origin of RNA. *Immunity* **2005,** *23*, 165-175.
30. Weide, B., Pascolo, S., Scheel, B., Derhovanessian, E., Pflugfelder, A., Eigentler, T. K., Pawelec, G., Hoerr, I., Rammensee, H.-G.,Garbe, C., Direct Injection of Protamine-protected mRNA: Results of a Phase 1/2 Vaccination Trial in Metastatic Melanoma Patients. *Journal of Immunotherapy* **2009,** *32*.
31. Doyon, Y., McCammon, J. M., Miller, J. C., Faraji, F., Ngo, C., Katibah, G. E., Amora, R., Hocking, T. D., Zhang, L., Rebar, E. J., Gregory, P. D., Urnov, F. D.,Amacher, S. L., Heritable targeted gene disruption in zebrafish using designed zinc-finger nucleases. *Nat Biotechnol* **2008,** *26*, 702-8.
32. Yoon, S. H., Lee, J. M., Cho, H. I., Kim, E. K., Kim, H. S., Park, M. Y.,Kim, T. G., Adoptive immunotherapy using human peripheral blood lymphocytes transferred with RNA encoding Her-2/neu-specific chimeric immune receptor in ovarian cancer xenograft model. *Cancer Gene Therapy* **2009,** *16*, 489-497.
33. Rabinovich, P. M., Komarovskaya, M. E., Wrzesinski, S. H., Alderman, J. L., Budak-Alpdogan, T., Karpikov, A., Guo, H., Flavell, R. A., Cheung, N.-K., Weissman, S. M.,Bahceci, E., Chimeric receptor mRNA transfection as a tool to generate antineoplastic lymphocytes. *Human gene therapy* **2009,** *20*, 51-61.
34. Davis, M. E., Zuckerman, J. E., Choi, C. H. J., Seligson, D., Tolcher, A., Alabi, C. A., Yen, Y., Heidel, J. D.,Ribas, A., Evidence of RNAi in humans from systemically administered siRNA via targeted nanoparticles. *Nature* **2010,** *464*, 1067-1070.
35. Kormann, M. S. D., Hasenpusch, G., Aneja, M. K., Nica, G., Flemmer, A. W., Herber-Jonat, S., Huppmann, M., Mays, L. E., Illenyi, M., Schams, A., Griese, M., Bittmann, I., Handgretinger, R., Hartl, D., Rosenecker, J.,Rudolph, C., Expression of therapeutic proteins after delivery of chemically modified mRNA in mice. *Nature Biotechnology* **2011,** *29*, 154-157.
36. Wood, A. J., Lo, T. W., Zeitler, B., Pickle, C. S., Ralston, E. J., Lee, A. H., Amora, R., Miller, J. C., Leung, E., Meng, X., Zhang, L., Rebar, E. J., Gregory, P. D., Urnov, F. D.,Meyer, B. J., Targeted genome editing across species using ZFNs and TALENs. *Science* **2011,** *333*, 307.
37. Geall, A. J., Verma, A., Otten, G. R., Shaw, C. A., Hekele, A., Banerjee, K., Cu, Y., Beard, C. W., Brito, L. A., Krucker, T., O'Hagan, D. T., Singh, M., Mason, P. W., Valiante, N. M., Dormitzer, P. R., Barnett, S. W., Rappuoli, R., Ulmer, J. B.,Mandl, C. W., Nonviral delivery of self-amplifying RNA vaccines. *Proceedings of the National Academy of Sciences of the United States of America* **2012,** *109*, 14604-14609.
38. Hwang, W. Y., Fu, Y., Reyon, D., Maeder, M. L., Tsai, S. Q., Sander, J. D., Peterson, R. T., Yeh, J. R. J.,Joung, J. K., Efficient genome editing in zebrafish using a CRISPR-Cas system. *Nature Biotechnology* **2013,** *31*, 227-229.
39. Alberer, M., Gnad-Vogt, U., Hong, H. S., Mehr, K. T., Backert, L., Finak, G., Gottardo, R., Bica, M. A., Garofano, A., Koch, S. D., Fotin-Mleczek, M., Hoerr, I., Clemens, R.,von Sonnenburg, F., Safety and immunogenicity of a mRNA rabies vaccine in healthy adults: an open-label, non-randomised, prospective, first-in-human phase 1 clinical trial. *Lancet* **2017,** *390*, 1511-1520.
40. Bahl, K., Senn, J. J., Yuzhakov, O., Bulychev, A., Brito, L. A., Hassett, K. J., Laska, M. E., Smith, M., Almarsson, Ö., Thompson, J., Ribeiro, A. M., Watson, M., Zaks, T.,Ciaramella, G., Preclinical and Clinical

Demonstration of Immunogenicity by mRNA Vaccines against H10N8 and H7N9 Influenza Viruses. *Molecular therapy : the journal of the American Society of Gene Therapy* **2017,** *25*, 1316-1327.
41. Safety, Tolerability, and Immunogenicity of VAL-506440 in Healthy Adult Subjects. ClinicalTrials.gov, 2017.
42. Guo, Y., Lei, K.,Tang, L., Neoantigen Vaccine Delivery for Personalized Anticancer Immunotherapy. *Frontiers in Immunology* **2018,** *9*.
43. Adams, D., Gonzalez-Duarte, A., O'Riordan, W. D., Yang, C. C., Ueda, M., Kristen, A. V., Tournev, I., Schmidt, H. H., Coelho, T., Berk, J. L., Lin, K. P., Vita, G., Attarian, S., Planté-Bordeneuve, V., Mezei, M. M., Campistol, J. M., Buades, J., Brannagan, T. H., 3rd, Kim, B. J., Oh, J., Parman, Y., Sekijima, Y., Hawkins, P. N., Solomon, S. D., Polydefkis, M., Dyck, P. J., Gandhi, P. J., Goyal, S., Chen, J., Strahs, A. L., Nochur, S. V., Sweetser, M. T., Garg, P. P., Vaishnaw, A. K., Gollob, J. A.,Suhr, O. B., Patisiran, an RNAi Therapeutic, for Hereditary Transthyretin Amyloidosis. *N Engl J Med* **2018,** *379*, 11-21.
44. Vaccines and Related Biological Products Advisory Committee Meeting. Moderna COVID-19 Vaccine. FDA Briefing Document. https://www.fda.gov/media/144434/download (accessed December 22, 2020).
45. Emergency use authorization (EUA) of the Pfizer-BioNTech COVID-19 vaccine to prevent coronavirus disease 2019 (COVID-19) in individuals 16 years of age and older. https://www.fda.gov/media/144414/download (accessed December 22, 2020).
46. FDA Approves First COVID-19 Vaccine. https://www.fda.gov/news-events/press-announcements/fda-approves-first-covid-19-vaccine (accessed Dec 27, 2021).
